# Main-chain stiff-stilbene photoswitches in solution, in bulk, and at surfaces†

**DOI:** 10.1039/d4sc06470d

**Published:** 2024-11-19

**Authors:** Naoki Kaneda, Keiichi Imato, Ayane Sasaki, Ryo Tanaka, Ichiro Imae, Toyoaki Hirata, Takuya Matsumoto, Yousuke Ooyama

**Affiliations:** a Applied Chemistry Program, Graduate School of Advanced Science and Engineering, Hiroshima University 1-4-1 Kagamiyama Higashihiroshima 739-8527 Japan kimato@hiroshima-u.ac.jp yooyama@hiroshima-u.ac.jp; b Department of Frontier Fiber Technology and Science, Graduate School of Engineering, University of Fukui 3-9-1 Bunkyo Fukui 910-8507 Japan; c Department of Chemical Science and Engineering, Graduate School of Engineering, Kobe University 1-1 Rokkodaicho, Nada Kobe 657-8501 Japan

## Abstract

Molecular photoswitches have been incorporated into polymer backbones to control the macromolecular conformations by structural changes of the main-chain photoswitches. However, previous photoswitches installed in the main chains are thermolabile, which precludes deep understanding, precise regulation, and practical applications of the macromolecular conformational changes. Herein, we focus on sterically hindered stiff stilbene (HSS), an emerging photoswitch offering large structural changes in isomerization between the thermally bistable *E* and *Z* isomers, and disclose the chemistry of main-chain HSS photoswitches in solution, in bulk, and at thin film surfaces. We synthesize and investigate three types of linear polymers with different chemical linkages between HSS repeating units, polyurethane, polyester, and polyene. The polymer conformations in solution, *i.e.*, hydrodynamic volume, are reversibly photocontrollable in a precise manner by the *E*/*Z* ratio. Furthermore, the nanoscopic conformational transformations are amplified to macroscopic photoswitching of the solution transmittance and the surface wettability synergistically with changes between interchain and intrachain hydrogen bonding in the polyurethanes. Additionally, the *Z*-to-*E* photoisomerization yields in the glassy state are above 70%, comparable to those in solution, and extraordinarily high despite the restricted molecular mobility. The findings of this study will pave the way for practical and unconventional applications of smart polymer systems based on photoswitches.

## Abstract 2

We report the chemistry of main-chain photoswitches of sterically hindered stiff stilbene, which offers large structural changes and high thermal stability, in solution, in bulk, and at thin film surfaces.

## Graphical abstract

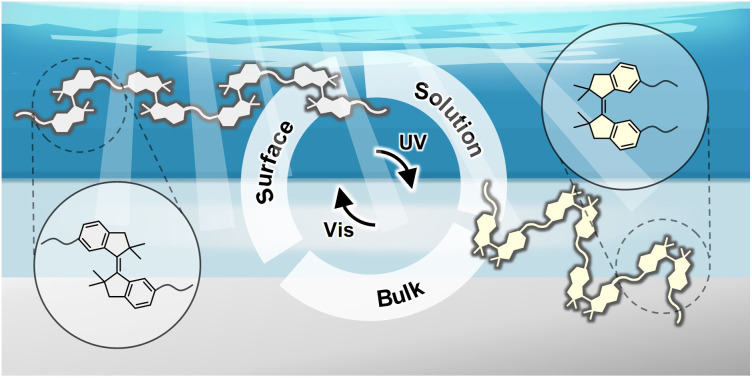

## Introduction

Molecular switches reversibly interconvert between two or more thermodynamically (meta)stable states in response to external stimulation with accompanying changes of their structures and properties and have been widely employed in molecular machines and stimuli-responsive (smart) materials.^1–4^ In particular, photoresponsive switches are commonly used owing to unique advantages of photostimulation including high spatiotemporal resolution, easy and precise control of wavelength and intensity, and no production of chemical waste.^5–12^ Even now, a large number of photoswitches with fascinating properties including near-infrared excitation and quantitative photoisomerization are newly reported.^13–19^ Recently, photocontrol of polymer conformations by focusing on structural changes of photoswitches and incorporating them into the polymer backbones has attracted considerable interest^20–24^ due to the wide variety of promising applications such as actuators,^25–31^ single-chain nanoparticles,^32,33^ and photoinduced transitions.^34–40^ Conformational changes of single polymer chains in solution induced by main-chain photoswitches and their impact on bulk properties have been studied predominantly by using azobenzene (AB),^20–26,29–31,33–39^ which is the most representative photoswitch and characterized by large structural variations in the *E*–*Z* isomerization.^41–44^ However, the *Z* isomer is so thermally unstable that the thermal *Z*-to-*E* isomerization rapidly proceeds even at room temperature (RT) with a half-life (*t*_1/2_) of *ca.* 1 day, precluding detailed investigation, precise control, and practical use of the macromolecular conformational changes. Except for AB, main-chain diarylethene,^45^ α-bisimine,^46–49^ thioindigo,^50^ and spiropyran^51^ photoswitches have been demonstrated to transform polymer conformations. Nevertheless, diarylethene is thermally stable but generates only small structural changes, which limit polymer backbones to rigid π-conjugated chains for the conformational changes,^45^ while α-bisimine, thioindigo, and spiropyran offer moderate or large structural changes but are thermally unstable similar to AB.^46–51^ In other words, these photoswitches cannot combine both large structural changes and high thermal stability. In this context, very recently, photocontrol of conformations of single polymer chains in solution, *i.e.*, hydrodynamic volume, and resulting changes in bulk physical properties, *i.e.*, glass transition temperatures (*T*_g_s), were investigated in detail by using hydrazone photoswitches offering both large structural changes and high thermal stability.^52^

**Fig. 1:**
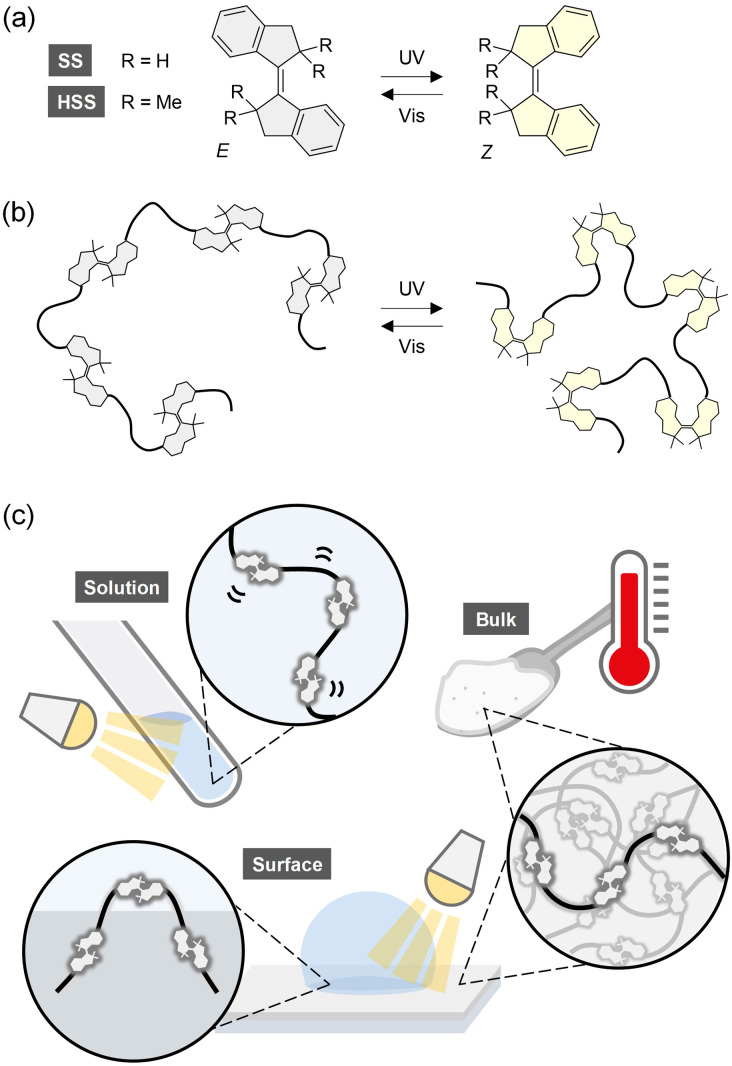
(a) Photoisomerization of SS and HSS. (b) Conformational changes of a single polymer chain by photoisomerization of the main-chain HSS. (c) Overview of this study.

In the present study, we focus on an emerging photoswitch based on stiff stilbene (SS) (Fig. 1a), which grabs the spotlight due to large structural changes in the *E*–*Z* isomerization and high thermal stability of the metastable *Z* isomer^53^ similar to hydrazone. Although various intriguing applications of SS-based photoswitches have been considered,^54–66^ there are only four papers on their polymers.^67–70^ Multiple *E*-SS molecules were incorporated into a main chain as a repeating unit by ring-opening metathesis polymerization of a strained *E*-SS macrocycle^67^ or into side chains by reversible addition–fragmentation chain transfer copolymerization of *E*-SS and other monomers.^68^ A single *Z* isomer of SS was located at different positions of a polymer backbone by reactions between a difunctional *Z*-SS and two monofunctional polymers^69^ or by atom transfer radical polymerization from a *Z*-SS initiator with two initiating groups.^70^ However, photoisomerization of the introduced SS molecules was observed only in the side chains,^68^ and the impact of their photoisomerization on the polymer structures and properties has not been investigated at all. One of the main reasons is the high reactivity of the central C=C bond with Grubbs catalysts and radicals in the above polymerizations.^67,70^ On the other hand, we previously found that sterically hindered SS (HSS) with four methyl groups modified around the central C=C bond^71–74^ (Fig. 1a) was unreactive to radicals and capable of producing high-molecular-weight and narrow-dispersity polymers with the mid-chain HSS functionality in a controlled manner, while maintaining the thermal stability of the *Z* isomer (*t*_1/2_ = *ca.* 1000 years at RT).^70,72^

Here we synthesize linear polymers containing multiple HSS photoswitches in the backbones as repeating units by using three types of step-growth polymerization and investigate the photoisomerization, macromolecular conformational changes, and macroscopic reflection in solution, in bulk, and at thin film surfaces (Fig. 1b and c). To the best of our knowledge, this is the first study that quantitatively examines both directions of photoisomerization of main-chain photoswitches in the bulk state (in thin films) and amplifies the nanoscopic polymer conformational changes to the macroscopic scale in solution and at thin film surfaces. Therefore, the findings of this study will contribute significantly to the fundamental understanding and practical applications of photoresponsive polymeric systems based on photoswitches.

## Results and discussion

### Synthesis of monomers and polymers

**Fig. 2:**
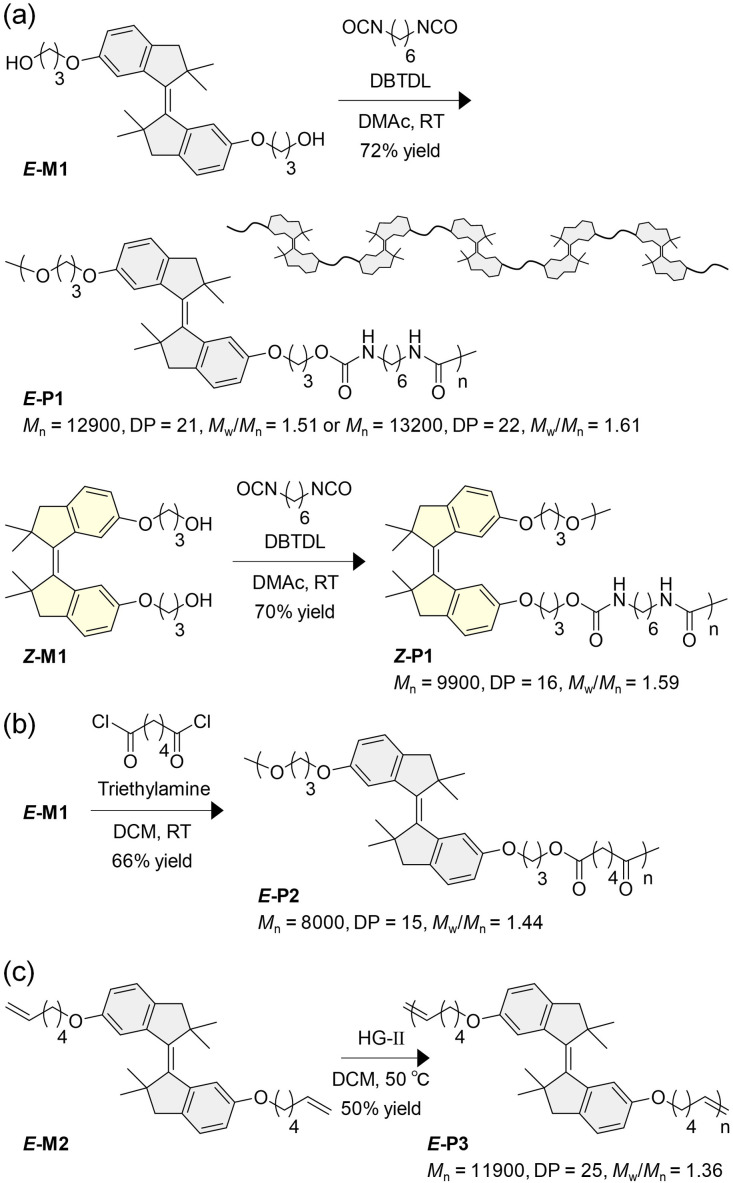
Polymerization for linear polymers with main-chain HSS functionality: (a) polyaddition for polyurethanes *E*-P1 and *Z*-P1, (b) polycondensation for polyester *E*-P2, and (c) ADMET polymerization for polyene *E*-P3.

We synthesized three HSS monomers named *E*-M1 and *Z*-M1, *E* and *Z* isomers with two hydroxy groups, and *E*-M2, an *E* isomer with two vinyl groups (Fig. 2). The polymerizable groups were attached at the positions that maximize conformational variations of polymer chains.^70,72^ We adopted three types of step-growth polymerization, polyaddition, polycondensation, and acyclic diene metathesis (ADMET) polymerization, for the first time for SS-based photoswitches to produce structurally similar but chemically distinct linear polymers, polyurethanes *E*-P1 and *Z*-P1, polyester *E*-P2, and polyene *E*-P3, with HSS in the backbones as repeating units (Fig. 2). P1 was synthesized by polyaddition of *E*-M1 or *Z*-M1 and hexamethylene diisocyanate (HDI) in the presence of di-*n*-butyltin dilaurate (DBTDL) in dimethylacetamide (DMAc), resulting in *E*-P1 and *Z*-P1 with relatively high number average molecular weights (*M*_n_s) and degrees of polymerization (DPs), which are comparable to those of other linear polymers with main-chain photoswitches.^45–49,51,52^ Two *E*-P1 were synthesized but used without distinction in the following experiments because of the similar *M*_n_, DP, and dispersity (*M*_w_/*M*_n_) values. *Z*-P1 is the first polymer having only the metastable isomer of a photoswitch as a repeating unit and was obtained due to the high thermal stability of *Z*-HSS. Polycondensation of *E*-M1 and adipoyl chloride for *E*-P2 successfully proceeded in dichloromethane (DCM) with triethylamine. In the absence of triethylamine, side reactions of HSS with HCl generated during the polymerization were observed with a number of unidentified peaks not originating from the *Z* isomer and indanones, which can be generated *via* oxidative cleavage of the C=C bond, in the ^1^H NMR spectrum of the resultant polymer (Fig. S11†). *E*-P3 was prepared by ADMET polymerization of *E*-M2 in DCM with a 2nd generation Hoveyda-Grubbs catalyst (HG-II) without side reactions involving the central C=C bond of HSS including *E*-to-*Z* isomerization, although SS was previously reported to undergo side reactions including *E*–*Z* isomerization with a 2nd generation Grubbs catalyst.^67^ The steric hindrance around the C=C bond of HSS would prevent coordination of the catalyst, as well as reactions with radicals during living radical polymerization in our previous study.^70^ From these results, both *E* and *Z* isomers of HSS were found to be available for various types of polymerization except under acidic conditions, promising a broad range of polymer applications. The low isolated yields of all polymers are attributed to incomplete polymerizations probably due to inexact feed ratios of the monomers in polyaddition and polycondensation and insufficient removal of the generated ethylene in ADMET polymerization. Macrocycles were considered to be hardly generated in the polymerizations as indicated by diffusion-ordered spectroscopy (DOSY) NMR spectra of the four polymers (Fig. S16, S18, S19, and S21†).^75^

### Photoisomerization in solution

**Fig. 3:**
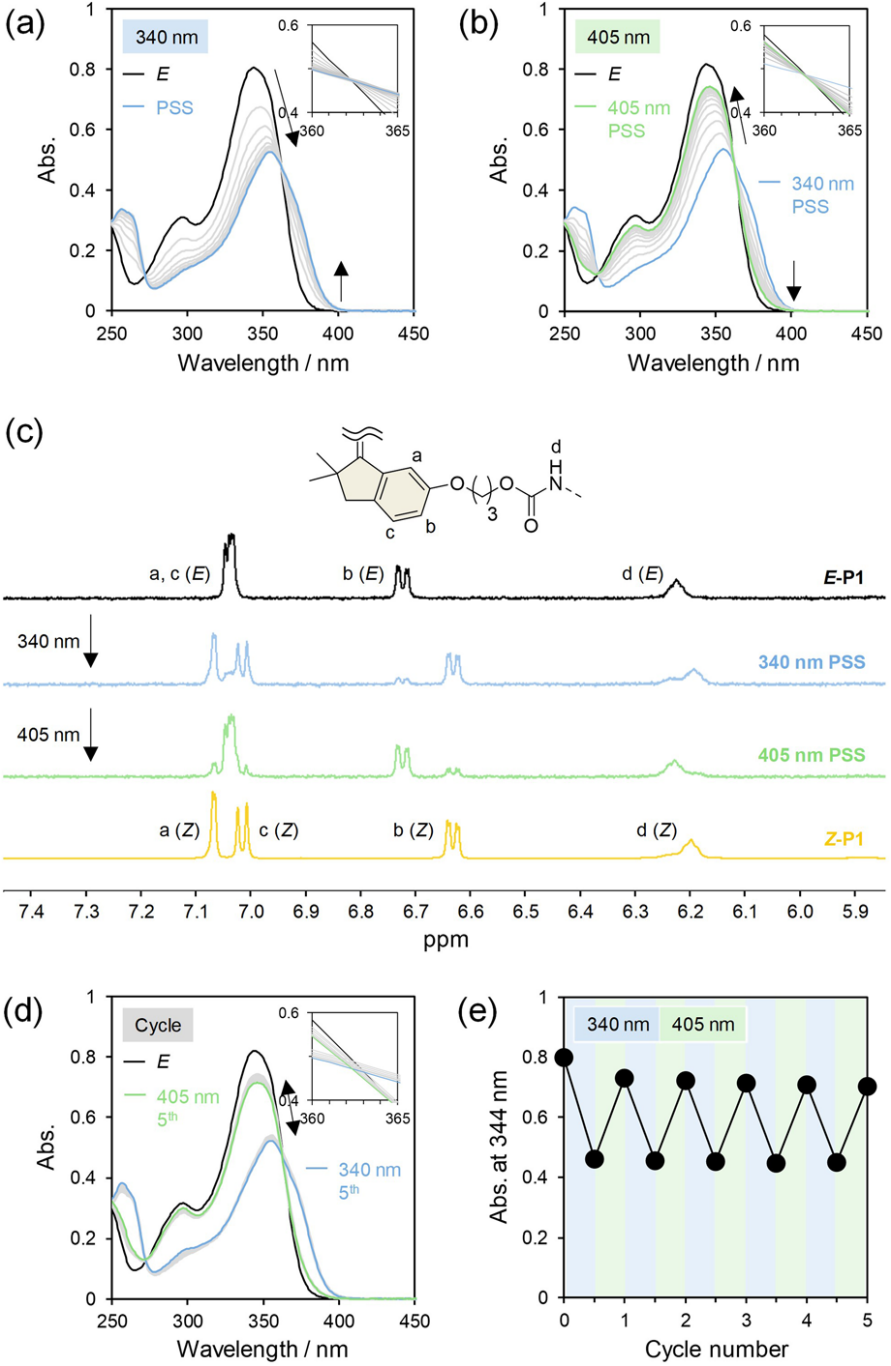
UV/vis absorption spectra of *E*-P1 upon (a) irradiation with 340 nm light and (b) subsequent irradiation with 405 nm light in THF (3.00 × 10^−5^ M). (c) ^1^H NMR spectra (500 MHz, THF-*d*_8_) of *E*-P1, *Z*-P1, and PSSs under 340 and 405 nm irradiation in THF-*d*_8_ (1.00 × 10^−4^ M). (d) UV/vis absorption spectra and (e) changes in absorbance at 344 nm upon alternating irradiation of *E*-P1 with 340 and 405 nm light in THF (3.00 × 10^−5^ M).

First, we examined photoisomerization of the main-chain HSS photoswitches in solution. The three polymers containing 100% *E* isomer, *E*-P1, *E*-P2, and *E*-P3, were individually dissolved in tetrahydrofuran (THF, 3.00 × 10^−5^ M) and irradiated with 340 nm UV light. In all three polymers, the UV/vis absorption spectra gradually approached those of the *Z* isomers^70,72^ and showed two isosbestic points at *ca.* 272 and 362 nm, indicating *E*-to-*Z* isomerization of the incorporated HSS photoswitches without noticeable side reactions (Fig. 3a, S13, and S14†). After 340 nm irradiation until photostationary states (PSSs), subsequent exposure of the solutions to 405 nm visible light caused the *Z*-to-*E* isomerization with the two isosbestic points (Fig. 3b, S13, and S14†). The *E*/*Z* ratios at the PSSs under 340 and 405 nm irradiation in deuterated THF (THF-*d*_8_, 1.00 × 10^−4^ M) were determined from the ^1^H NMR spectra to be 17/83 and 81/19 for *E*-P1, 16/84 and 82/18 for *E*-P2, and 19/81 and 81/19 for *E*-P3, respectively (Fig. 3c, S13, and S14†). In other words, the *E*-to-*Z* and *Z*-to-*E* photoisomerization reached more than 80% *Z* and *E* isomers, respectively, regardless of the linkage structure and are comparable to those of a small HSS derivative with two methoxy groups and a single HSS molecule incorporated in the center of a polymer chain in solution.^70,72^ Additionally, the reversible photoisomerization was observed repeatedly in at least five cycles by alternating irradiation with 340 and 405 nm light (Fig. 3d, e, S13, and S14†).

### Macromolecular conformational changes in solution

**Fig. 4:**
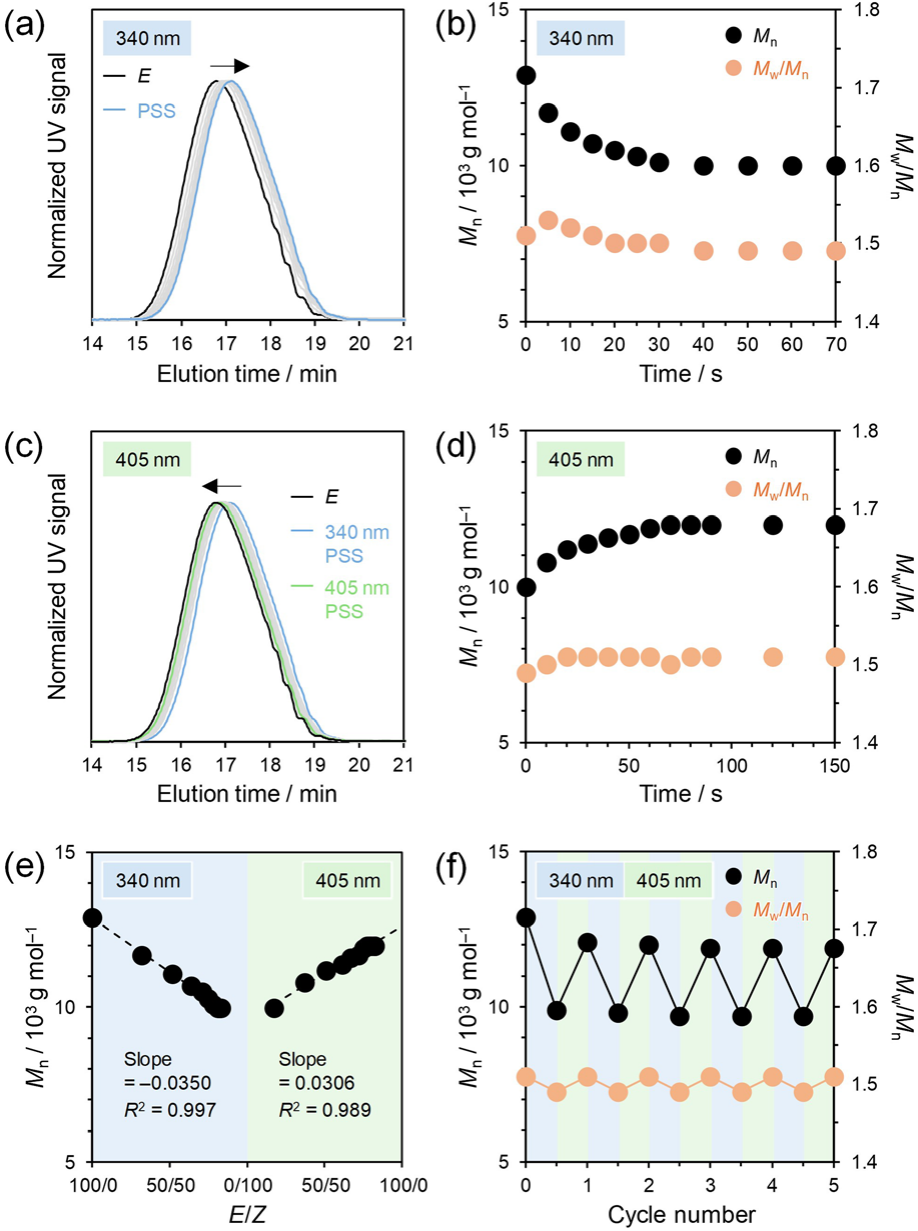
(a) SEC curves and (b) apparent *M*_n_s and *M*_w_/*M*_n_s of *E*-P1 during 340 nm irradiation in THF (3.00 × 10^−5^ M). (c) SEC curves and (d) apparent *M*_n_s and *M*_w_/*M*_n_s of 340 nm PSS during 405 nm irradiation in THF (3.00 × 10^−5^ M). (e) Apparent *M*_n_s at different *E*/*Z* ratios in the 340 nm and subsequent 405 nm irradiation of *E*-P1. (f) Changes in apparent *M*_n_s and *M*_w_/*M*_n_s upon alternating irradiation of *E*-P1 with 340 and 405 nm light in THF (3.00 × 10^−5^ M).

Next, we investigated conformational changes of the single polymer chains in solution induced by photoisomerization of the main-chain HSS photoswitches using size exclusion chromatography (SEC), which has been employed also in previous studies on main-chain photoswitches of AB, α-bisimine, and hydrazone.^20,21,24,45,46,52^ THF solutions of *E*-P1, *E*-P2, and *E*-P3 (3.00 × 10^−5^ M) were irradiated with 340 nm light and subsequently with 405 nm light under the same conditions as the above photoisomerization experiments, and apparent *M*_n_s and *M*_w_/*M*_n_s were calculated from the SEC curves at 40 °C. The SEC curve of *E*-P1 gradually shifted to a longer elution time upon 340 nm irradiation (Fig. 4a), corresponding to a continuous decrease in the apparent *M*_n_ (Fig. 4b). The *E*-to-*Z* isomerization is considered to increase the number of bends in the single polymer chain, shrink the polymer conformation, and reduce the hydrodynamic volume.^52^ Conversely, subsequent 405 nm irradiation of the 340 nm PSS solution shifted the SEC curve to a shorter elution time and increased the apparent *M*_n_ to almost the initial value (Fig. 4c and d), indicating that the *Z*-to-*E* isomerization restored the polymer conformation and hydrodynamic volume. Moreover, the SEC curves maintained the unimodality with almost constant *M*_w_/*M*_n_s throughout the photoirradiation (Fig. 4b and d), which excluded the possibility of side reactions such as chain scission. Therefore, we demonstrated that the polymer conformation reversibly switched between the swollen and shrank states by the main-chain HSS photoisomerization. Similar behavior was also observed in the *E*-P2 and *E*-P3 solutions (Fig. S24 and S25†). Additionally, the macromolecular conformational changes were supported by increases in the diffusion coefficients after the *E*-to-*Z* isomerization of *E*-P1, *E*-P2, and *E*-P3 in DOSY NMR measurements (Fig. S16–S22†).

To gain a more detailed understanding of the macromolecular conformational changes, we analyzed the correlation between the apparent *M*_n_s and the *E*/*Z* ratios determined from the UV/vis absorption and ^1^H NMR spectra (Fig. S15†). The apparent *M*_n_s of the three polymers decreased and increased in proportion to the *Z* and *E* ratios, respectively (Fig. 4e, S24, and S25†). The absolute values of the slopes increased in the order of P2 (*ca.* 0.02) < P1 (*ca.* 0.03) < P3 (*ca.* 0.04) and corresponded to the DPs, 15 (P2), 21 (P1), and 25 (P3) (Fig. 2), although probably affected by other factors as well, including the initial *M*_n_s, linker lengths between HSS molecules (or linear densities of HSS in the single polymer chains), chain rigidity, and interactions with the solvent. A larger number of HSS incorporated in the backbones was demonstrated to allow more significant conformational changes of the single polymer chains. We also calculated rates of the apparent *M*_n_ changes (Δ*M*_n_ = (*M*_n,PSS_ − *M*_n,before irradiation_)/*M*_n,initial_ × 100%) to quantify the conformational variations. Upon 340 nm irradiation for *E*-to-*Z* isomerization until PSSs, the apparent *M*_n_s of *E*-P1, *E*-P2, and *E*-P3 decreased by 22%, 23%, and 29%, respectively, which are comparable to or greater than those induced by other main-chain photoswitches of AB, α-bisimine, and hydrazone.^20,21,24,45,46,52^ Upon subsequent 405 nm irradiation for *Z*-to-*E* isomerization until PSSs, the apparent *M*_n_s of P1, P2, and P3 increased (recovered) by up to 16%, 16%, and 22%, respectively. P3 with the highest DP and the shortest linker length between HSS molecules (the highest linear density of HSS in the single polymer chain) showed the largest Δ*M*_n_s among the three polymers, while the photoisomerization yields in addition to the above other factors would also dictate the Δ*M*_n_s. Furthermore, the reversible photoinduced conformational changes were repeatedly observed over at least five cycles (Fig. 4f and S23–S25) as well as the photoisomerization (Fig. 3d and e). From these results, we concluded that the main-chain HSS photoswitches are capable of controlling the polymer conformations in solution in a reversible and precise manner based on the *E*/*Z* ratio, unlike the previous main-chain photoswitches of AB, α-bisimine, thioindigo, and spiropyran without thermal stability.^20,21,24,45,46,50,51^ As for the main-chain hydrazone photoswitches with high thermal stability, the polymer conformations in solution were not sufficiently restored despite almost complete recovery of the *E*/*Z* ratio.^52^ This is probably due to the flexible molecular structures originating from the single bond free rotation, although the reversible control has not yet been explored in detail. The high thermal stability and rigid molecular structure of HSS in addition to the large structural changes would enable the precise photocontrol of conformations of single polymer chains.

### Hydrogen bonding in P1

**Fig. 5:**
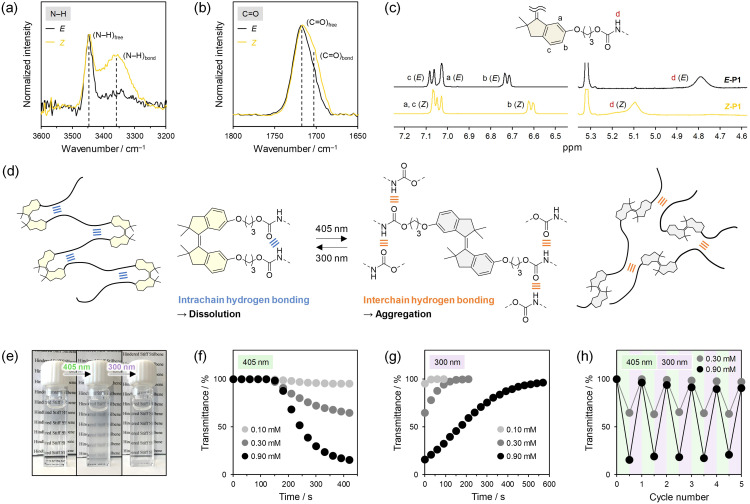
FTIR spectra of *E*-P1 and *Z*-P1 in DCM (100 mg mL^−1^) focused on (a) N–H and (b) C=O stretching vibrations. (c) ^1^H NMR spectra (500 MHz) of *E*-P1 and *Z*-P1 in CD_2_Cl_2_. (d) Illustration of intrachain and interchain hydrogen bonding in *Z*-P1 and *E*-P1, respectively. (e) Pictures of photoreversible precipitation and dissolution of *Z*-P1 in toluene (0.90 mM) by 405 nm irradiation for 7 min and subsequent 300 nm irradiation for 9.5 min. Transmittance changes of toluene solutions of *Z*-P1 at 0.10, 0.30, and 0.90 mM during (f) 405 nm irradiation and (g) subsequent 300 nm irradiation. (h) Transmittance changes of toluene solutions of *Z*-P1 at 0.30 and 0.90 mM upon alternating irradiation with 405 and 300 nm light.

More interestingly, *E*-P1 and *Z*-P1 showed completely different solubilities in toluene. Specifically, *Z*-P1 is soluble but *E*-P1 is insoluble, although both polymers are soluble in other solvents such as DCM, chloroform, and THF. *E*-P2, *E*-P3, and their *Z*-rich (*ca.* 80%) mixtures obtained by irradiation with 300 nm UV light, which also yielded more than 80% *Z* ratios at the PSSs as well as 340 nm UV light (Fig. S37–S39†), were also soluble in all the solvents including toluene. We attributed the unique insolubility of *E*-P1 in toluene to interchain hydrogen bonding between the urethane linkages and measured FTIR and ^1^H NMR spectra of *E*-P1 and *Z*-P1 in these solvents. A sharp peak corresponding to the stretching vibration of free N–H bonds was observed at *ca.* 3450 cm^−1^ in the FTIR spectrum of *E*-P1 in DCM (100 mg mL^−1^) (Fig. 5a). On the other hand, another broad peak appeared around 3350 cm^−1^ in the spectrum of *Z*-P1 in DCM (100 mg mL^−1^) and originated from hydrogen-bonded N–H groups.^76,77^ Similarly, the stretching vibration of free C=O bonds was predominantly observed in *E*-P1 as a broad peak around 1720 cm^−1^, a part of which was shifted to *ca.* 1700 cm^−1^ in *Z*-P1 and ascribed to hydrogen-bonded C=O groups (Fig. 5b).^76^ Therefore, we concluded that in DCM, the urethane linkages of *Z*-P1 form intrachain hydrogen bonding but those of *E*-P1 hardly form any hydrogen bonding. In the ^1^H NMR spectrum of *Z*-P1 in CD_2_Cl_2_, the N–H signal shifted downfield compared with that observed in *E*-P1,^76^ supporting the intrachain hydrogen bonding in *Z*-P1 (Fig. 5c). Similar experiments in chloroform and THF also led to the same conclusion in a complementary way (Fig. S26 and S27†). In chloroform, a broad peak and a downfield signal derived from hydrogen-bonded N–H groups were observed only in the FTIR and ^1^H NMR spectra of *Z*-P1, respectively, while no differences were found in the C=O stretching vibration of both polymers probably because the C=O groups formed hydrogen bonding with chloroform. Conversely, in THF, only *Z*-P1 showed a broad peak originating from hydrogen-bonded C=O groups in the FTIR spectrum, but both polymers exhibited similar peaks and signals of N–H groups hydrogen-bonded with THF in the FTIR and ^1^H NMR spectra, respectively. In addition to these results, the FTIR spectrum of the *E*-P1 powder, in which broad peaks derived from hydrogen-bonded N–H and C=O groups were observed (Fig. S28†), demonstrated that *E*-P1 formed interchain hydrogen bonding between the urethane linkages in toluene and aggregated (Fig. 5d). This conclusion was also supported by the FTIR spectra of *Z*-P1 in toluene under 405 nm irradiation, in which soluble and insoluble components were observed and the intensity of the peaks originating from the hydrogen-bonded N–H and C=O groups increased (Fig. S30†), and the ^1^H NMR spectra of *Z*-P1 in toluene-*d*_8_ under 405 nm irradiation, in which only soluble components were observed but the hydrogen-bonded N–H signal was gradually shifted upfield to be free (Fig. S30†).

The intrachain and interchain hydrogen bonding between the urethane linkages of P1 in toluene was controllable by the HSS photoisomerization, and these nanoscopic changes were amplified to macroscopic photoswitching of the dissolved and precipitated states (Fig. 5e and Video S1–S3†). We prepared toluene solutions of *Z*-P1 at different concentrations, 0.10, 0.30, and 0.90 mM, irradiated the solutions with 405 nm light without stirring to induce *Z*-to-*E* isomerization, and monitored the transmittance at 600 nm. The transmittance gradually decreased from 100% after an induction period of about 150 s at all the concentrations (Fig. 5f). The induction periods would indicate that the *Z*-to-*E* photoisomerization was the rate-limiting step of the precipitation and once the single polymer chain reached a certain *E*/*Z* ratio, the aggregation began. A lower concentration of *Z*-P1 led to a higher isomerization rate, while a higher concentration led to earlier aggregation at a smaller *E*/*Z* ratio, which was indicated by an increase in the baseline (light scattering) (Fig. S31†). Consequently, the induction periods at all the concentrations were similar. A higher concentration also resulted in a more significant reduction in the transmittance. Subsequently, the opaque solutions were irradiated with 300 nm light to undergo *E*-to-*Z* isomerization. The transmittance was gradually restored to 100%, *i.e.*, complete dissolution (Fig. 5g). A higher concentration of P1 required a longer period of irradiation for the transmittance recovery, probably because the HSS photoisomerization and switching of the hydrogen bonding occurred at the aggregate surfaces and additionally, the irradiating light was scattered by the aggregates. The precipitation and dissolution could be locally induced using spot light sources (Video S1–S3†) and repeatedly switched over at least five cycles by alternating irradiation with 405 and 300 nm light (Fig. 5h and S32†). Photocontrol of hydrogen bonding has been demonstrated predominantly in small molecules in the context of supramolecular chemistry by using structural changes of molecular switches and motors such as AB, SS, and overcrowded alkenes.^55,64,78–81^ However, that is extremely rare in polymers and, to the best of our knowledge, limited to the side chains.^79,82^ Therefore, photocontrol of main-chain hydrogen bonding was demonstrated for the first time in this study. Moreover, photoswitching of precipitation and dissolution of polymers has mainly employed polarity changes of photoswitches in the side chains to shift their lower critical solution temperatures (LCSTs) but been available only in a narrow temperature range, *i.e.*, the shift range.^83–85^ In contrast, photoswitching of precipitation and dissolution of P1 was based on the new mechanism, *i.e.*, switching between intrachain and interchain hydrogen bonding, and workable in a wide temperature range because *Z*-P1 basically dissolved in toluene at all temperatures, although the *E*-rich (*ca.* 80%) P1 also dissolved in toluene at high temperatures probably due to dissociation of the interchain hydrogen bonding (Fig. S33†).

### Thermal properties in bulk

Then, we examined thermal properties of the polymers in the bulk state. In the thermogravimetric analysis (TGA) curves of *E*-P1, *Z*-P1, *E*-P2, and *E*-P3, no decomposition was observed below 300 °C, indicating similar and high thermal stability regardless of the isomer and backbone (Fig. S34 and Table S2†). *E*-P1, *Z*-P1, *E*-P2, and *E*-P3 showed *T*_g_s at 73, 72, 44, and 64 °C, respectively, without melting peaks in the differential scanning calorimetry (DSC) curves (Fig. S35 and Table S2†) and showed only a broad peak in the X-ray diffraction (XRD) profiles (Fig. S36†). These results indicate that all four polymers are amorphous glasses at RT. Surprisingly, *E*-P1 and *Z*-P1 composed of the same polyurethane backbone but a different isomer had almost the same *T*_g_s. The isomer effect of main-chain photoswitches on *T*_g_s has been rarely examined including in AB^23,36–38^ and was also small in the case of hydrazone (Δ*T*_g_s < 10 °C).^52^ To further investigate the isomer effect, we prepared P1, P2, and P3 with two different *E*/*Z* ratios by 300 nm exposure of each 100% *E* polymer in THF (1.00 × 10^−3^ M) and obtained their DSC curves. The *T*_g_s were almost independent of the *E*/*Z* ratio in all three polymers (Fig. S40†). Therefore, the large conformational changes of the single polymer chains by isomerization of the main-chain HSS photoswitches found in solution hardly affected the segmental mobility in bulk. The slight difference in polarity between the *E* and *Z* isomers of HSS (Fig. S41†) and resulting small variations in interchain interactions would contribute to the unchanged segmental mobility.

Thermal stability of the main-chain HSS photoswitches in the bulk state was also evaluated. When *E*-P1, *Z*-P1, *E*-P2, and *E*-P3 were heated at 80 °C above the *T*_g_s under vacuum for 2 days, no thermal isomerization was observed in the ^1^H NMR spectra (Fig. S42†). However, only the SEC curve of *E*-P3 was broadened with emergence of higher-molecular-weight components (Fig. S43 and Table S3†) probably due to cross-linking between the C=C bonds of the backbone except HSS, which might be related to a tiny and broad endothermic peak above 100 °C in the DSC curve (Fig. S35†). Then, we monitored thermal isomerization of the polymers except *E*-P3 at 100, 120, and 140 °C. The main-chain HSS photoswitches thermally isomerized at these temperatures and reached equilibria containing about 70% *E* isomer (Fig. S47, S51, and S55†) similar to the small HSS derivative with two methoxy groups in solution.^72^ The *t*_1/2_s of *Z*-to-*E* thermal isomerization in the bulk *Z*-P1 were approximately 7 days at 100 °C, 15 h at 120 °C, and 4 h at 140 °C (Fig. S51†) and longer than those of the small HSS derivative in solution, about 7 h at 120 °C and 1 h at 140 °C.^72^ When the three polymers were heated at 100 °C for more than 1 week, insoluble components in CDCl_3_ appeared with tiny unidentified peaks in the ^1^H NMR spectra (Fig. S44, S48, and S52†) presumably because of side reactions including cross-linking.

### Photoisomerization in the glassy state

**Fig. 6:**
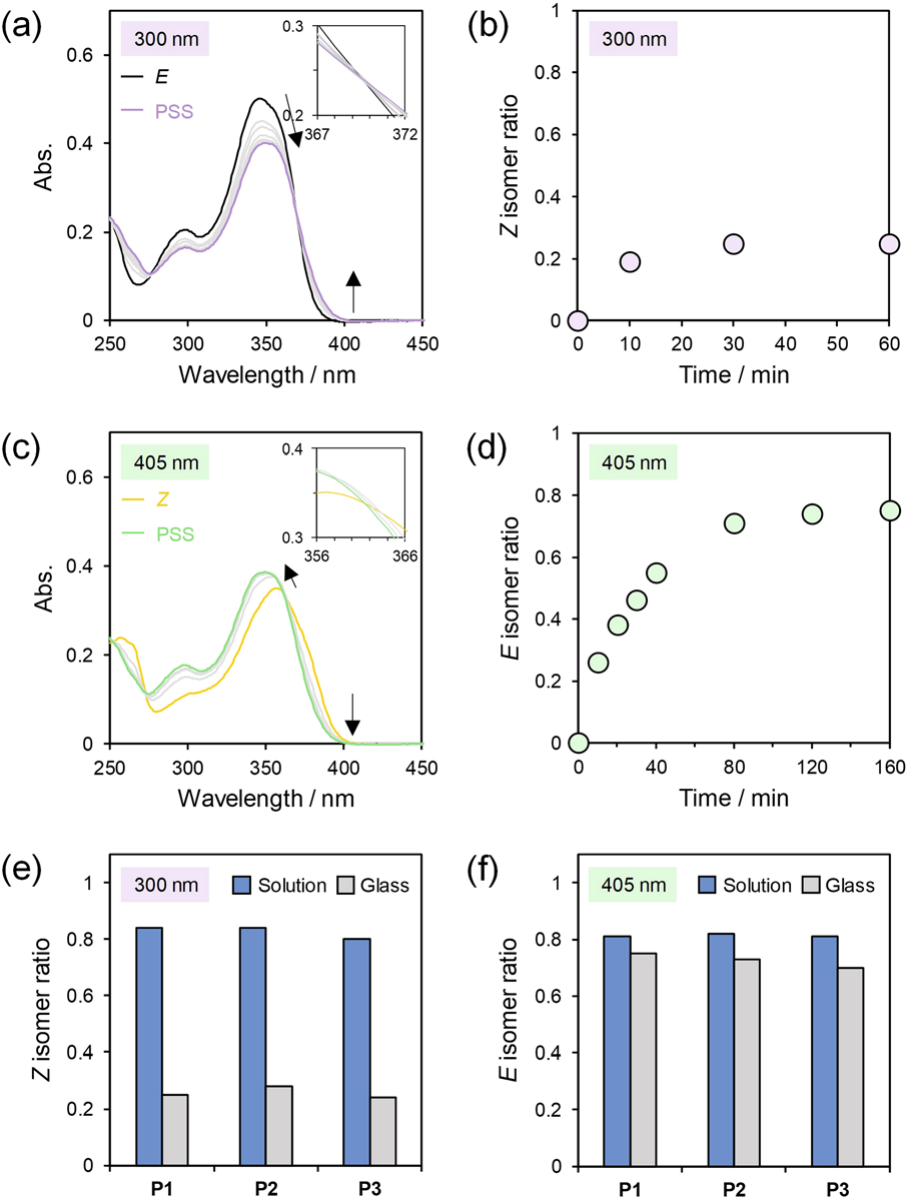
UV/vis absorption spectra of (a) *E*-P1 thin film upon 300 nm irradiation and (c) *Z*-P1 thin film upon 405 nm irradiation. *Z* and *E* isomer ratios determined from ^1^H NMR spectra in the photoisomerization of (b) *E*-P1 and (d) *Z*-P1 thin films. Summary of *Z* and *E* isomer ratios determined from ^1^H NMR spectra at (e) 300 nm and (f) 405 nm PSSs in THF solution and in the glassy state of P1, P2, and P3.

Photoisomerization of the main-chain HSS photoswitches with restricted molecular mobility in the glassy state was investigated by using thin films of the polymers with 70–180 nm thickness (Table S4†). The thin films were prepared by spin coating of *E*-P1, *Z*-P1, *E*-P2, and *E*-P3 on quartz substrates whose surfaces were modified with a hydrophobic monolayer using 1,1,1,3,3,3-hexamethyldisilazane (HMDS). When the thin films of *E*-P1, *E*-P2, and *E*-P3 were exposed to 300 nm light, *E*-to-*Z* isomerization and PSSs were observed in the UV/vis absorption spectra (Fig. 6a, S56, and S57†) similar to the solutions (Fig. 3a, S13, and S14†). However, the *Z* ratios at the PSSs determined from the ^1^H NMR spectra of the thin films dissolved in CDCl_3_ were 25%, 28%, and 24% in P1, P2, and P3, respectively (Fig. 6b and S56–S58†), and significantly lower than those (>80%) in solution (Fig. S37–S39†). The ratio at the PSS was unchanged in a similar experiment of a thinner *E*-P1 film prepared using a solution diluted to half the concentration, ensuring that the UV light penetrated deep into the whole films (Fig. S59†). Therefore, the *E*-to-*Z* photoisomerization in the glassy state was much harder than that in solution. These results are similar to photoisomerization of main-chain diarylethene and α-bisimine photoswitches in the thin films, the conversions of which were up to *ca.* 30% even above the *T*_g_s.^45,48^ In contrast, the *Z*-to-*E* photoisomerization proceeded well when the thin film of *Z*-P1 was irradiated with 405 nm light. Surprisingly, the *E* ratio determined from the ^1^H NMR spectra reached 75% at the PSS comparable to that (81%) in solution (Fig. 6d and S60†), although the slight changes in the UV/vis absorption spectra seemed to indicate a low yield (Fig. 6c). The restricted molecular mobility in the glassy state might distort the spectra. Similarly, *Z*-rich (>80%) thin films of P2 and P3 prepared by photoisomerization of the *E* polymers in solution and subsequent spin coating attained PSSs containing 73% and 70% *E* isomer, respectively, under 405 nm irradiation (Fig. S61 and S62†). The *E*/*Z* ratios at the PSSs in the both directions of photoisomerization of P1, P2, and P3 in solution and in the glassy state are summarized in Fig. 6e and f. The results clearly indicate that only the *E*-to-*Z* photoisomerization was inhibited in the glassy state. The contracting *E*-to-*Z* direction seems unfavorable in the glassy state, similar to the photocyclization reaction from the open-ring form to the closed-ring form of main-chain diarylethene photoswitches.^45^ Nevertheless, the photoisomerization yields of the main-chain HSS photoswitches are superior to those of previous main-chain photoswitches in the glassy (or solid) state^45,48^ and even small molecular photoswitches dispersed in glassy polymers,^86^ although their reverse photoisomerization has not been evaluated in a quantitative manner except in one paper on diarylethene.^45^

Reversible *E*-to-*Z*-to-*E* photoisomerization was not observed in the *E*-P1 thin film (Fig. S63†); the *E*/*Z* ratio was changed from 100/0 to 25/75 under 300 nm irradiation but was almost unchanged under subsequent 405 nm irradiation. On the other hand, interestingly, reversible *Z*-to-*E*-to-*Z* photoisomerization was partially observed in the *Z*-P1 thin film (Fig. S66†); the *E*/*Z* ratio was changed from 0/100 to 75/25 under 405 nm irradiation and changed to 56/44 under subsequent 300 nm irradiation. These results might indicate that the *E* isomer generated in the *Z*-P1 thin film had different conformations from those in the *E*-P1 thin film, *e.g.*, intermediate conformations that easily undergo the contracting *E*-to-*Z* isomerization, as similar behavior was found in main-chain diarylethene photoswitches.^45^ The distorted UV/vis absorption spectra possibly represent the conformations (Fig. 6c). Only the reversible *Z*-to-*E*-to-*Z* photoisomerization was also observed in the P2 and P3 thin films (Fig. S64, S65, S67, and S68†).

### Wettability of thin film surfaces

**Fig. 7:**
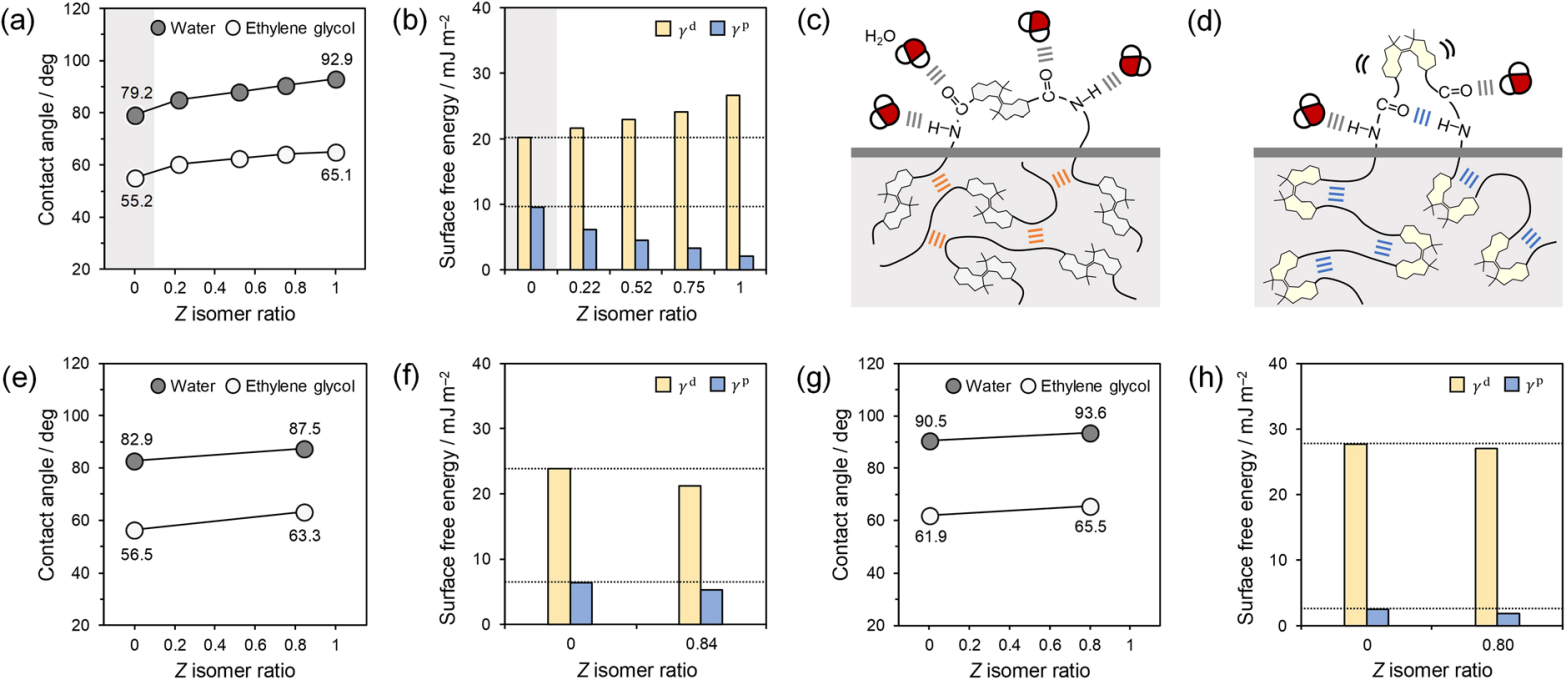
(a) Static contact angles of water and ethylene glycol on P1 thin films with different *E*/*Z* ratios (*n* = 4), and (b) *γ*^d^ and *γ*^p^ of the surface free energies. The 0% *Z* isomer highlighted in light gray is *E*-P1. The others are prepared by photoirradiation of *Z*-P1. Illustration of interchain (orange) and intrachain (blue) hydrogen bonding between urethane linkages and hydrogen bonding between urethane linkage and water (gray) in (c) *E*-P1 and (d) *Z*-P1 thin films. Static contact angles of water and ethylene glycol on (e) P2 and (g) P3 thin films with different *E*/*Z* ratios (*n* = 4), and *γ*^d^ and *γ*^p^ of surface free energies of (f) P2 and (h) P3 thin films. Small standard deviations are added to (a), (e), and (g) as error bars but buried.

Finally, we examined the effects of nanoscopic macromolecular conformational changes on macroscopic wettability of thin film surfaces. Thin films of P1 with five different *E*/*Z* ratios and P2 and P3 with two different *E*/*Z* ratios were prepared by spin coating on HMDS-treated quartz substrates. The thin film surfaces were observed by atomic force microscopy (AFM) to be smooth with a root mean square (RMS) roughness of less than 1.2 nm (Table S5†). Static contact angles of water and ethylene glycol on the thin film surfaces of P1, P2, and P3 increased with increasing the *Z* ratio (Fig. 7a, e, g and Table S6†), which indicates enhanced hydrophobicity but was opposite to a prediction based on the polarity of the isomers (non-polar *E* and slightly polar *Z*, Fig. S41†), as reported in AB and spiropyran.^87,88^ To gain further insight into the unexpected results, the surface free energies *γ* (= *γ*^d^ + *γ*^p^) were calculated from the contact angles.^89^ In the P1 thin films, the increase in the *Z* ratio increased the dispersion component *γ*^d^ and decreased the polar component *γ*^p^ (Fig. 7b). Additionally, hydrogen bonding between the urethane linkages was confirmed in the *E*-P1 and *Z*-P1 thin films by FTIR measurements (Fig. S70†). Based on these results and the discussion regarding the solutions (Fig. 5), it is considered that the urethane linkages of the *E* isomers form interchain hydrogen bonding (Fig. 7c), which restricts the molecular mobility and gives a low *γ*^d^ value, while those of the *Z* isomers form intrachain hydrogen bonding (Fig. 7d), which enhances the molecular mobility and gives a high *γ*^d^ value, inside the thin films. At the thin film surfaces, the urethane linkages of the *E* isomers would be partially free and form hydrogen bonding with the probe liquids (Fig. 7c), which gives a high *γ*^p^ value, while those of the *Z* isomers would preferentially form intrachain hydrogen bonding rather than with the probe liquids due to the proximity effect (Fig. 7d), which gives a low *γ*^p^ value. Therefore, the increased *γ*^d^ and decreased *γ*^p^ with increasing the *Z* ratio are attributed to the enhanced molecular mobility and the reduced interactions with the probe liquids, respectively, by the intrachain hydrogen bonding. The clear reduction in *γ*^p^ could be observed due to the small polarity difference between the *E* and *Z* isomers. The reason for the unexpected hydrophobization on the P1 thin film surface with increasing the *Z* ratio (Fig. 7a) is probably because the contribution of *γ*^p^ exceeded that of *γ*^d^. Additionally, the hysteresis of water contact angles increased with increasing the *Z* ratio (Fig. S71 and Table S7†) and also indicates the enhanced molecular mobility of hydrophilic parts.^90^ Similar trends of the static contact angles and *γ*^p^ values were also observed when the *Z*-P1 thin film was irradiated with 405 nm light and measured *in situ* (Fig. S72 and Table S8†). On the other hand, both *γ*^d^ and *γ*^p^ of P2 and P3, which are incapable of forming hydrogen bonding independently, decreased as the *Z* ratio increased (Fig. 7f and h). The decreased *γ*^d^ would originate from restricted molecular mobility by contraction of the macromolecular conformations as found in the solutions, which can increase the hysteresis of water contact angles (Fig. S71 and Table S7†).^91^ The decreased *γ*^p^ would originate from reduced interactions between the probe liquids and hydrophilic parts such as the ester linkages in P2 and ether linkages in P3 by steric hindrance of the *Z* isomers. From these results, we concluded that the macroscopic wettability was governed by the nanoscopic structures and properties, *i.e.*, hydrogen bonding modes in P1 and macromolecular conformations and steric hindrance of HSS in P2 and P3. The wettability was controlled for the first time based on photoswitching between interchain and intrachain hydrogen bonding, due to the independence of the HSS polarity, (bulk) *T*_g_s, and surface morphologies from the *E*/*Z* ratio.

## Conclusions

In this study, we incorporated thermally bistable HSS photoswitches into the main chains of three types of linear polymers with different chemical linkages, polyurethane, polyester, and polyene, and demonstrated the photoisomerization, macromolecular conformational changes, and macroscopic reflection in solution, in bulk, and at thin film surfaces. The polymer conformations in solution, *i.e.*, hydrodynamic volume, were correlated well with the *E*/*Z* ratio and reversibly and precisely controlled by photoirradiation. Particularly in the polyurethanes, the conformational transformations accompanied the changes between interchain and intrachain hydrogen bonding, enabling photoswitching of the solution transmittance and the surface wettability. Furthermore, the *Z*-to-*E* photoisomerization yields in the glassy state exceeded 70%, which were comparable to those in solution and extraordinarily high despite the restricted molecular mobility. This study is the first to quantitatively examine both directions of photoisomerization of main-chain photoswitches in the bulk state (in thin films) and amplify the nanoscopic macromolecular conformational changes to the macroscopic phenomena. Therefore, we believe that the findings of this study foster the development of smart polymer systems based on photoswitches and pave the way for their practical and unconventional applications.

## Data availability

The data supporting this article have been included in the ESI.†

## Author contributions

K. I. and Y. O. designed and directed the project. N. K. mainly performed all the experiments. A. S. contributed to the synthesis of the monomers and polymers. R. T. contributed to the DOSY NMR measurements. T. M. contributed to the experimental design and discussion of the wettability of the polymer thin film surfaces. N. K., K. I., and Y. O. wrote the manuscript. All authors discussed the results and contributed to the manuscript editing.

## Conflicts of interest

There are no conflicts to declare.

## Supplementary Material

SC-015-D4SC06470D-s001

SC-015-D4SC06470D-s002

SC-015-D4SC06470D-s003

SC-015-D4SC06470D-s004
