# Supplementary material for: Main-chain stiff-stilbene photoswitches in solution, in bulk, and at surfaces
Source: Chem Sci. 2024 Nov 19;15(48):20545–55. doi: 10.1039/d4sc06470d (PMC11587148; doi:10.1039/d4sc06470d)
Supplement: SC-015-D4SC06470D-s001 [file SC-015-D4SC06470D-s001.pdf]

Supplementary Information (SI) for Chemical Science.
This journal is © The Royal Society of Chemistry 2024

Electronic Supplementary Information

Main-chain stiff-stilbene photoswitches in solution, in bulk, and at surfaces

Naoki Kaneda, Keiichi Imato,* Ayane Sasaki, Ryo Tanaka, Ichiro Imae, Toyoaki Hirata, Takuya Matsumoto and Yousuke Ooyama*

Applied Chemistry Program, Graduate School of Advanced Science and Engineering, Hiroshima University, 1-4-1 Kagamiyama, Higashihiroshima, 739-8527, Japan.

E-mail: kimato@hiroshima-u.ac.jp, yooyama@hiroshima-u.ac.jp

Table of contents

Materials and measurements S3

Synthesis of ***E*-P1** S5

Synthesis of ***Z*-P1** S7

Synthesis of ***E*-P2** S9

Synthesis of ***E*-P3** S11

Side reactions in polycondensation without triethylamine for ***E*-P2** S15

Photoisomerization in solution S16

DOSY NMR measurements S20

Macromolecular conformational changes in solution S24

Hydrogen bonding in **P1** S27

Thermal properties in bulk S34

Photoisomerization in the glassy state S50

Wettability of thin film surfaces S58

References S63

Materials

All solvents and reagents were purchased from FUJIFILM Wako Pure Chemical, Tokyo Chemical Industry, Sigma Aldrich, or Nacalai Tesque and used as received unless otherwise noted. Milli-Q water (resistivity >18 MΩ cm) was prepared using a Merck Direct-Q UV 3 water purification system. Hexamethylene diisocyanate (HDI) and adipoyl chloride were purified by distillation. The *E* and *Z* isomers of a sterically hindered stiff stilbene (HSS) derivative with two hydroxy groups, ***E*-M1** and ***Z*-M1**, were synthesized and isolated as monomers according to our previously published methods.[1] The *E* isomer of an HSS derivative with two hydroxy groups at the C6 and C6' positions, ***E*-HSS-diol**, was also obtained according to our previously published methods.[1]

Measurements

^{1}H NMR and ^{13}C NMR spectra were recorded at 25 °C using Varian-400 (400 MHz) and Varian-500 (500 MHz) FT NMR spectrometers in $CDCl_3$, CD_2Cl_2, acetone-d_6, tetrahydrofuran (THF)-d_8, or toluene-d_8. Diffusion-ordered spectroscopy (DOSY) NMR spectra were recorded at 27 °C on Varian-500 (500 MHz) FT NMR spectrometer in CD_2Cl_2 using the bipolar pulse pair-stimulated echo (BPPStE) sequence with a diffusion delay (Δ) of 50 ms, length of the gradient pulse (δ) of 2 ms, and gradient field strengths in the range of 0.02–0.6 T/m with exponential lamp steps. Number of scans for each gradient field strength was 16. To prevent the convection problem, the measurements were performed using a 3 mm tube, and polymers were dissolved in CD_2Cl_2 and stirred at RT in the dark at least overnight, prior to DOSY NMR measurements. DOSY spectra were processed in the MestReNova 11.0.4 software using Basyian DOSY Transform with a resolution factor of 0.5, two repetitions, and 64 points in the diffusion dimension over the range from 1×10^{-11} to 1×10^{-7} m^2 s^{-1}. High-resolution mass spectral (HRMS) data were acquired using a Thermo Fisher Scientific LTQ Orbitrap XL. Photoabsorption spectra were obtained at room temperature (RT) using a Shimadzu UV-3600i Plus spectrophotometer. Size exclusion chromatography (SEC) measurements were performed at 40 °C using a Shimadzu Prominence-i LC-2030 plus with a guard column (LF-G, Shodex), two series-connected columns (LF-804, Shodex), a UV detector, and a differential refractive index (RI) detector (RID-20A). THF was used as the eluent, and

polystyrene standards were used to calibrate the SEC system. Polymers were dissolved in THF and stirred at RT in the dark at least overnight, prior to SEC measurements. Flourier-transform infrared (FTIR) spectra were recorded at RT using a Shimadzu IRTracer-100 with a Quest single-reflection ATR accessory. Transmittance measurements were conducted at RT or a heating rate of 5 °C min^{-1} under nitrogen atmosphere using a Shimadzu UV-3600i Plus spectrophotometer. Thermogravimetric analyses (TGA) were conducted at a heating rate of 10 °C min^{-1} under nitrogen atmosphere using a Rigaku Thermo plus EV02 TG-DTA8122. Differential scanning calorimetry (DSC) measurements were performed at a heating rate of 10 °C min^{-1} under nitrogen atmosphere using a Hitachi DSC7000X. X-ray diffraction (XRD) patterns were obtained at RT using a Rigaku MiniFlex600-C/CM diffractometer with a Cu Kα radiator. Thickness of thin films was measured using a Keyence VK-9700 3D laser microscope. Static and dynamic contact angles were measured at RT by the sessile drop and extension/contraction methods using a Kyowa Interface Science DMo-602 contact angle meter. Surface roughness values were obtained by atomic force microscopy (AFM) measurements in dynamic mode using a Shimadzu SPM-9700HT.

Synthesis of *E*-P1

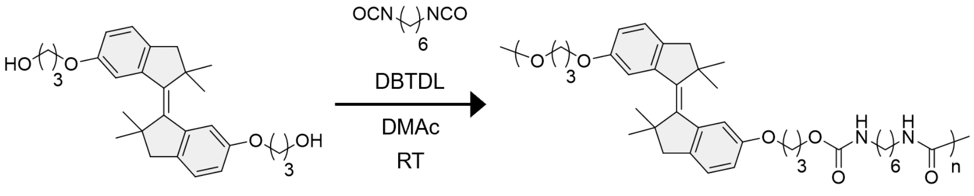

Scheme S1 Synthetic route for ***E*-P1**.

***E*-P1.** A solution of ***E*-M1** (501 mg, 1.15 mmol) and di-*n*-dibutyltin dilaurate (DBTDL, 3.40 µL, 5.65 µmol) in dehydrated dimethylacetamide (DMAc, 3.1 mL) was prepared under nitrogen atmosphere. HDI (183 µL, 1.14 mmol) was then added dropwise to the solution under nitrogen atmosphere. After stirring at RT for 30 h, methanol (0.5 mL) was added to the reaction mixture and stirred for 3 h. The mixture was precipitated into water, and the precipitate was collected by filtration, dissolved in dichloromethane (DCM, 3 mL), and reprecipitated into a mixed solvent of hexane (36 mL) and isopropanol (4 mL). The precipitate was collected by filtration and dried under vacuum to give ***E*-P1** as a white solid (501 mg, 72% yield). ^{1}H NMR (500 MHz, $CDCl_3$, Fig. S1): δ (ppm) = 7.08–7.02 (m, 4H, aromatic), 6.76–6.69 (m, 2H, aromatic), 4.70 (s, 2H, NH), 4.25 (t, J = 5.9 Hz, 4H, OCH_2), 4.03 (t, J = 6.0 Hz, 4H, OCH_2), 3.17–3.08 (m, 4H, $NHCH_2$), 2.70 (s, 4H, CH_2), 2.15–2.02 (m, 4H, CH_2), 1.51–1.40 (m, 4H, CH_2), 1.35–1.25 (m, 16H, CH_2 and CH_3). The number average molecular weights (M_ns) and polydispersity indices (M_w/M_ns) were determined by SEC (Fig. S2). n1: M_n = 12900 g mol^{-1}, M_w/M_n = 1.51. n2: M_n = 13200 g mol^{-1}, M_w/M_n = 1.61.

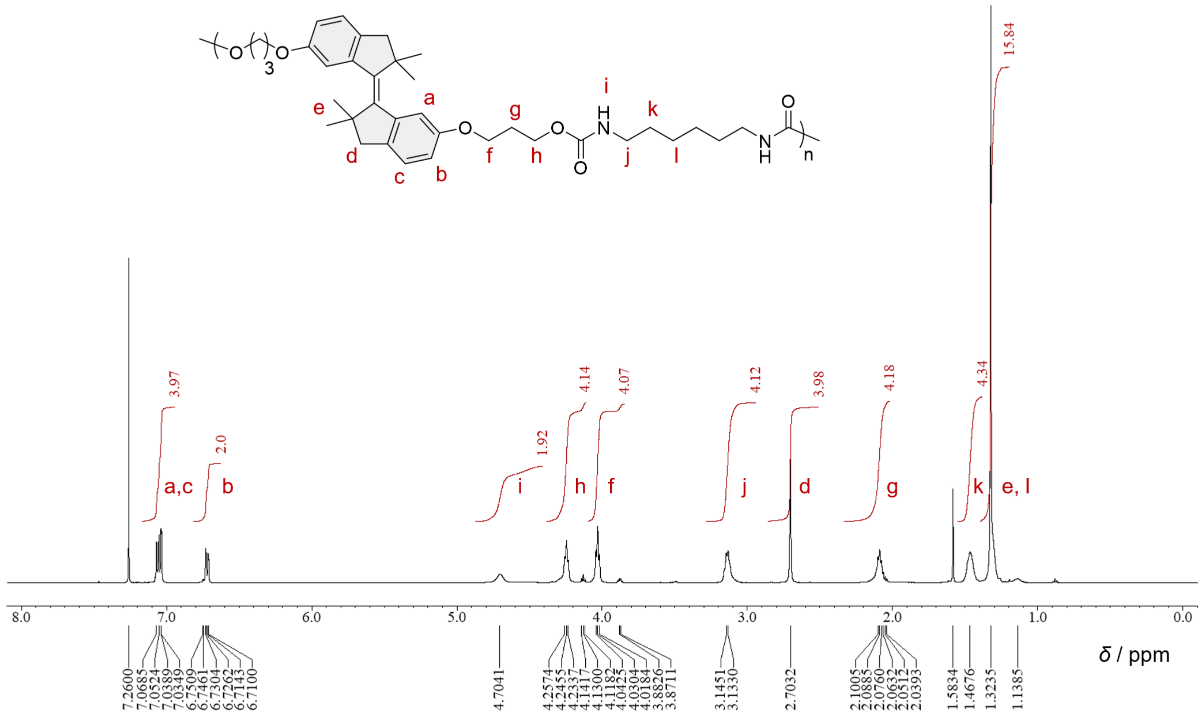

Fig. S1 ^{1}H NMR spectrum (500 MHz, $CDCl_3$) of ***E*-P1**.

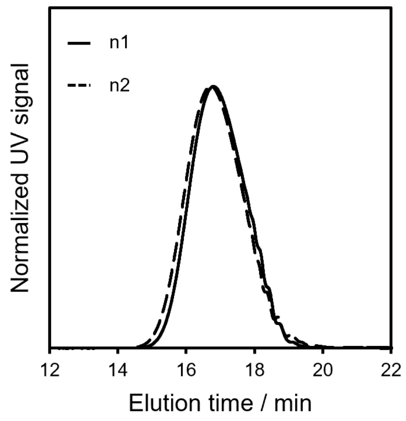

Fig. S2 SEC curves of ***E*-P1**.

Synthesis of ***Z*-P1**

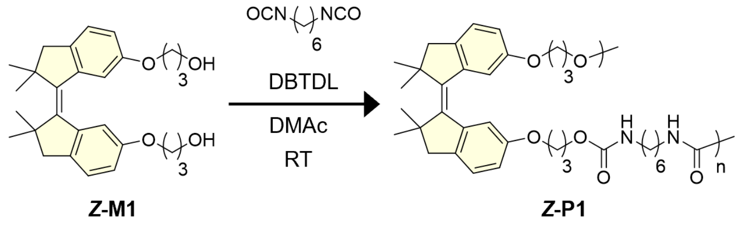

Scheme S2 Synthetic route for ***Z*-P1**.

***Z*-P1**. A solution of ***Z*-M1** (501 mg, 1.15 mmol) and DBTDL (3.40 μL, 5.65 μmol) in dehydrated DMAc (3.1 mL) was prepared under nitrogen atmosphere. HDI (183 μL, 1.14 mmol) was then added dropwise to the solution under nitrogen atmosphere. After stirring at RT for 30 h, methanol (0.5 mL) was added to the reaction mixture and stirred for 3 h. The mixture was precipitated into water, and the precipitate was collected by filtration, dissolved in DCM (3 mL), and reprecipitated into a mixed solvent of hexane (36 mL) and isopropanol (4 mL). The precipitate was collected by filtration and dried under vacuum to give ***Z*-P1** as a pale yellow solid (482 mg, 70% yield). ^{1}H NMR (500 MHz, $CDCl_3$, Fig. S3): δ (ppm) = 7.14–7.08 (m, 2H, aromatic), 7.06–7.00 (m, 2H, aromatic), 6.65–6.58 (m, 2H, aromatic), 5.20–5.00 (m, 2H, NH), 4.15–4.05 (m, 4H, OCH_2), 3.85–3.67 (m, 4H, OCH_2), 3.18–3.01 (m, 6H, $NHCH_2$ and CH_2), 2.46 (d, J = 14.3 Hz, 2H, CH_2), 1.97–1.85 (m, 4H, CH_2), 1.61 (s, 6H, CH_3), 1.53–1.40 (m, 4H, CH_2), 1.36–1.23 (m, 4H, CH_2), 1.19 (s, 6H, CH_3). The M_n and M_w/M_n were determined by SEC (Fig. S4). M_n = 9900 g mol^{-1}, M_w/M_n = 1.59.

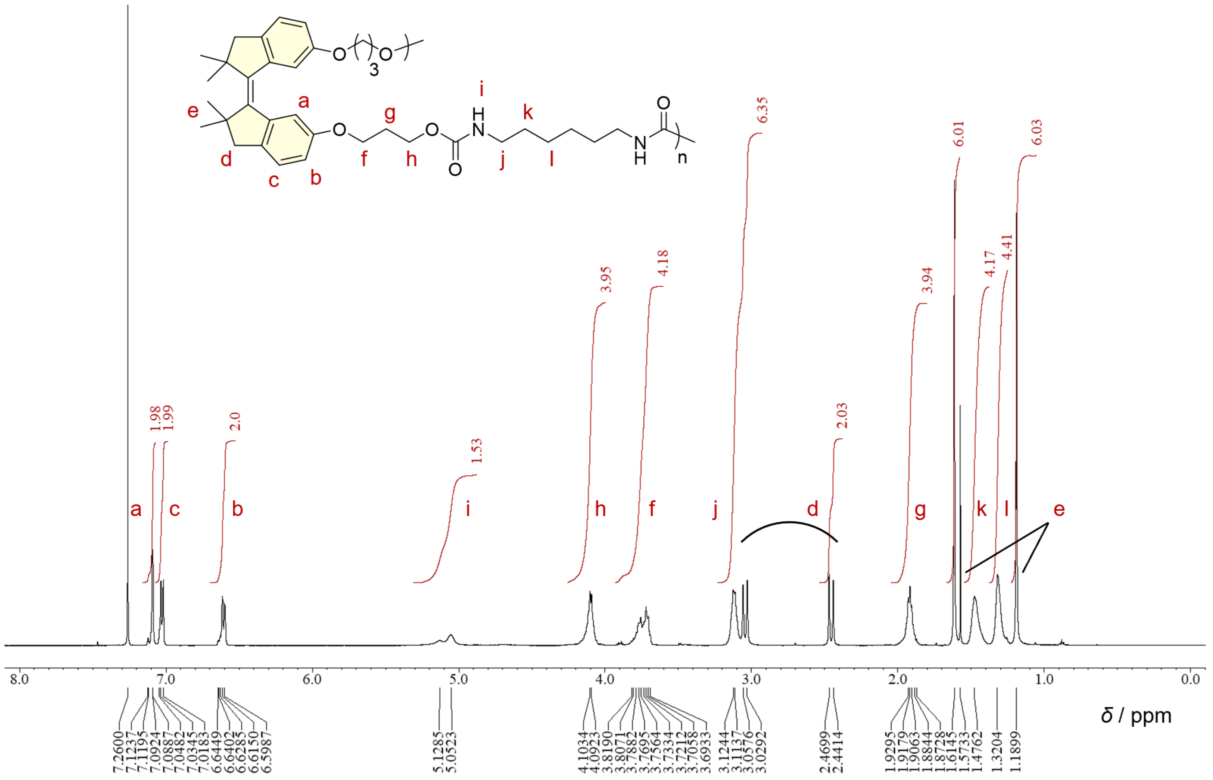

Fig. S3 ^{1}H NMR spectrum (500 MHz, $CDCl_3$) of ***Z*-P1**.

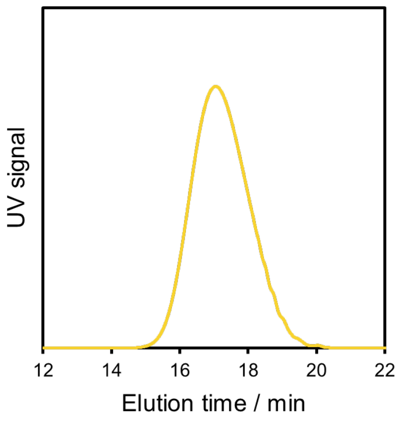

Fig. S4 SEC curve of ***Z*-P1**.

Synthesis of ***E*-P2**

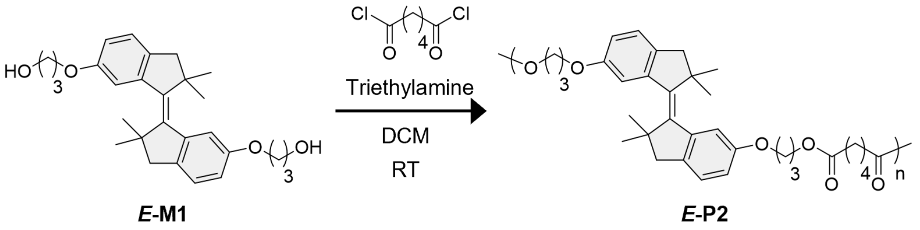

Scheme S3 Synthetic route for ***E*-P2**.

***E*-P2.** A solution of ***E*-M1** (400 mg, 0.92 mmol) and triethylamine (190 µL, 1.37 mmol) in dehydrated DCM (1.6 mL) was prepared under nitrogen atmosphere. Adipoyl chloride (130 µL, 0.89 mmol) was then added dropwise to the solution in an ice bath under nitrogen atmosphere. After stirring at RT for 43 h, adipoyl chloride (8 drops) was added to the reaction mixture to promote polymerization. After stirring at RT for 8 h, methanol (0.3 mL) was added to the reaction mixture and stirred for 3 h. The mixture was diluted with DCM (30 mL), washed with saturated $NaHCO_3$ aqueous solution and water, and concentrated. The crude product was precipitated into a mixed solvent of hexane (30 mL) and methanol (4.5 mL). The precipitate was collected by dissolution in DCM, concentrated, and dried under vacuum to give ***E*-P2** as a white solid (320 mg, 66% yield). ^{1}H NMR (500 MHz, $CDCl_3$, Fig. S5): δ (ppm) = 7.12–7.02 (m, 4H, aromatic), 6.77–6.70 (m, 2H, aromatic), 4.27 (t, J = 6.4 Hz, 4H, OCH_2), 4.03 (t, J = 6.1 Hz, 4H, OCH_2), 2.71 (s, 4H, CH_2), 2.38–2.25 (m, 4H, $COCH_2$), 2.18–2.06 (m, 4H, CH_2), 1.71–1.60 (m, 4H, CH_2), 1.33 (s, 12H, CH_3). The M_n and M_w/M_n were determined by SEC (Fig. S6). M_n = 8000 g mol^{-1}, M_w/M_n = 1.44.

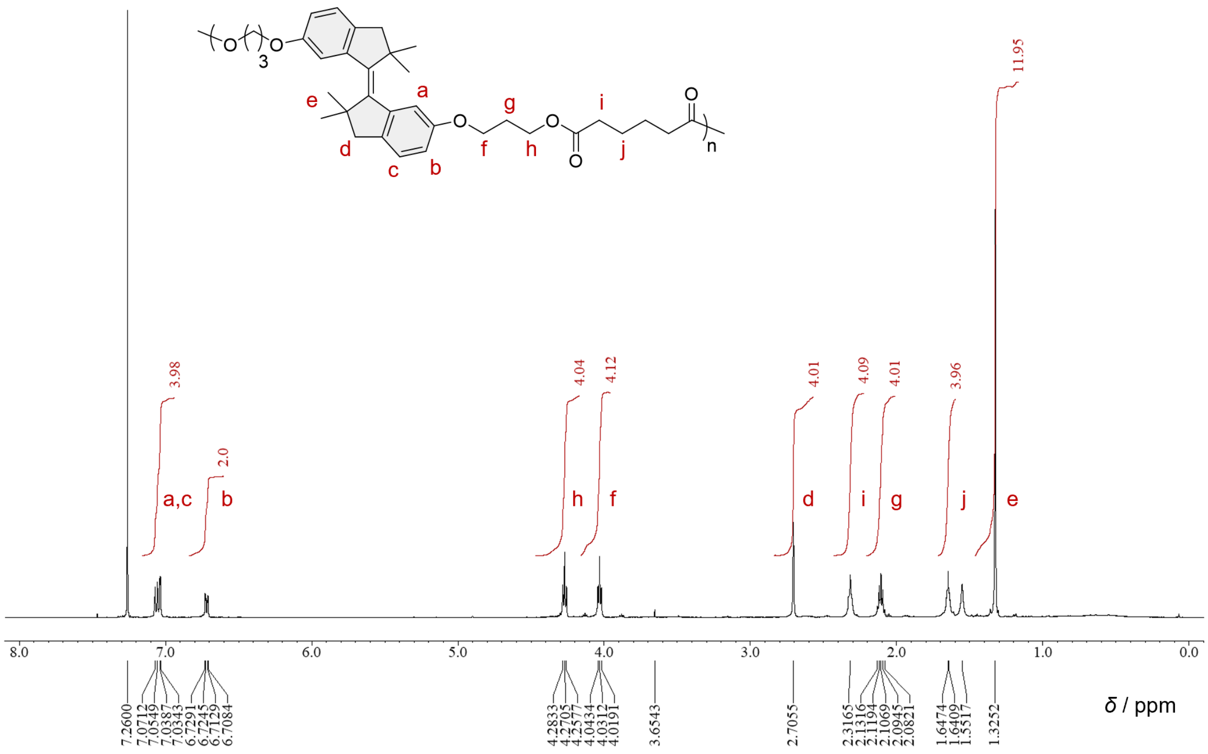

Fig. S5 ^{1}H NMR spectrum (500 MHz, $CDCl_3$) of ***E*-P2**.

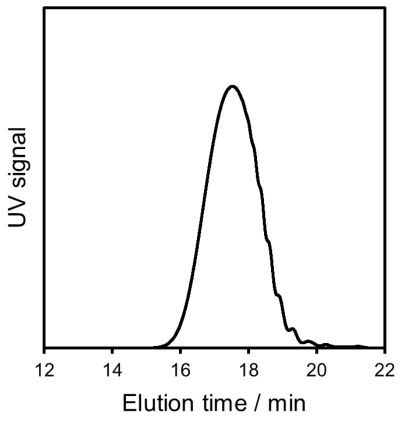

Fig. S6 SEC curve of ***E*-P2**.

Synthesis of *E*-P3

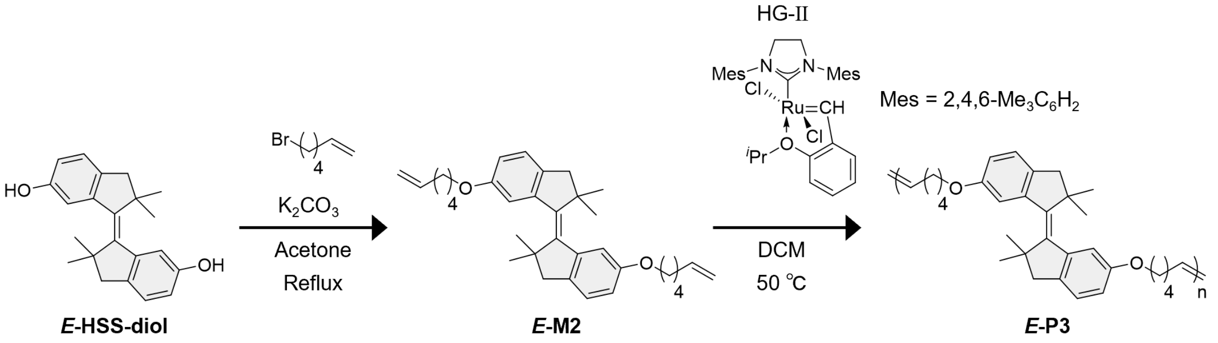

Scheme S4 Synthetic route for ***E*-P3**.

***E*-M2.** A solution of ***E*-HSS-diol** (1.00 g, 3.12 mmol) and potassium carbonate (K_2CO_3, 1.73 g, 12.5 mmol) in dehydrated acetone (20 mL) was prepared under nitrogen atmosphere. 6-Bromo-1-hexene (1.70 mL, 12.7 mmol) was added to the solution under nitrogen atmosphere. The mixture was stirred under reflux for 49 h. After cooling to RT, ethyl acetate was added, and the solution was washed with water and brine, dried over anhydrous $MgSO_4$, filtered, and concentrated. Finally, the crude product was purified by flash column chromatography (silica, hexane/ethyl acetate = 9/1) and dried under vacuum to give ***E*-M2** as a pale yellow liquid (1.05 g, 69% yield). ^{1}H NMR (500 MHz, acetone-d_6, Fig. S7): δ (ppm) = 7.11 (d, J = 8.2 Hz, 2H, aromatic), 7.06 (d, J = 2.4 Hz, 2H, aromatic), 6.78 (dd, J = 8.2 and 2.4 Hz, 2H, aromatic), 5.89–5.80 (m, 2H, CH), 5.06–4.92 (m, 4H, CH_2), 4.02 (t, J = 6.5, 4H, OCH_2), 2.72 (s, 4H, CH_2), 2.17–2.11 (m, 4H, CH_2), 1.83–1.77 (m, 4H, CH_2), 1.62–1.55 (m, 4H, CH_2), 1.34 (s, 12H, CH_3). ^{13}C NMR (125 MHz, acetone-d_6, Fig. S8): δ (ppm) = 158.1, 147.3, 144.8, 139.7, 138.2, 125.7, 115.2, 115.1, 114.7, 68.7, 52.1, 51.9, 34.3, 29.8, 28.2, 26.3. HRMS (APCI) m/z: $[M+H]^+$ Calcd for $C_{34}H_{45}O_2$ 485.34196; Found 485.34131.

***E*-P3.** A solution of ***E*-M2** (400 mg, 0.83 mmol) and a 2nd generation Hoveyda-Grubbs catalyst (HG-II, 7.79 mg, 12.0 μmol) in dehydrated DCM (0.44 mL) was prepared under nitrogen atmosphere. The mixture was stirred at 50 °C, then generated ethylene gas was removed under vacuum in a liquid nitrogen bath for 1 min, and the mixture was stirred at 50 °C again.[2] The procedure was repeated three times in the first 1.5 h and seven times in the following 7.5 h. After cooling to RT, ethyl vinyl ether (0.1 mL) was added to the reaction mixture and stirred for 1 h. The mixture was diluted with DCM and

stirred with Silia*MetS* DMT (metal scavenger, 20 equivalent to HG-II) to remove HG-II for 5 days. The mixture was filtered, and the filtrate was concentrated and precipitated into cold hexane. The precipitate was collected by filtration and dried under vacuum to give ***E*-P3** as a white solid (198 mg, 50% yield). ^{1}H NMR (500 MHz, $CDCl_3$, Fig. S9): δ (ppm) = 7.10–7.02 (m, 4H, aromatic), 6.75–6.69 (m, 2H, aromatic), 5.62–5.37 (m, 2H, CH), 4.00–3.90 (m, 4H, OCH_2), 2.70 (s, 4H, CH_2), 2.60–1.20 (m, 24H, CH_2 and CH_3). The M_n and M_w/M_n were determined by SEC (Fig. S10). M_n = 11900 g mol^{-1}, M_w/M_n = 1.36.

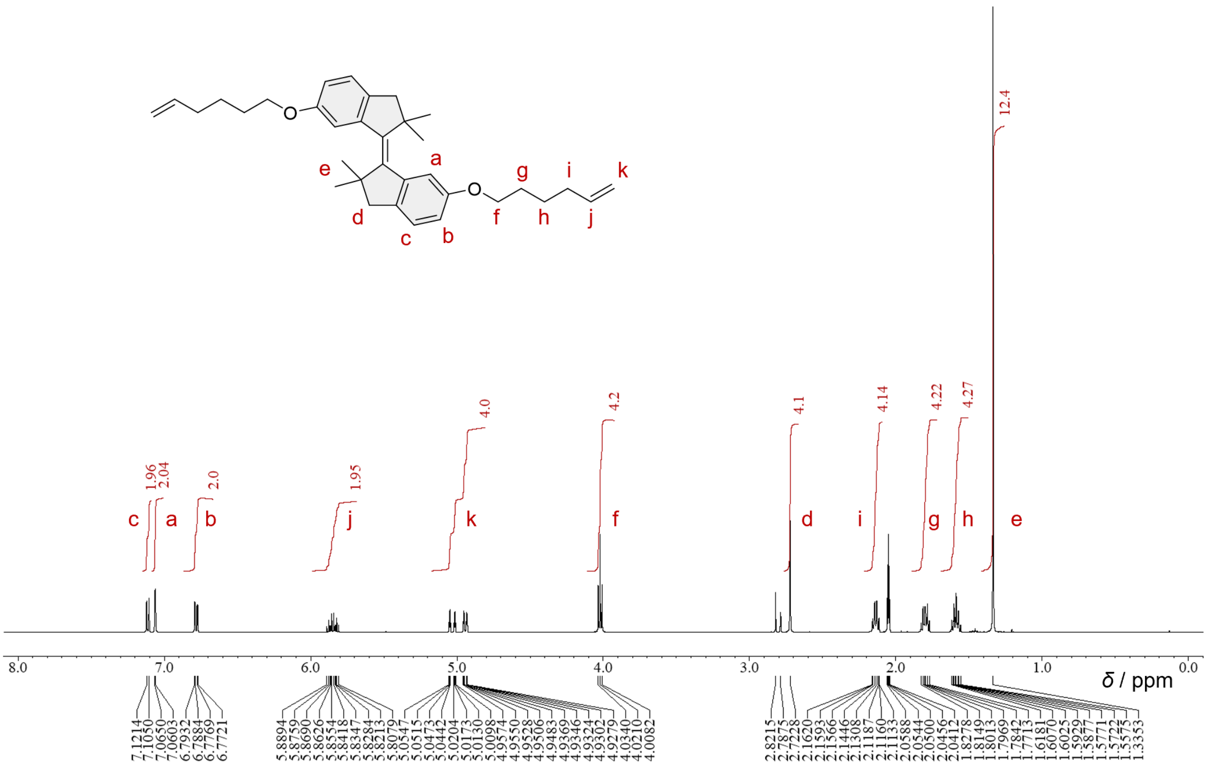

Fig. S7 ^{1}H NMR spectrum (500 MHz, acetone-d_6) of ***E*-M2**.

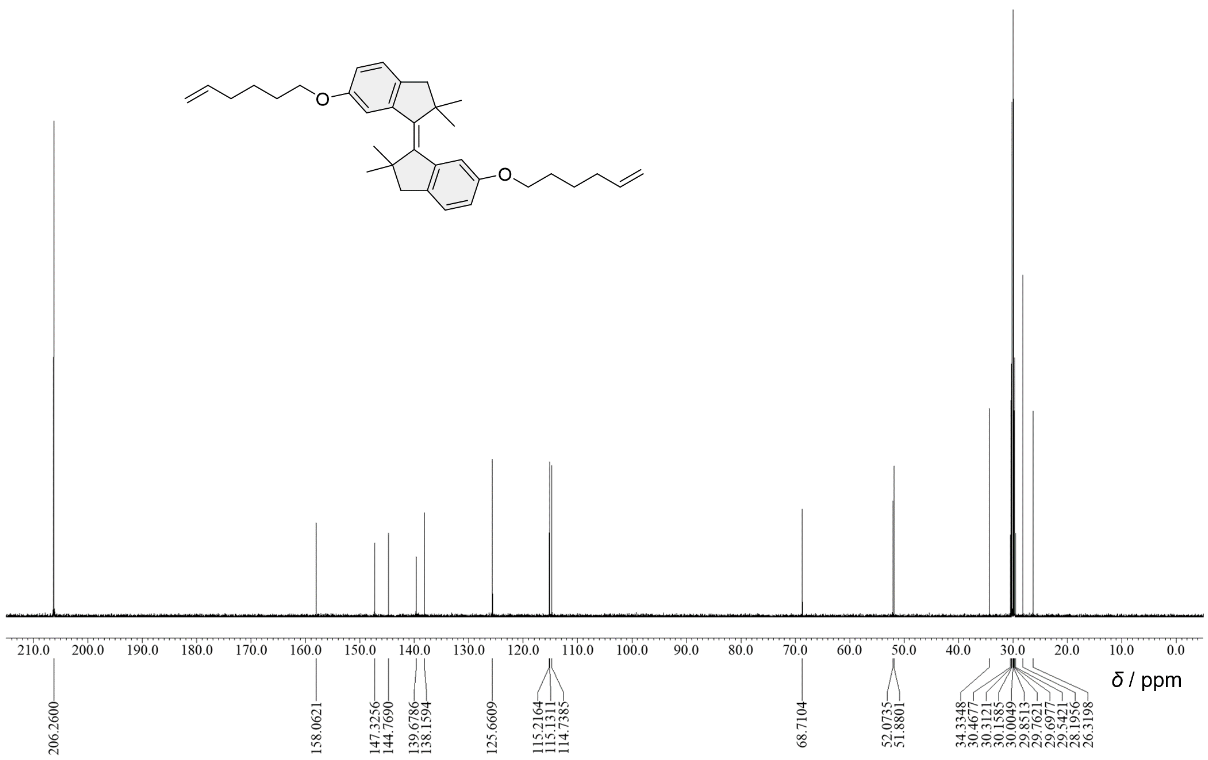

Fig. S8 ^{13}C NMR spectrum (125 MHz, acetone-d_6) of ***E*-M2**.

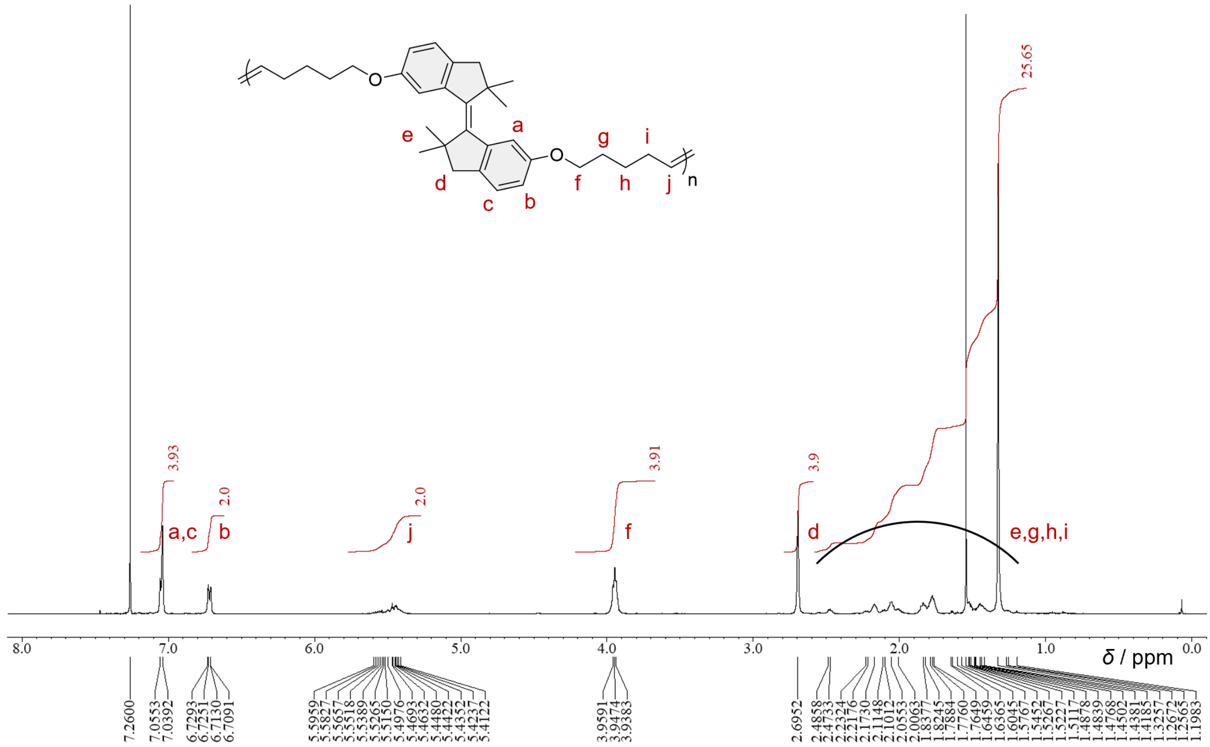

Fig. S9 ^{1}H NMR spectrum (500 MHz, $CDCl_3$) of ***E*-P3**.

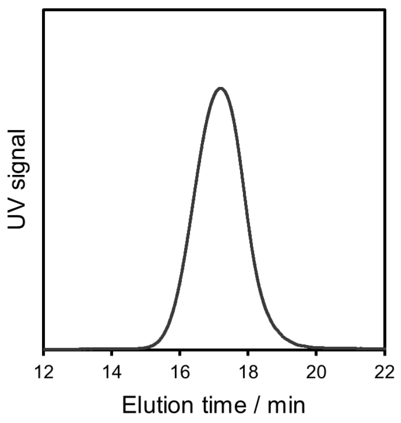

Fig. S10 SEC curve of ***E*-P3**.

Side reactions in polycondensation without triethylamine for *E*-P2

A solution of ***E*-M1** (412 mg, 0.94 mmol) in dehydrated DCM (1.6 mL) was prepared under nitrogen atmosphere. Adipoyl chloride (134 µL, 0.92 mmol) was then added dropwise to the solution in an ice bath under nitrogen atmosphere. After stirring at RT for 3 days, methanol (0.3 mL) was added to the reaction mixture and stirred for 3 h. The mixture was diluted with DCM (30 mL), washed with saturated $NaHCO_3$ aqueous solution and water, and concentrated. The crude product was precipitated into a mixed solvent of hexane (30 mL) and methanol (4.5 mL). The precipitate was collected by dissolution in DCM, concentrated, and dried under vacuum. A ^{1}H NMR spectrum of the reaction product was measured (Fig. S11).

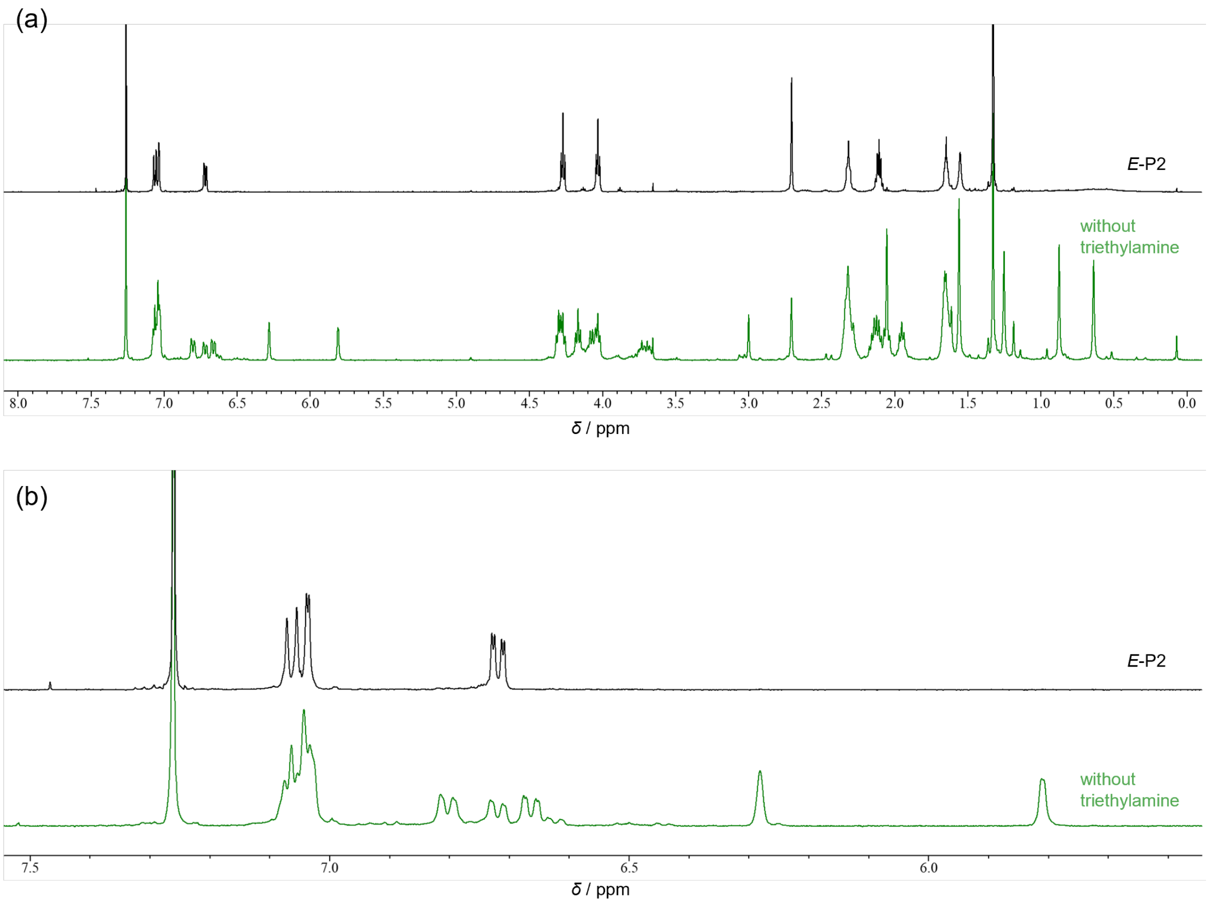

Fig. S11 (a) Overall and (b) enlarged ^{1}H NMR spectra (400 MHz, $CDCl_3$) of ***E*-P2** and the reaction product without triethylamine.

Photoisomerization in solution

UV/vis absorption and ^{1}H NMR spectroscopy was employed to study photoisomerization of the main-chain HSS photoswitches in solution. For UV/vis absorption measurements, solutions of ***E*-P1** (M_n = 12900), ***E*-P2**, and ***E*-P3** (3.00×10^{-5} M) in spectrophotometric grade THF were prepared in a 1.0 cm quartz cuvette with a stirring bar. For ^{1}H NMR measurements, solutions of the polymers (1.00×10^{-4} M) in THF-d_8 were prepared in a 1.0 cm quartz cuvette with a stirring bar. The solutions were irradiated with xenon light (MAX-303, Asahi Spectra) with a 340 nm band pass filter (LX0340, Asahi Spectra) and light emitting diode (LED) light with a peak wavelength of 405 nm (CL-H1-405-9-1-B, Asahi Spectra) under stirring (Fig. S12). The irradiance was determined using an optical power meter (PM100USB and S401C, Thorlabs) prior to light irradiation (Table S1). The *E*/*Z* ratios were determined from UV/vis absorption spectra (Fig. 3, S13, and S14) using molar absorption coefficients (εs) of the *E* and *Z* isomers and ^{1}H NMR spectra (Fig. 3, S13, and S14).

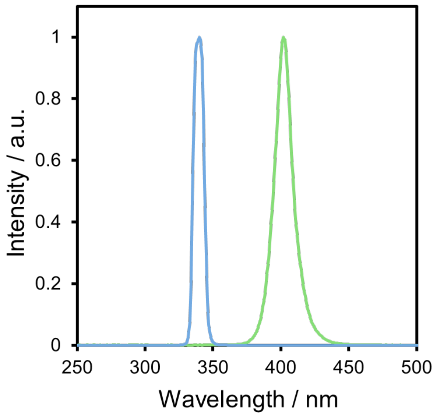

Fig. S12 Normalized emission spectra of xenon light with 340 nm band pass filter and 405 nm LED light.

Table S1 Irradiance of xenon light with 340 nm band pass filter and 405 nm LED light used for photoisomerization

	***E*-P1**		***E*-P2**		***E*-P3**	
	UV/vis	^{1}H NMR	UV/vis	^{1}H NMR	UV/vis	^{1}H NMR
340 nm / mW cm^{-2}	51.6	31.3	28.2	31.3	27.1	31.3
405 nm / mW cm^{-2}	666	637	634	637	566	637

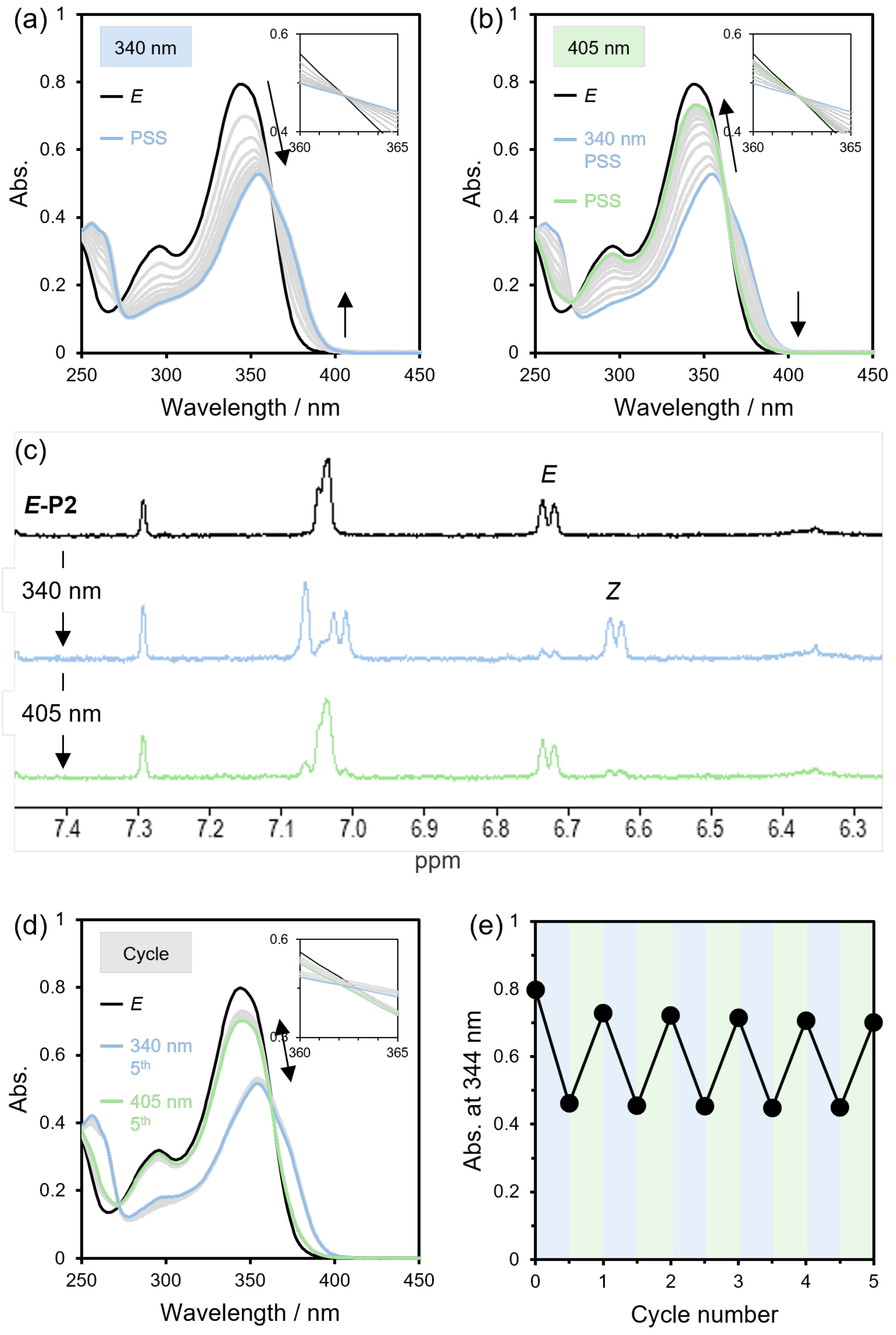

Fig. S13 UV/vis absorption spectra of ***E*-P2** upon (a) irradiation with 340 nm light and (b) subsequent irradiation with 405 nm light in THF (3.00×10^{-5} M). (c) ^{1}H NMR spectra (500 MHz, THF-d_8) of ***E*-P2** and PSSs under 340 and 405 nm irradiation in THF-d_8 (1.00×10^{-4} M). (d) UV/vis absorption spectra and (e) changes in absorbance at 344 nm upon alternating irradiation of ***E*-P2** with 340 and 405 nm light in THF (3.00×10^{-5} M).

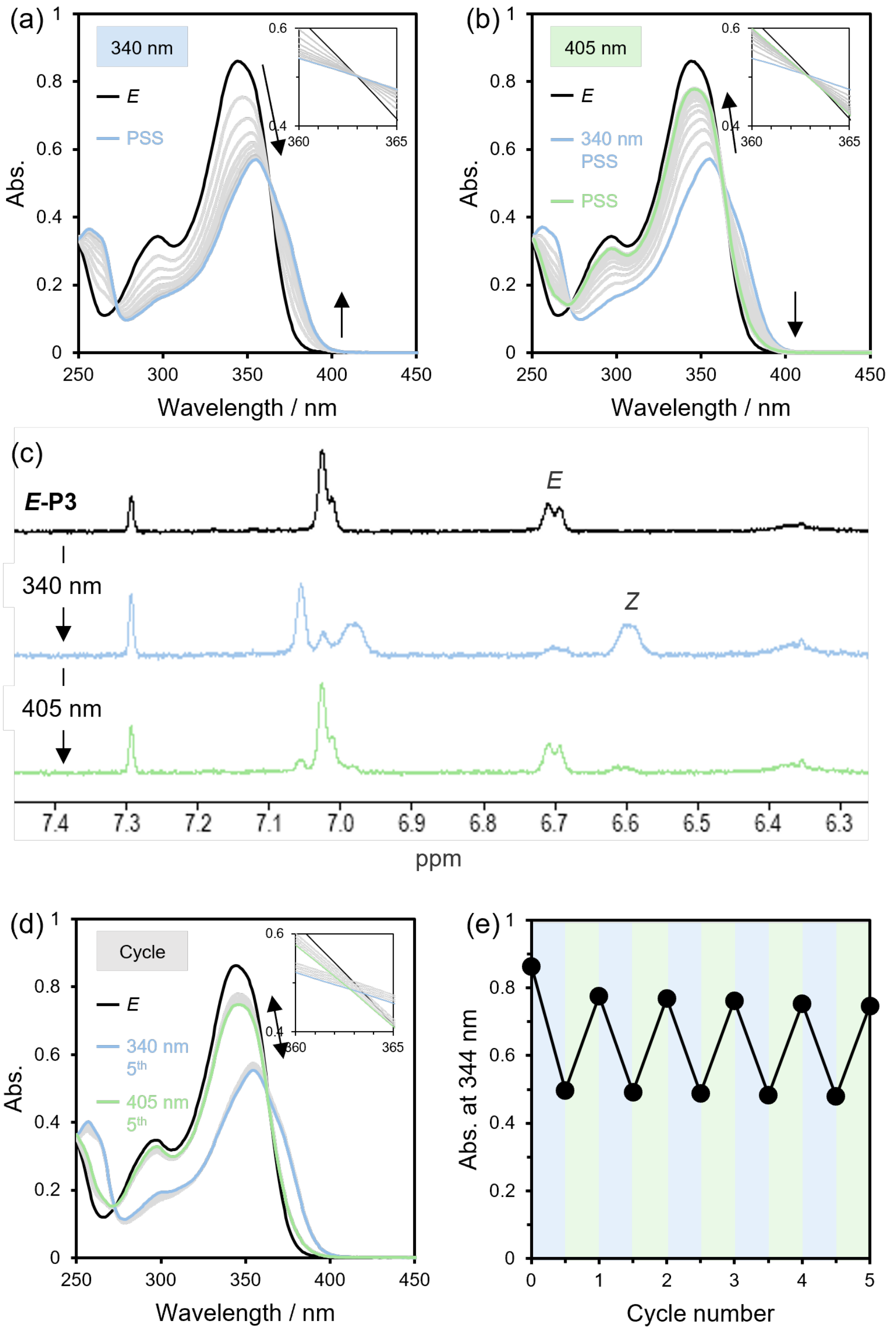

Fig. S14 UV/vis absorption spectra of ***E*-P3** upon (a) irradiation with 340 nm light and (b) subsequent irradiation with 405 nm light in THF (3.00×10^{-5} M). (c) ^{1}H NMR spectra (500 MHz, THF-d_8) of ***E*-P3** and PSSs under 340 and 405 nm irradiation in THF-d_8 (1.00×10^{-4} M). (d) UV/vis absorption spectra and (e) changes in absorbance at 344 nm upon alternating irradiation of ***E*-P3** with 340 and 405 nm light in THF (3.00×10^{-5} M).

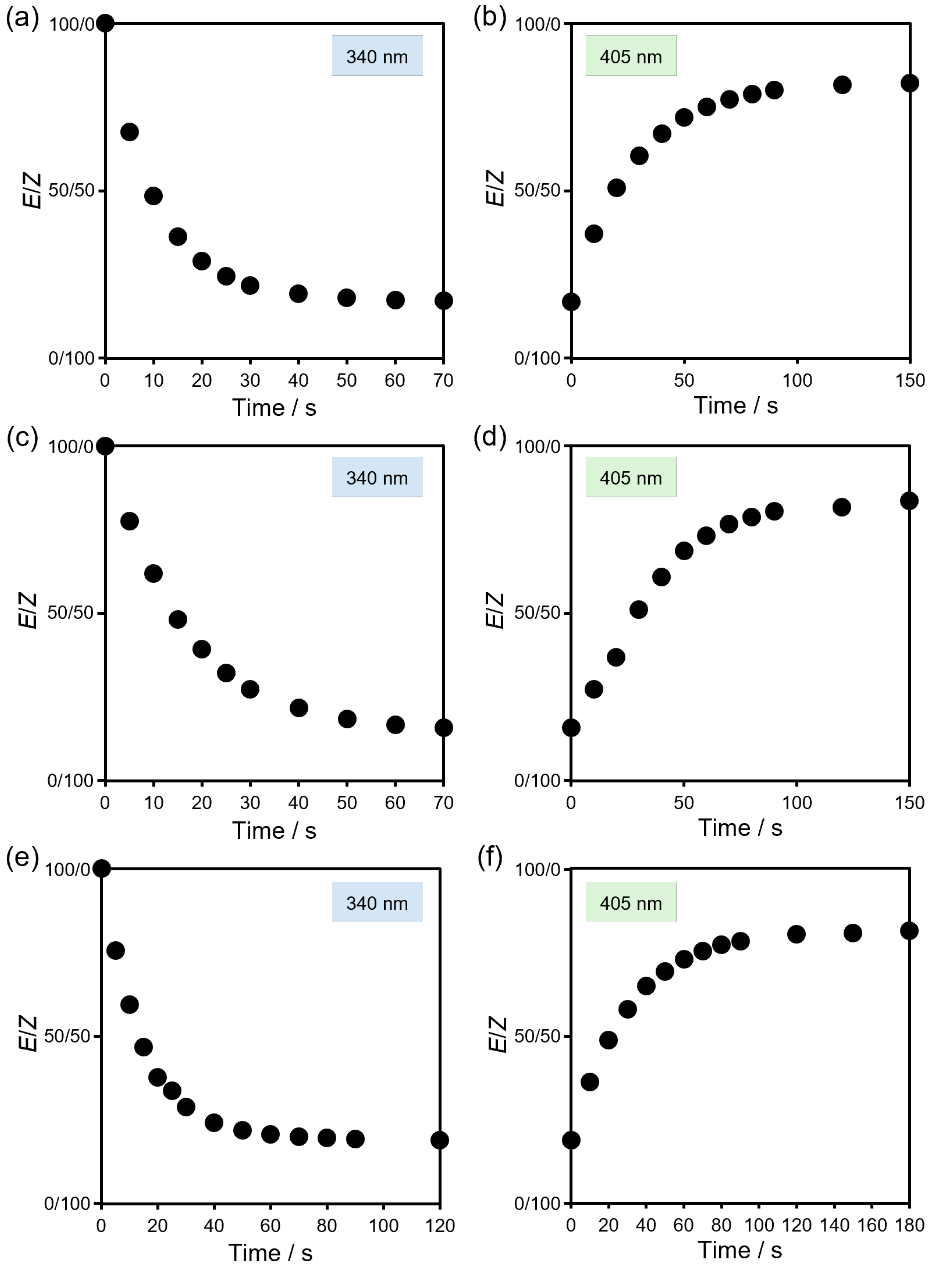

Fig. S15 Changes in *E*/*Z* ratios upon irradiation of ***E*-P1** (M_n = 12900) with (a) 340 nm light and (b) subsequent 405 nm light, ***E*-P2** with (c) 340 nm light and (d) subsequent 405 nm light, and ***E*-P3** with (e) 340 nm light and (f) subsequent 405 nm light in THF (3.00 × 10^{-5} M).

DOSY NMR measurements

Z-rich **P1** (M_n = 13200), **P2**, and **P3** were prepared by exposure of their 100% *E* polymers in THF (1.00 $\times 10^{-3}$ M) to 300 nm LED light (0.40 mW cm^{-2}, LDR2-100UV300, CCS) (Fig. S29) under stirring and drying in vacuum.

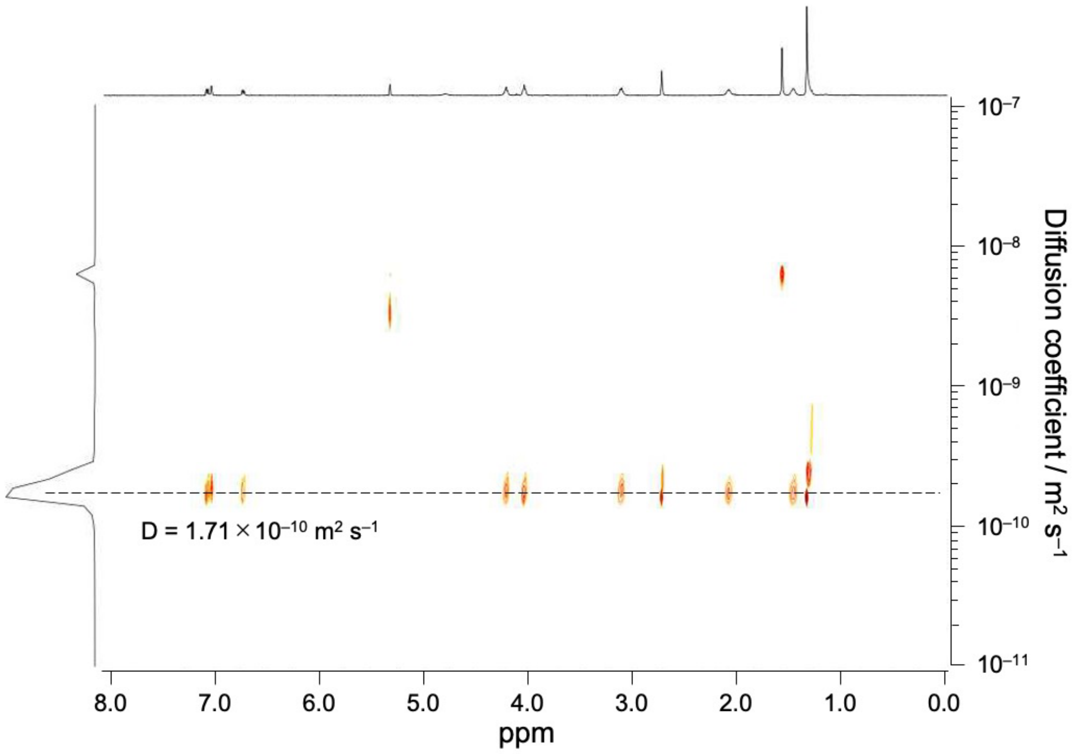

Fig. S16 DOSY NMR spectrum (500 MHz, CD_2Cl_2) of ***E*-P1** (M_n = 13200).

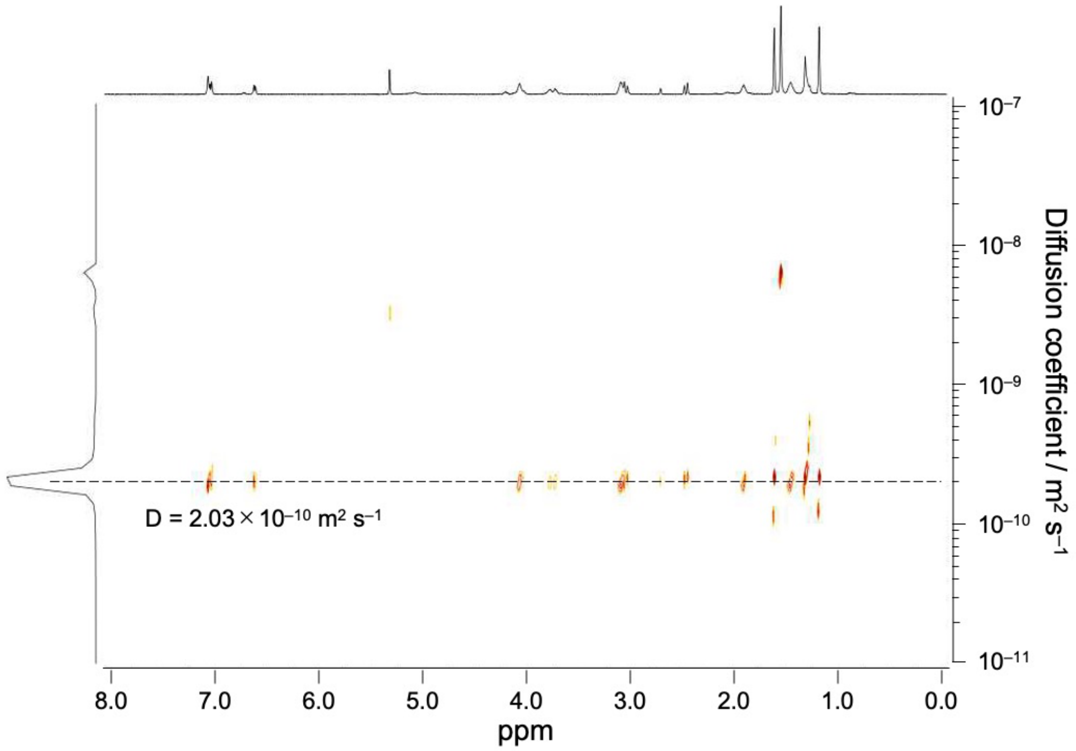

Fig. S17 DOSY NMR spectrum (500 MHz, CD_2Cl_2) of *Z*-rich **P1** (84% *Z*) prepared by exposure of ***E*-P1** (M_n = 13200) in THF (1.00×10^{-3} M) to 300 nm light.

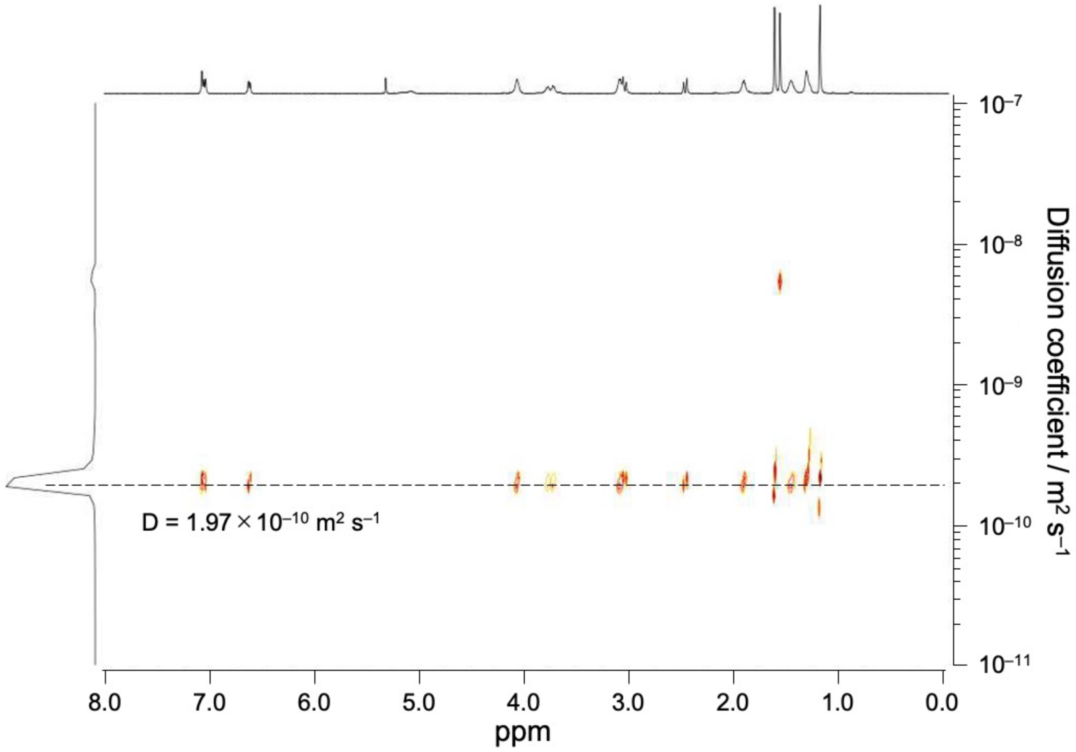

Fig. S18 DOSY NMR spectrum (500 MHz, CD_2Cl_2) of ***Z*-P1**.

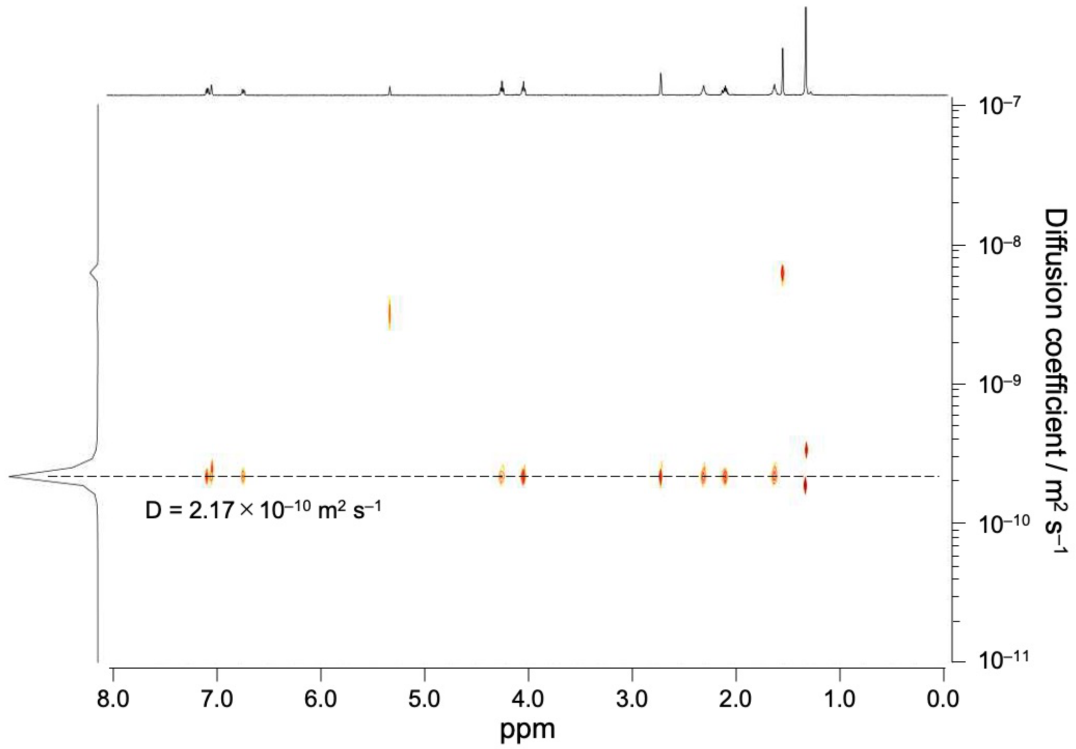

Fig. S19 DOSY NMR spectrum (500 MHz, CD_2Cl_2) of ***E*-P2**.

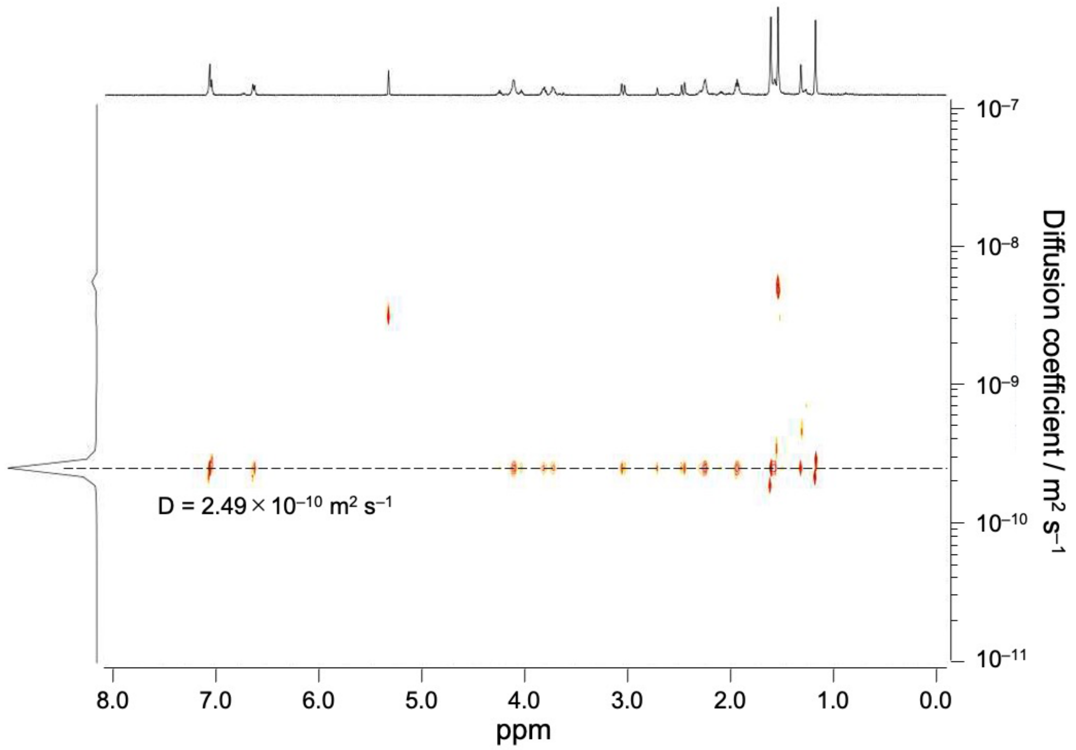

Fig. S20 DOSY NMR spectrum (500 MHz, CD_2Cl_2) of *Z*-rich **P2** (84% *Z*) prepared by exposure of ***E*-P2** in THF (1.00×10^{-3} M) to 300 nm light.

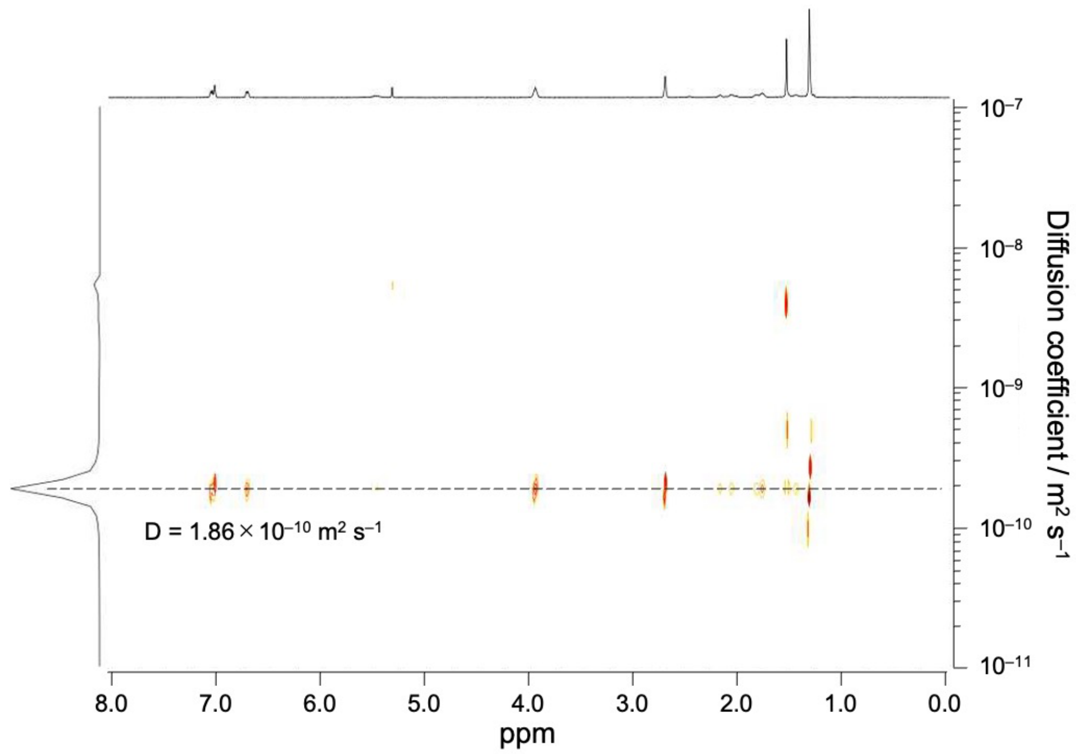

Fig. S21 DOSY NMR spectrum (500 MHz, CD_2Cl_2) of ***E*-P3**.

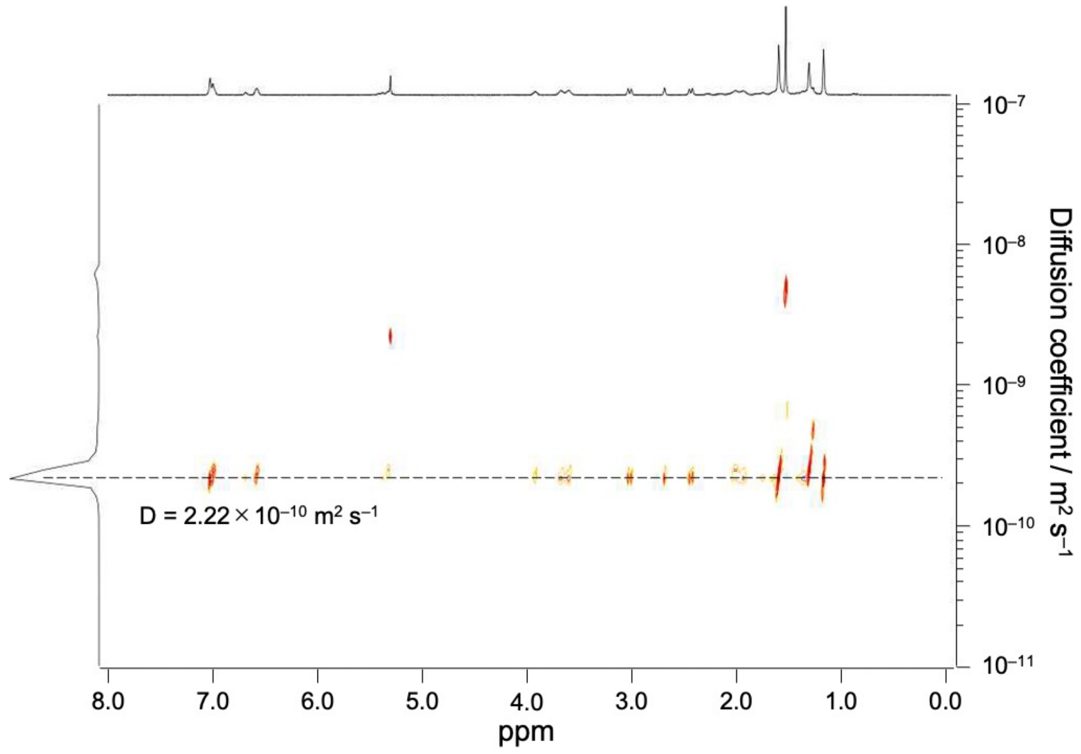

Fig. S22 DOSY NMR spectrum (500 MHz, CD_2Cl_2) of *Z*-rich **P3** (80% *Z*) prepared by exposure of ***E*-P3** in THF (1.00×10^{-3} M) to 300 nm light.

Macromolecular conformational changes in solution

SEC measurements were employed to study conformational changes of the single polymer chains in solution induced by photoisomerization of the main-chain HSS photoswitches. Solutions of ***E*-P1** (M_n = 12900), ***E*-P2**, and ***E*-P3** (3.00×10^{-5} M) in spectrophotometric grade THF were prepared in a 1.0 cm quartz cuvette with a stirring bar. The solutions were irradiated with 340 and 405 nm light in the same conditions as the above UV/vis absorption measurements (Fig. S12 and Table S1).

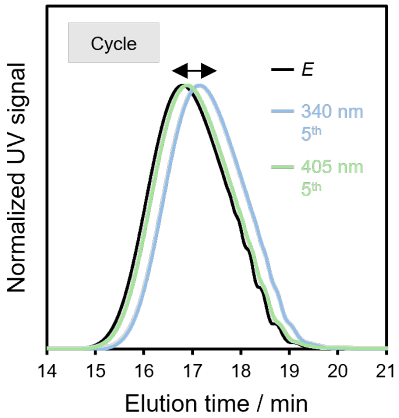

Fig. S23 SEC curves upon alternating irradiation of ***E*-P1** (M_n = 12900) with 340 and 405 nm light in THF (3.00×10^{-5} M).

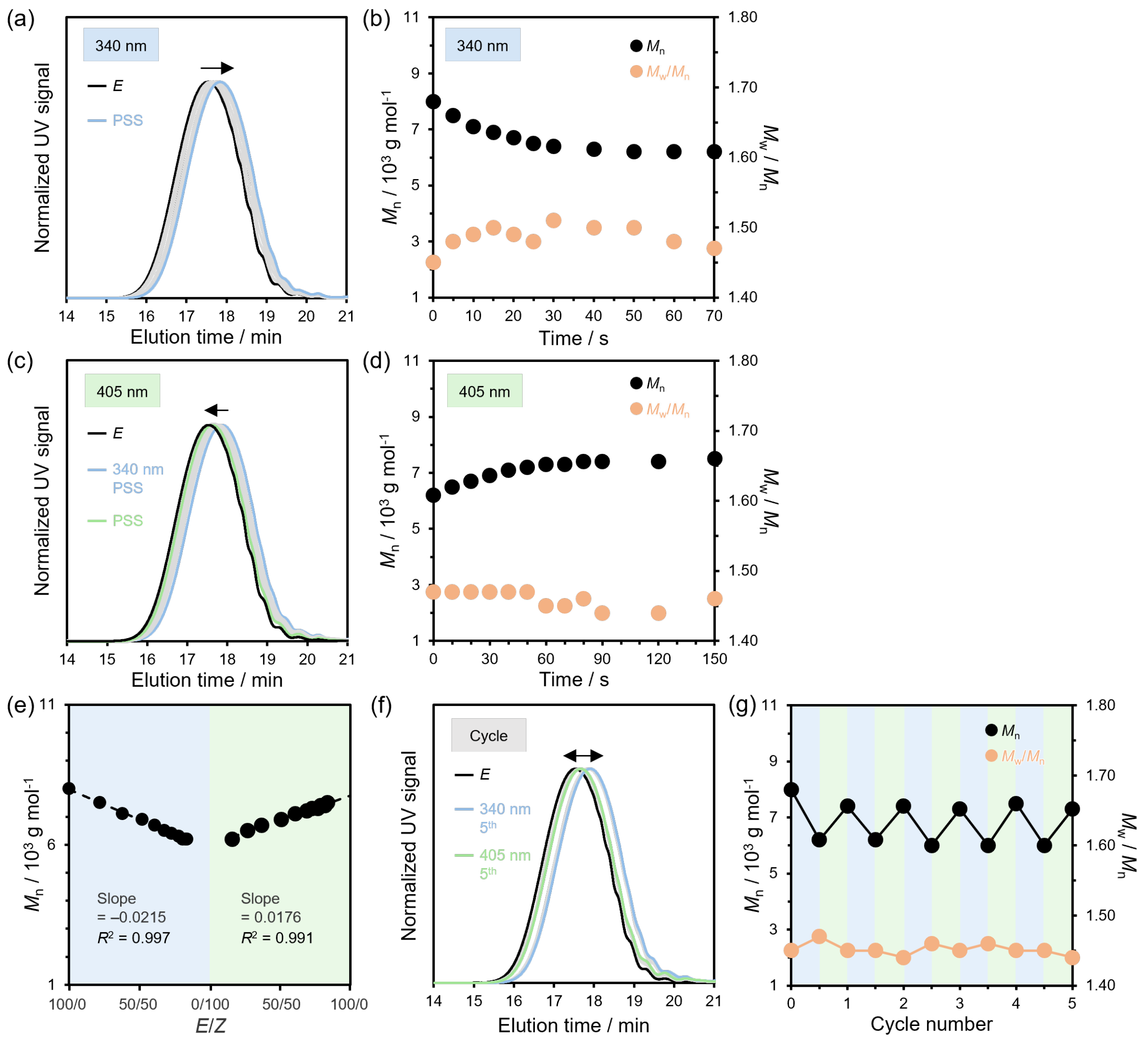

Fig. S24 (a) SEC curves and (b) apparent M_ns and M_w/M_ns of ***E*-P2** during irradiation with 340 nm light in THF (3.00×10^{-5} M). (c) SEC curves and (d) apparent M_ns and M_w/M_ns of 340 nm PSS during irradiation with 405 nm light in THF (3.00×10^{-5} M). (e) Apparent M_ns at different *E*/*Z* ratios determined from UV/vis absorption and ^{1}H NMR spectra in irradiation of ***E*-P2** with 340 nm light and subsequent irradiation of 340 nm PSS with 405 nm light in THF (3.00×10^{-5} M). (f) SEC curves and (g) changes in apparent M_ns and M_w/M_ns upon alternating irradiation of ***E*-P2** with 340 and 405 nm light in THF (3.00×10^{-5} M).

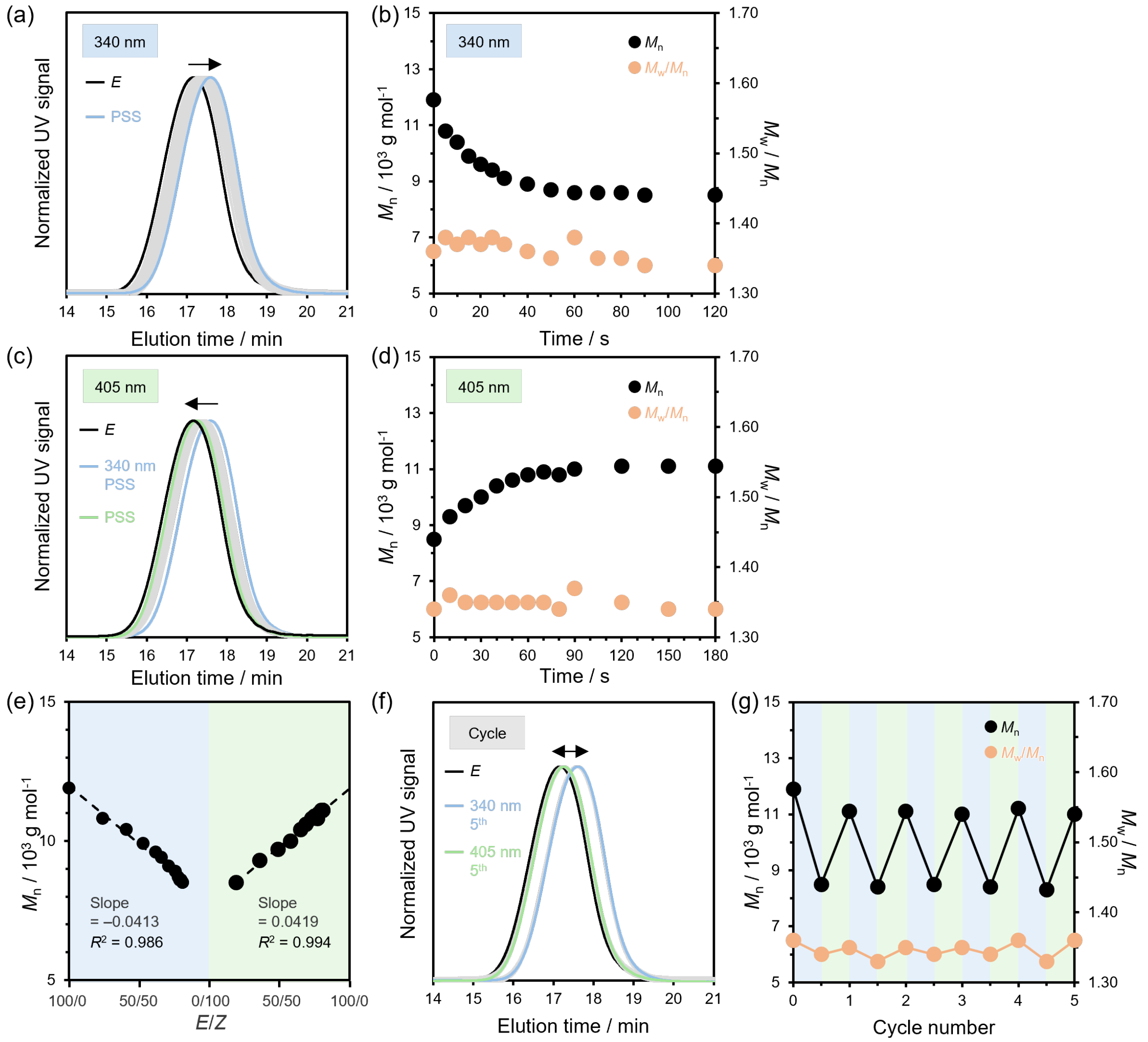

Fig. S25 (a) SEC curves and (b) apparent M_ns and M_w/M_ns of ***E*-P3** during irradiation with 340 nm light in THF (3.00×10^{-5} M). (c) SEC curves and (d) apparent M_ns and M_w/M_ns of 340 nm PSS during irradiation with 405 nm light in THF (3.00×10^{-5} M). (e) Apparent M_ns at different *E*/*Z* ratios determined from UV/vis absorption and ^{1}H NMR spectra in irradiation of ***E*-P3** with 340 nm light and subsequent irradiation of 340 nm PSS with 405 nm light in THF (3.00×10^{-5} M). (f) SEC curves and (g) changes in apparent M_ns and M_w/M_ns upon alternating irradiation of ***E*-P3** with 340 and 405 nm light in THF (3.00×10^{-5} M).

Hydrogen bonding in P1

FTIR, ^{1}H NMR, and UV/vis absorption spectroscopy was employed to study hydrogen bonding of the urethane linkages of **P1** in solution and the ***E*-P1** powder. Transmittance measurements were employed to study solubilities of **P1**.

For FTIR measurements, solutions of ***E*-P1** (M_n = 13200) and ***Z*-P1** (100 mg mL^{-1}) in spectrophotometric grade DCM, chloroform, THF, and toluene were prepared. The toluene solution of ***Z*-P1** (0.90 mM) in a 0.1 cm quartz cuvette was irradiated with LED light with a peak wavelength of 405 nm (19.6 mW cm^{-2}, LDR2-100VL405-W1U, CCS) (Fig. S29) and concentrated. The exposure distance was 80 mm.

For ^{1}H NMR measurements, solutions of ***E*-P1** (M_n = 12900 and 13200) and ***Z*-P1** in CD_2Cl_2, $CDCl_3$, THF-d_8, and toluene-d_8 were prepared. The toluene-d_8 solution of ***Z*-P1** (0.90 mM) in a quartz NMR tube was irradiated with LED light with a peak wavelength of 405 nm (19.6 mW cm^{-2}, LDR2-100VL405-W1U, CCS) (Fig. S29). The exposure distance was 80 mm.

For transmittance measurements, solutions of ***Z*-P1** (0.10, 0.30, and 0.90 mM) in spectrophotometric grade toluene were prepared in a 1.0 cm quartz cuvette. The solutions were irradiated with LED light with a peak wavelength of 300 nm (0.40 mW cm^{-2}, LDR2-100UV300, CCS) and 405 nm (19.6 mW cm^{-2}, LDR2-100VL405-W1U, CCS) (Fig. S29). The exposure distance was 40 mm for the 300 nm light and 80 mm for the 405 nm light. Transmittance of the solutions was monitored at 600 nm, where the main-chain HSS photoswitches have no absorption. The light irradiation was continued until the transmittance reached a stationary state.

For UV/vis absorption measurements, solution of ***Z*-P1** (0.10, 0.30, and 0.90 mM) in spectrophotometric grade toluene were prepared in a 0.1 cm quartz cuvette. The solutions were irradiated with LED light with a peak wavelength of 405 nm (19.6 mW cm^{-2}, LDR2-100VL405-W1U, CCS) (Fig. S29). The exposure distance was 80 mm. The *E*/*Z* ratios were determined from UV/vis absorption spectra (Fig. S31) using εs of the *E* and *Z* isomers.

For supporting videos (Video S1–S3), solutions of ***Z*-P1** (0.90 mM) in spectrophotometric grade toluene were prepared in a 1.0 cm quartz cuvette. The solutions were irradiated with LED light with a peak wavelength of 405 nm (CL-H1-405-9-1-B, Asahi Spectra) and then xenon light (MAX-303, Asahi

Spectra) with a 300 nm band pass filter (LX0300, Asahi Spectra) or LED light with a peak wavelength of 365 nm (CL-H1-365-9-1-B, Asahi Spectra).

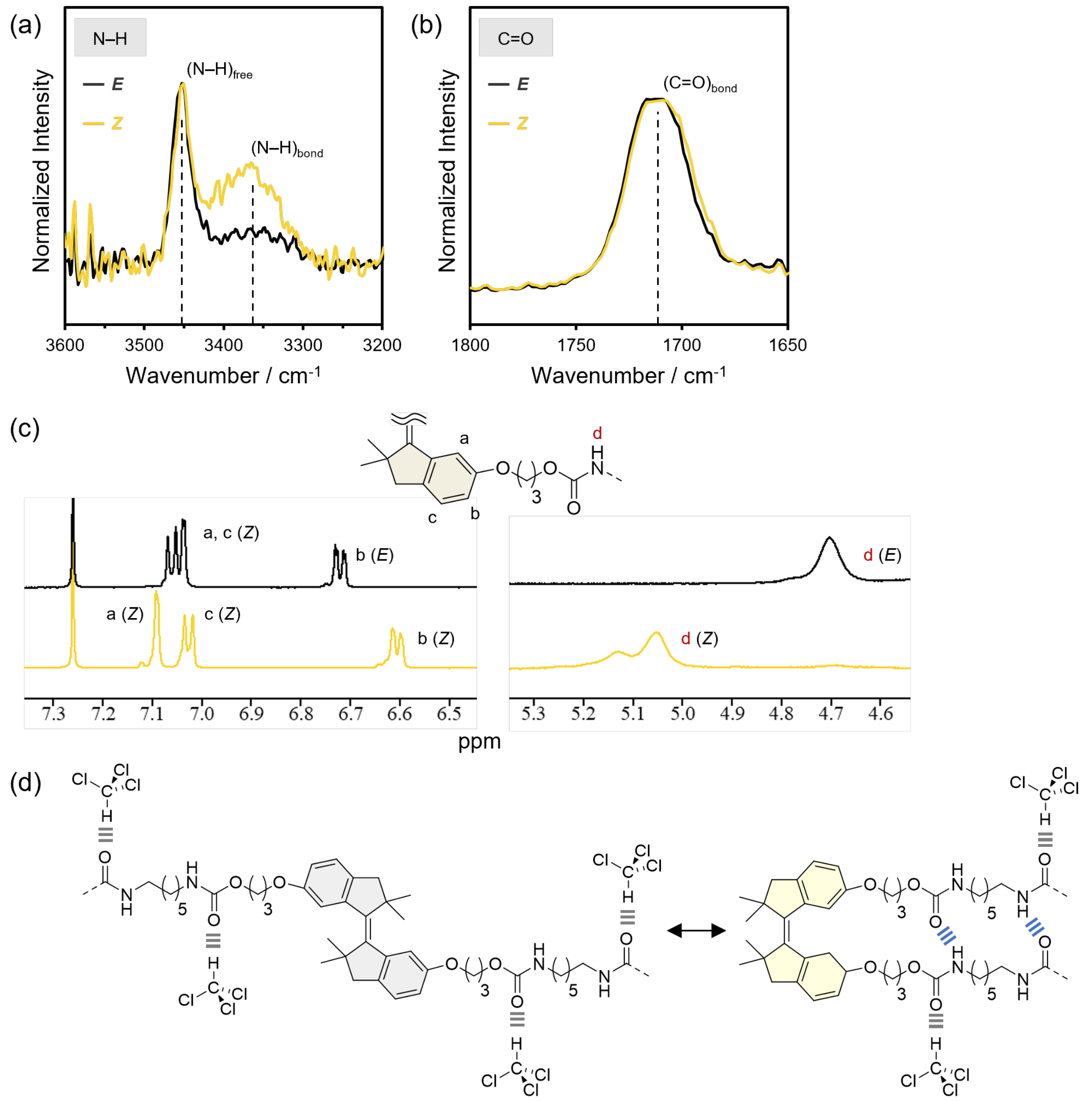

Fig. S26 FTIR spectra of ***E*-P1** (M_n = 13200) and ***Z*-P1** in chloroform (100 mg mL^{-1}) focused on (a) N–H and (b) C=O stretching vibration. (c) ^1H NMR spectra (500 MHz) of ***E*-P1** (M_n = 13200) and ***Z*-P1** in $CDCl_3$. (d) Illustration of hydrogen bonding between urethane linkages and chloroform (gray) in ***E*-P1** and intrachain hydrogen bonding between urethane linkages (blue) in ***Z*-P1**.

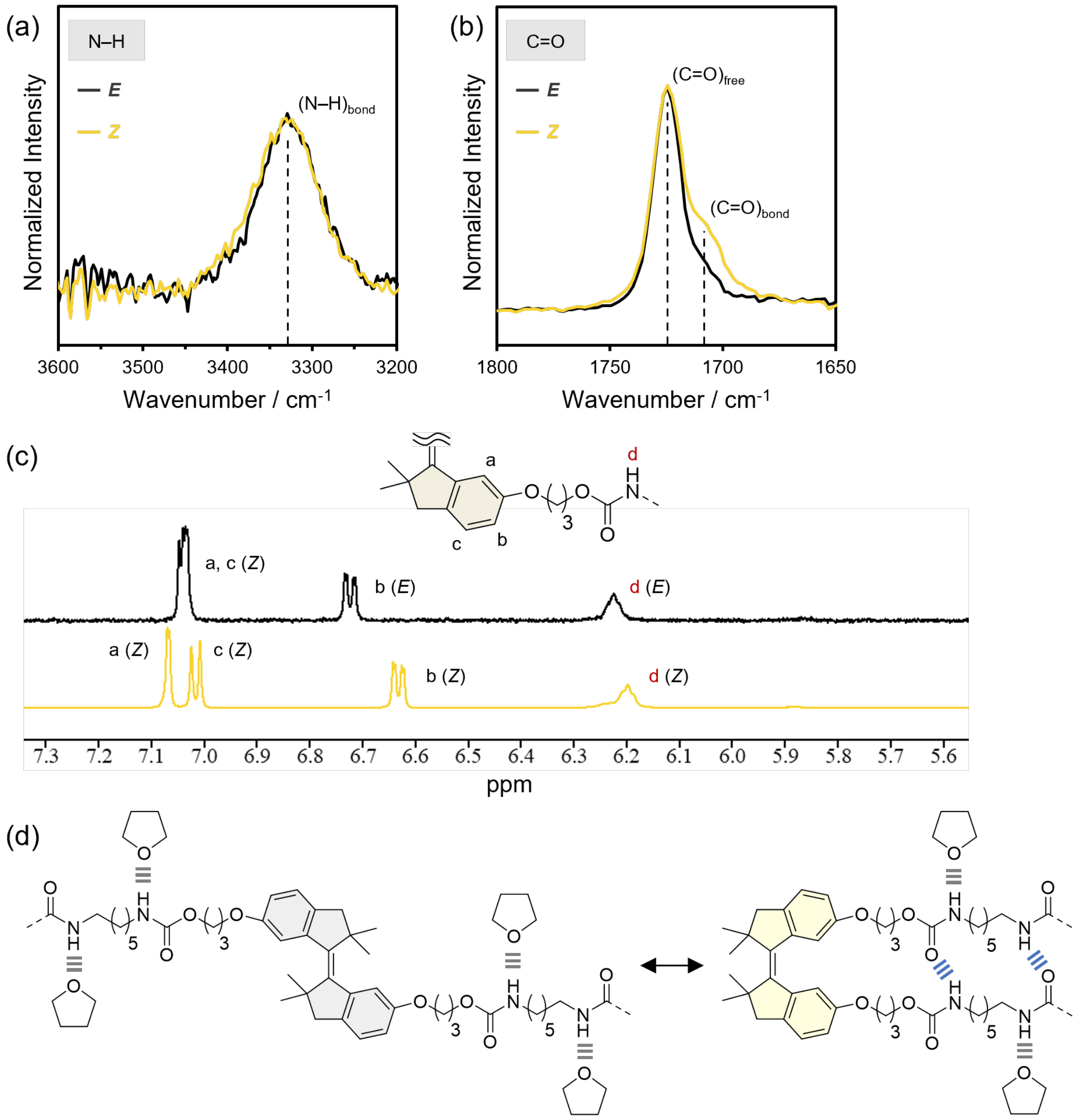

Fig. S27 FTIR spectra of ***E*-P1** (M_n = 13200) and ***Z*-P1** in THF (100 mg mL^{-1}) focused on (a) N–H and (b) C=O stretching vibration. (c) ^1H NMR spectra (500 MHz) of ***E*-P1** (M_n = 12900) and ***Z*-P1** in THF-d_8. (d) Illustration of hydrogen bonding between urethane linkages and THF (gray) in ***E*-P1** and intrachain hydrogen bonding between urethane linkages (blue) in ***Z*-P1**.

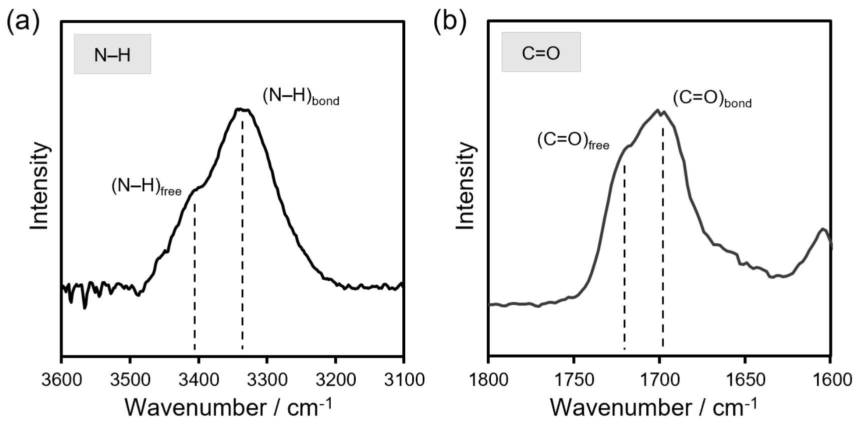

Fig. S28 FTIR spectrum of ***E*-P1** powder (M_n = 13200) focused on (a) N–H and (b) C=O stretching vibration.

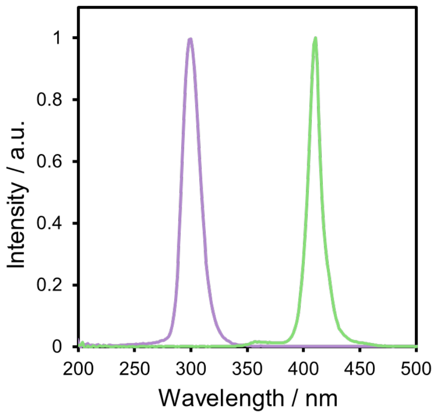

Fig. S29 Normalized emission spectra of LED light with a peak wavelength of 300 or 405 nm.

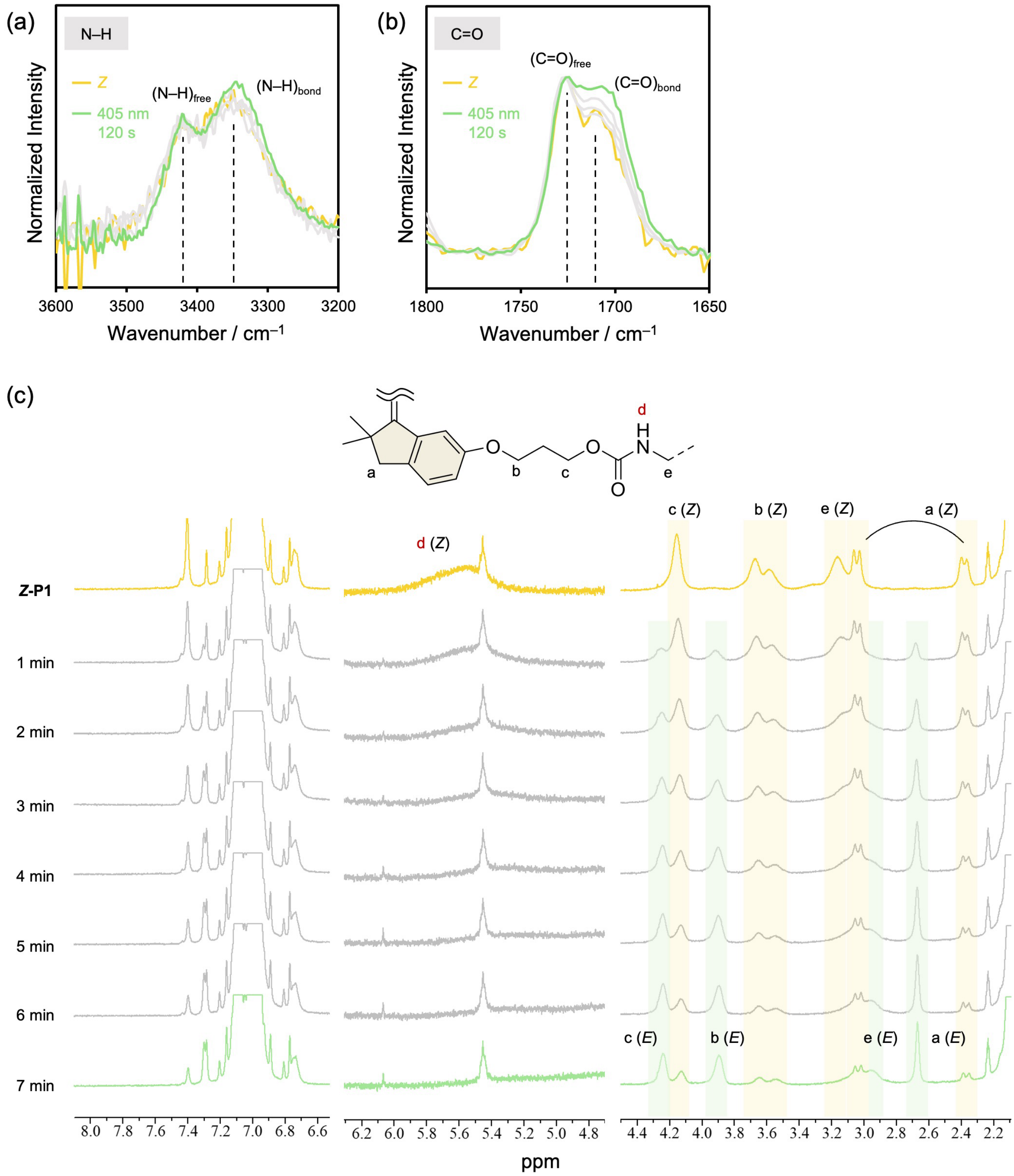

Fig. S30 Changes in FTIR spectra of ***Z*-P1** in toluene focused on (a) N–H and (b) C=O stretching vibration and (c) ^{1}H NMR spectra (400 MHz) of ***Z*-P1** in toluene-d_8 (0.90 mM) under 405 nm light irradiation.

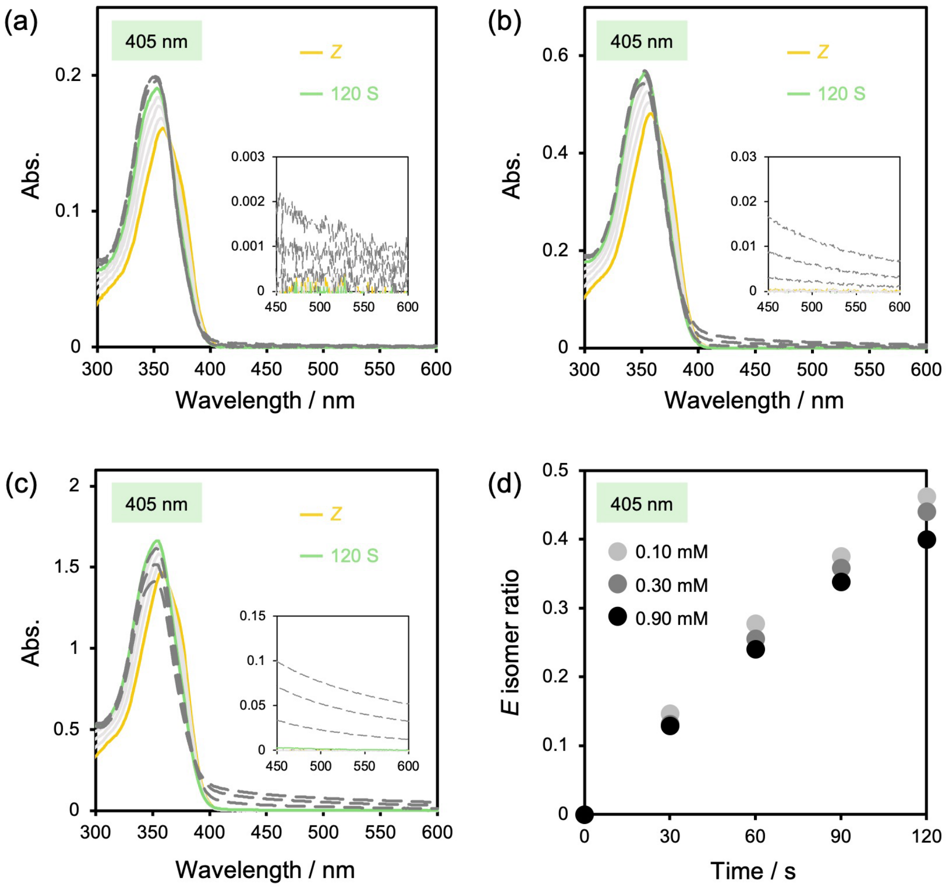

Fig. S31 UV/vis absorption spectra of ***Z*-P1** upon irradiation with 405 nm light in toluene at (a) 0.1 mM, (b) 0.3 mM, and (c) 0.9 mM. (d) Changes in *E*/*Z* ratios upon irradiation of ***Z*-P1** with 405 nm light in toluene.

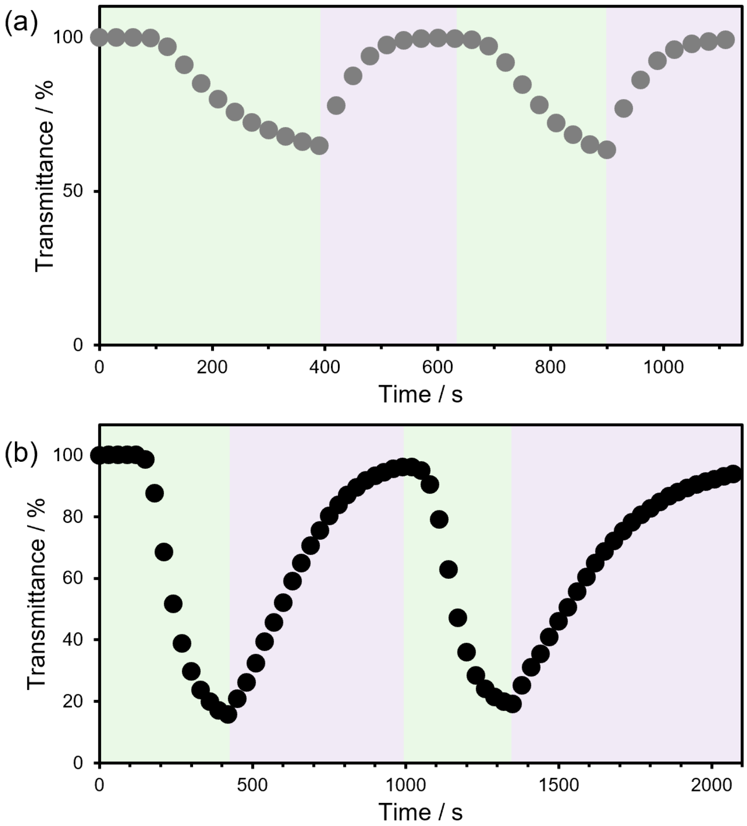

Fig. S32 Transmittance changes of toluene solutions of ***Z*-P1** at (a) 0.30 mM and (b) 0.90 mM during alternating irradiation with 405 and 300 nm light.

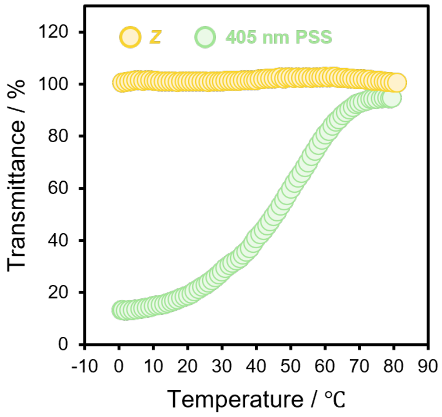

Fig. S33 Transmittance changes of toluene solutions (0.90 mM) of ***Z*-P1** and 405 nm PSS with ca. 80% *E* isomer during heating from 0 °C to 80 °C at a heating rate of 5 °C min^{-1}.

Thermal properties in bulk

TGA and DSC were employed to study thermal properties of the polymers in the bulk state. ^{1}H NMR and SEC measurements were employed to study thermal isomerization of the main-chain HSS photoswitches in the bulk state.

For DSC measurements, **P1** (M_n = 13200), **P2**, and **P3** with two different *E*/*Z* ratios were prepared by exposure of their 100% *E* polymers in THF (1.00 ×10^{-3} M) to 300 nm LED light (0.40 mW cm^{-2}, LDR2-100UV300, CCS) (Fig. S29) under stirring and drying in vacuum (Fig. S37–S39). DSC measurements were performed by heating three times from −50 °C to 200 °C for ***E*-P1** (M_n = 13200), ***Z*-P1**, ***E*-P2**, and ***E*-P3** (Fig. S35), from −50 °C to 90 °C for **P1** (M_n = 13200) with the different *E*/*Z* ratios, and from −50 °C to 80 °C for **P2** and **P3** with the different *E*/*Z* ratios at a heating rate of 10 °C min^{-1} under nitrogen atmosphere. Inflection points of the third scans were used to determine the T_gs (Fig. S40).

For thermal isomerization, ***E*-P1** (M_n = 13200), ***Z*-P1**, ***E*-P2**, and ***E*-P3** (1 mg) were placed in a glass vial and heated at 80, 100, 120, and 140 °C in vacuum for different periods, and ^{1}H NMR spectra of the resulting polymers were measured in $CDCl_3$ to determine the *E*/*Z* ratios.

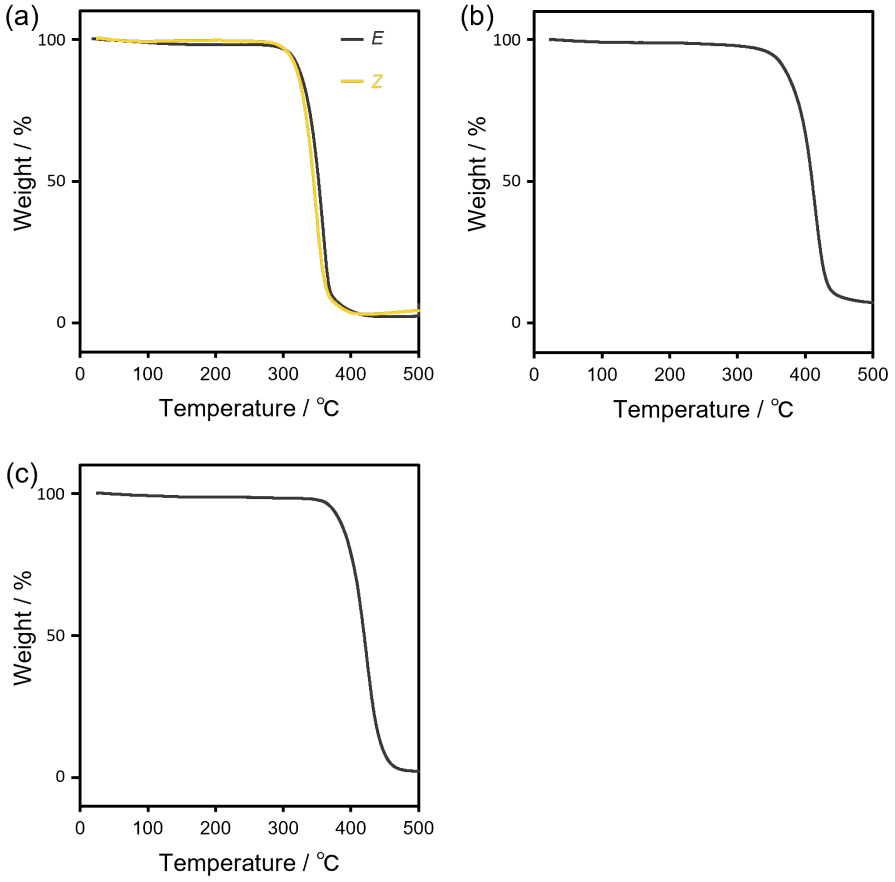

Fig. S34 TGA curves of (a) ***E*-P1** (M_n = 13200) and ***Z*-P1**, (b) ***E*-P2**, and (c) ***E*-P3**.

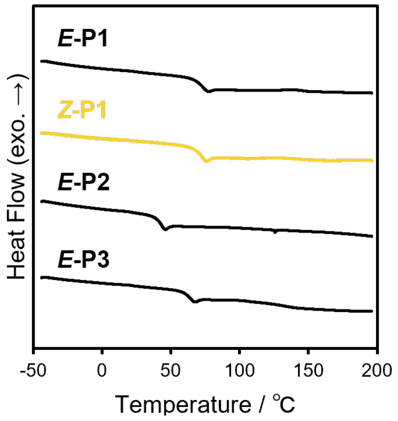

Fig. S35 DSC curves of ***E*-P1** (M_n = 13200), ***Z*-P1**, ***E*-P2**, and ***E*-P3**.

Table S2 Temperatures at 5% weight loss ($T_{5\%}$s) and T_gs of ***E*-P1** (M_n = 13200), ***Z*-P1**, ***E*-P2**, and ***E*-P3** in TGA and DSC

	***E*-P1**	***Z*-P1**	***E*-P2**	***E*-P3**
$T_{5\%}{}^{a}$ / °C	309	307	350	371
$T_g{}^{b}$ / °C	73	72	44	64

[a]Determined by TGA. [b]Inflection points in DSC curves.

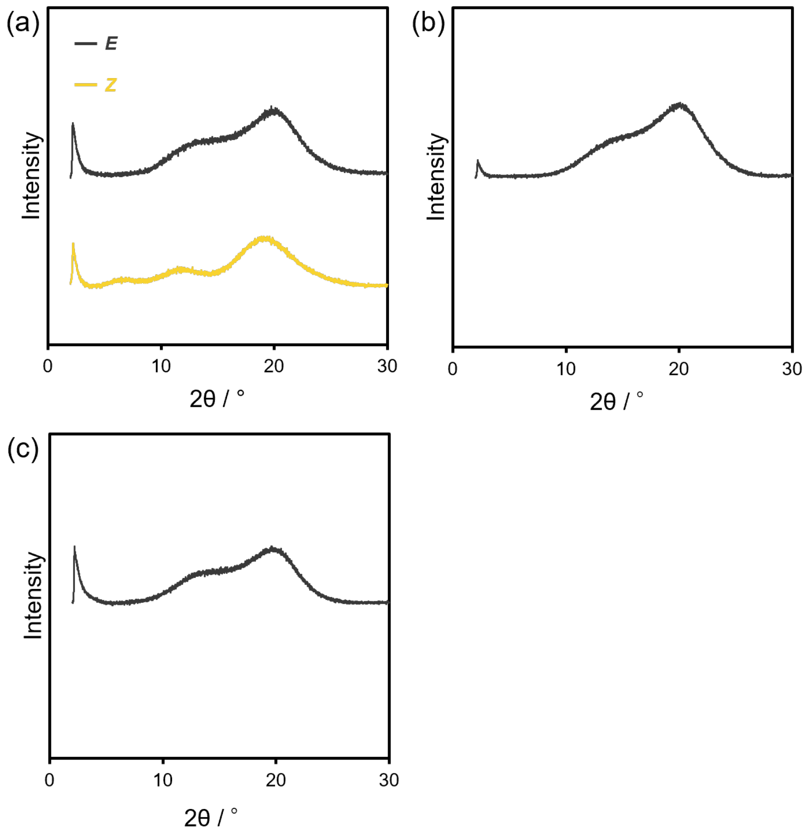

Fig. S36 XRD profiles of (a) ***E*-P1** (M_n = 13200) and ***Z*-P1**, (b) ***E*-P2**, and (c) ***E*-P3**.

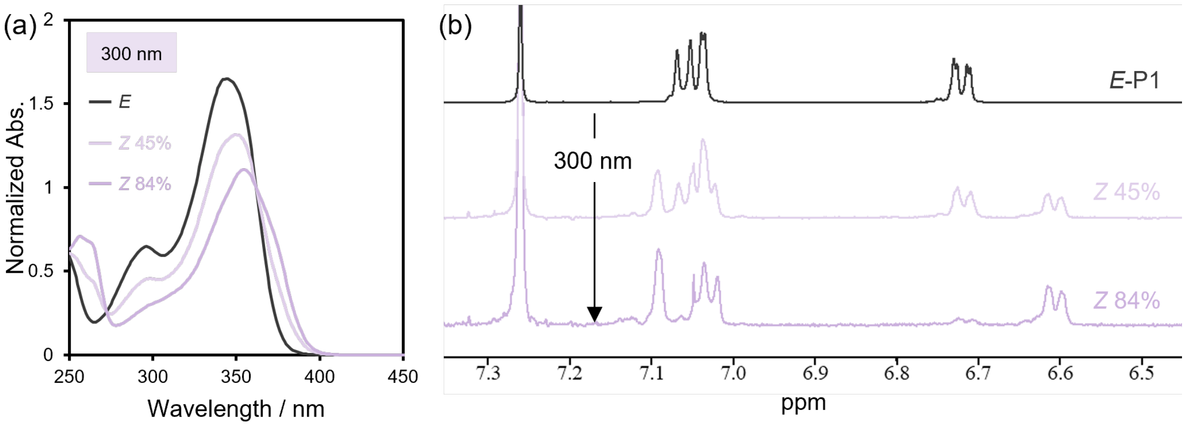

Fig. S37 (a) UV/vis absorption spectra and (b) ^{1}H NMR spectra (500 MHz, $CDCl_3$) of **P1** (M_n = 13200) with different *E*/*Z* ratios prepared by exposure of ***E*-P1** (M_n = 13200) in THF (1.00 ×10^{-3} M) to 300 nm light. UV/vis absorption spectra were measured in THF (1.00 ×10^{-3} M) and normalized at 362 nm.

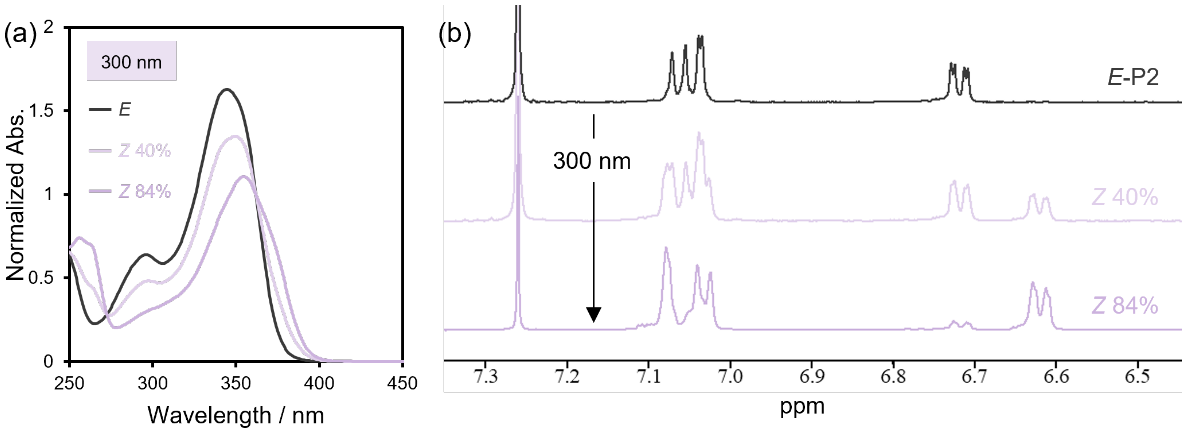

Fig. S38 (a) UV/vis absorption spectra and (b) ^{1}H NMR spectra (500 MHz, $CDCl_3$) of **P2** with different *E*/*Z* ratios prepared by exposure of ***E*-P2** in THF (1.00 ×10^{-3} M) to 300 nm light. UV/vis absorption spectra were measured in THF (1.00 ×10^{-3} M) and normalized at 362 nm.

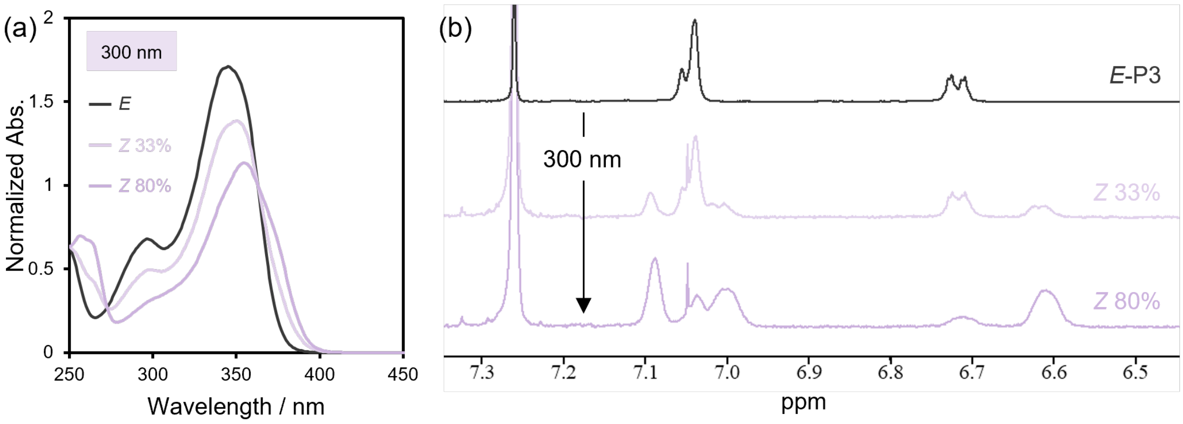

Fig. S39 (a) UV/vis absorption spectra and (b) ^{1}H NMR spectra (500 MHz, $CDCl_3$) of **P3** with different *E*/*Z* ratios prepared by exposure of ***E*-P3** in THF (1.00×10^{-3} M) to 300 nm light. UV/vis absorption spectra were measured in THF (1.00×10^{-3} M) and normalized at 363 nm.

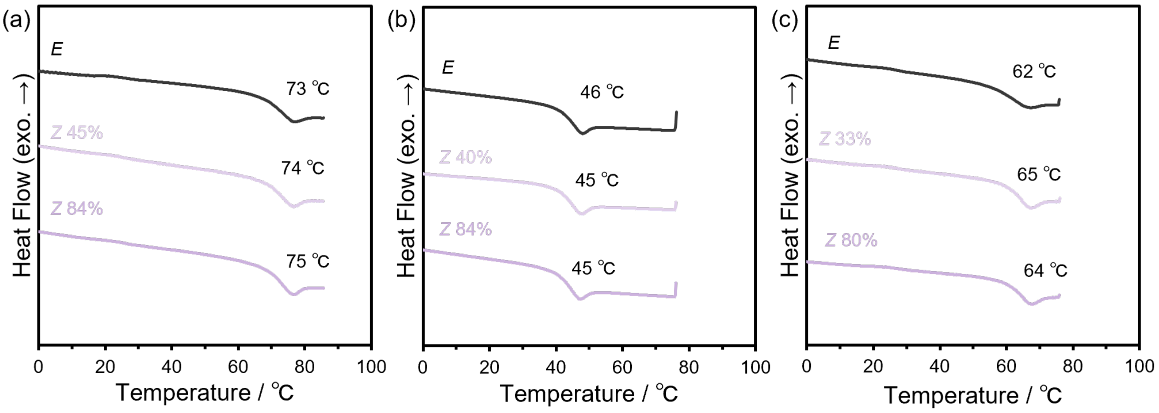

Fig. S40 DSC curves and T_gs of (a) **P1** (M_n = 13200), (b) **P2**, and (c) **P3** with different *E*/*Z* ratios.

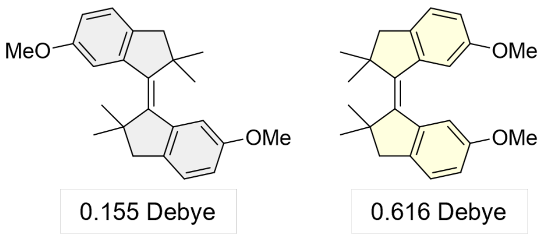

Fig. S41 Dipole moments of *E* and *Z* isomers of HSS with two methoxy groups at 298.15 K. Dipole moments were calculated according to our previously published methods.[3]

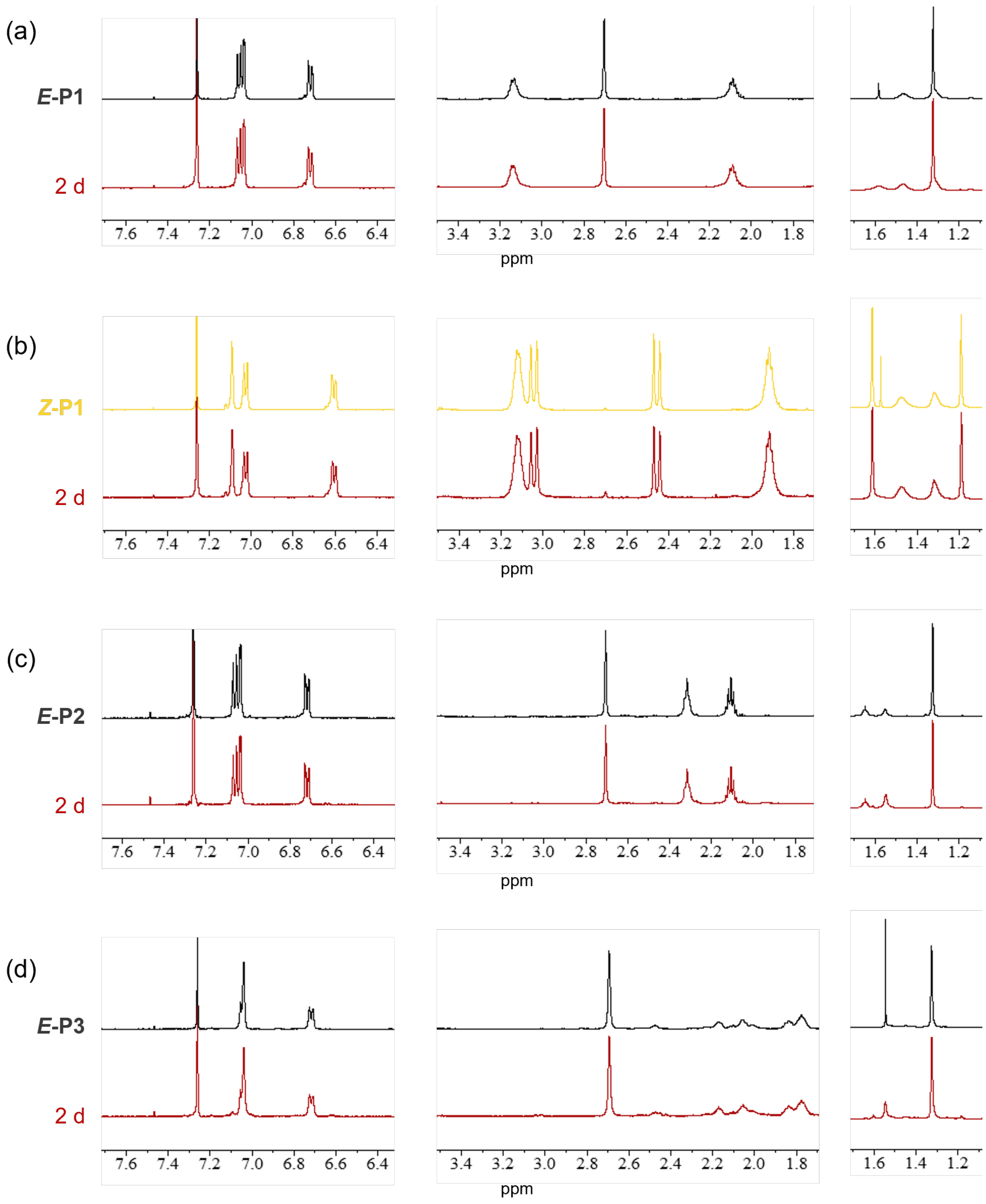

Fig. S42 ^{1}H NMR spectra (500 MHz, $CDCl_3$) of (a) ***E*-P1** (M_n = 13200), (b) ***Z*-P1**, (c) ***E*-P2**, and (d) ***E*-P3** before and after heating at 80 °C in vacuum for 2 days in bulk.

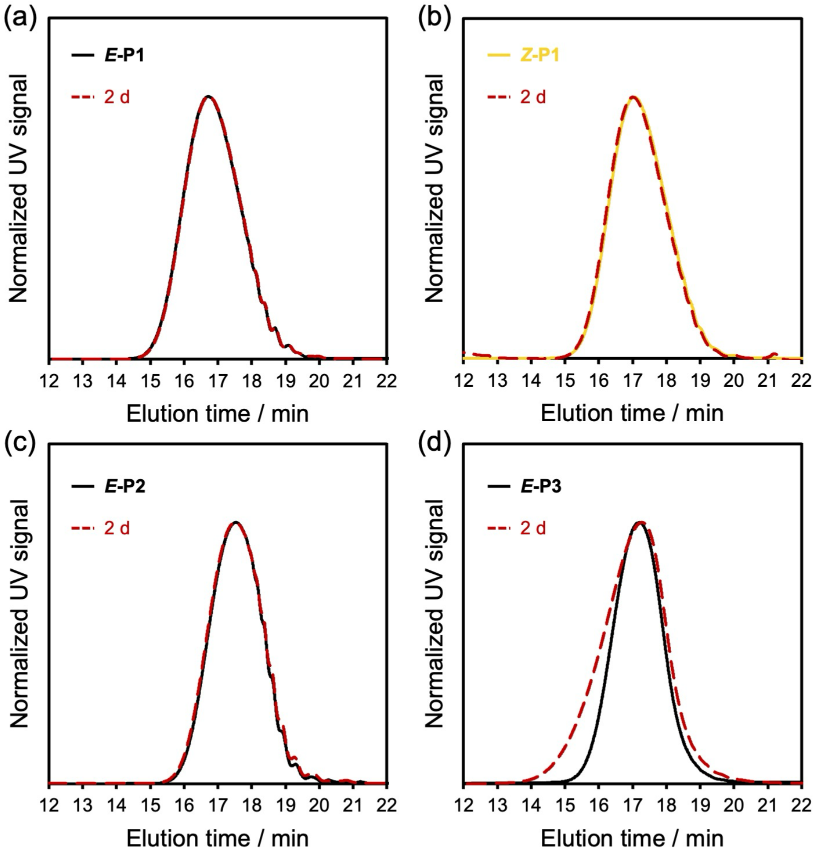

Fig. S43 SEC curves of (a) ***E*-P1** (M_n = 13200), (b) ***Z*-P1**, (c) ***E*-P2**, and (d) ***E*-P3** before and after heating at 80 °C in vacuum for 2 days in bulk.

Table S3 M_ns and M_w/M_ns of ***E*-P1** (M_n = 13200), ***Z*-P1**, ***E*-P2**, and ***E*-P3** before and after heating at 80 °C in vacuum for 2 days in bulk

		***E*-P1**	***Z*-P1**	***E*-P2**	***E*-P3**
Initial	M_n	13200	9900	8000	11900
	M_w/M_n	1.61	1.59	1.44	1.36
2 d	M_n	12900	10200	7900	10600
	M_w/M_n	1.62	1.59	1.48	2.10

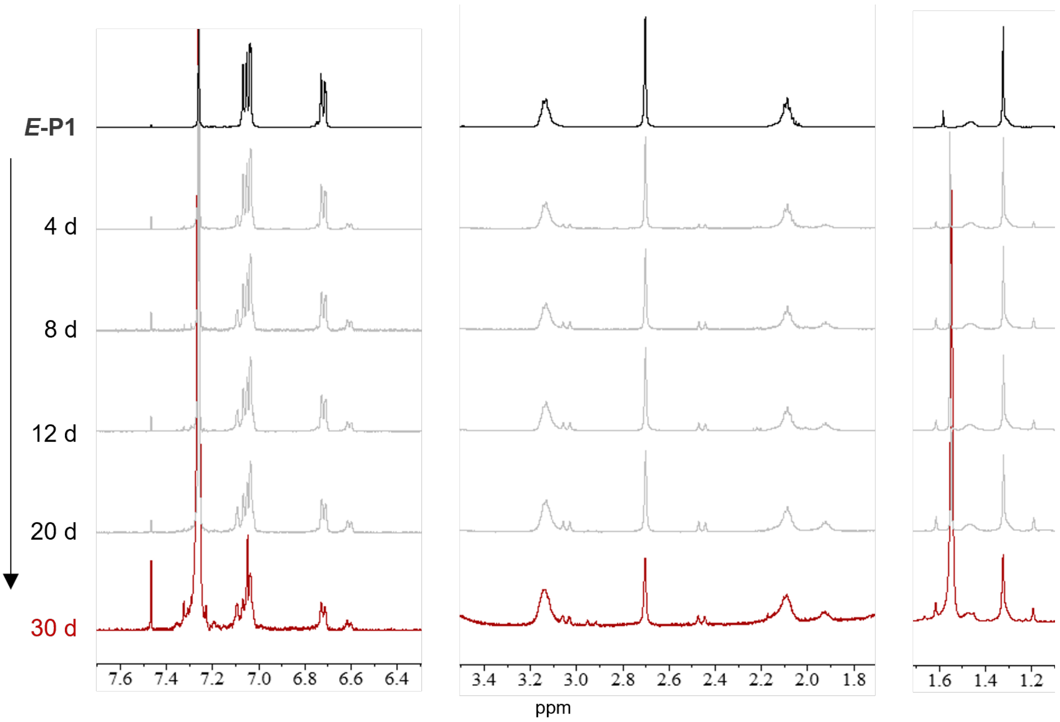

Fig. S44 Changes in ^{1}H NMR spectra (500 MHz, $CDCl_3$) of ***E*-P1** (M_n = 13200) upon heating at 100 °C in vacuum in bulk.

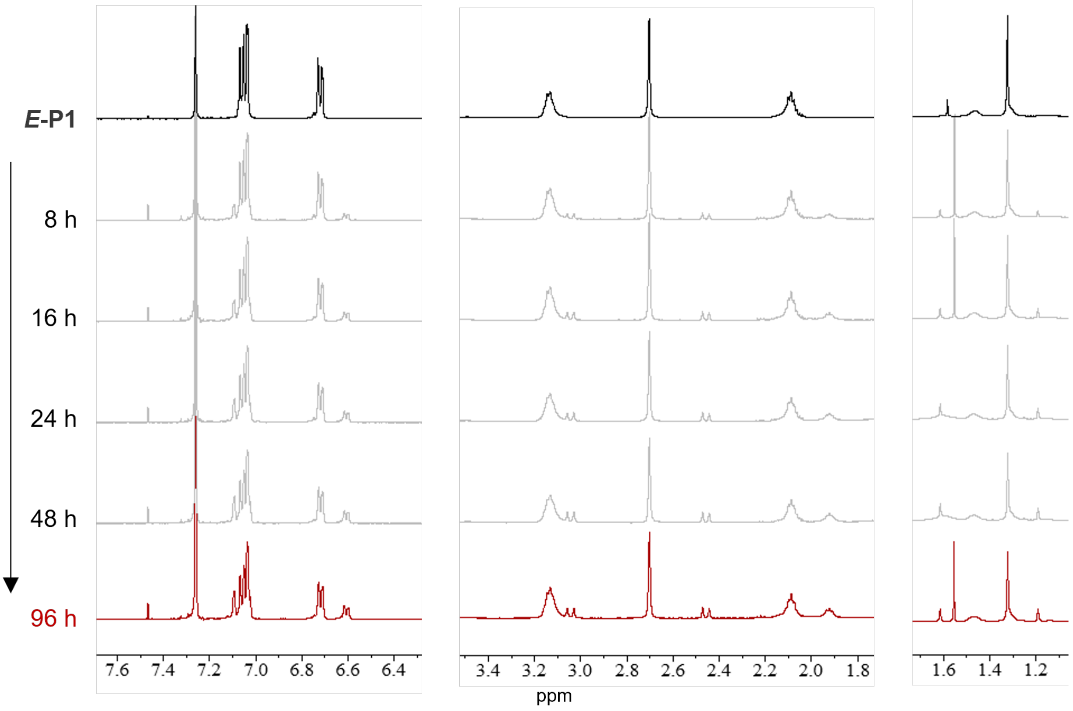

Fig. S45 Changes in ^{1}H NMR spectra (500 MHz, $CDCl_3$) of ***E*-P1** (M_n = 13200) upon heating at 120 °C in vacuum in bulk.

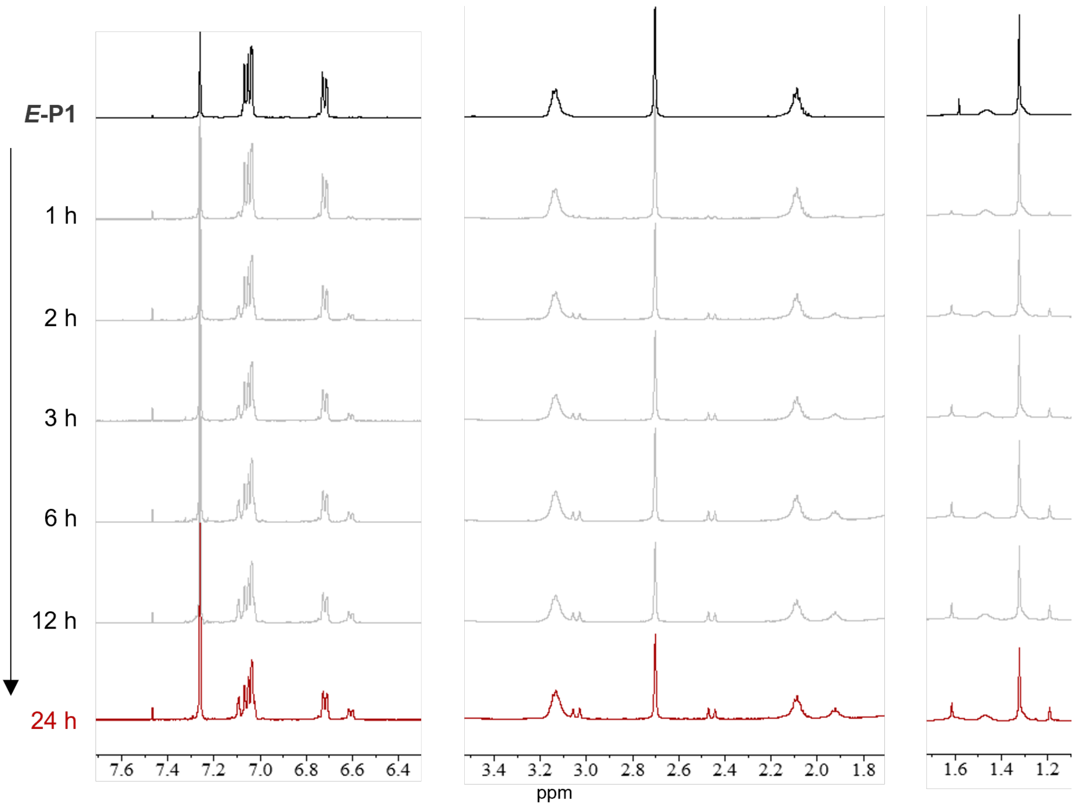

Fig. S46 Changes in ^{1}H NMR spectra (500 MHz, $CDCl_3$) of ***E*-P1** (M_n = 13200) upon heating at 140 °C in vacuum in bulk.

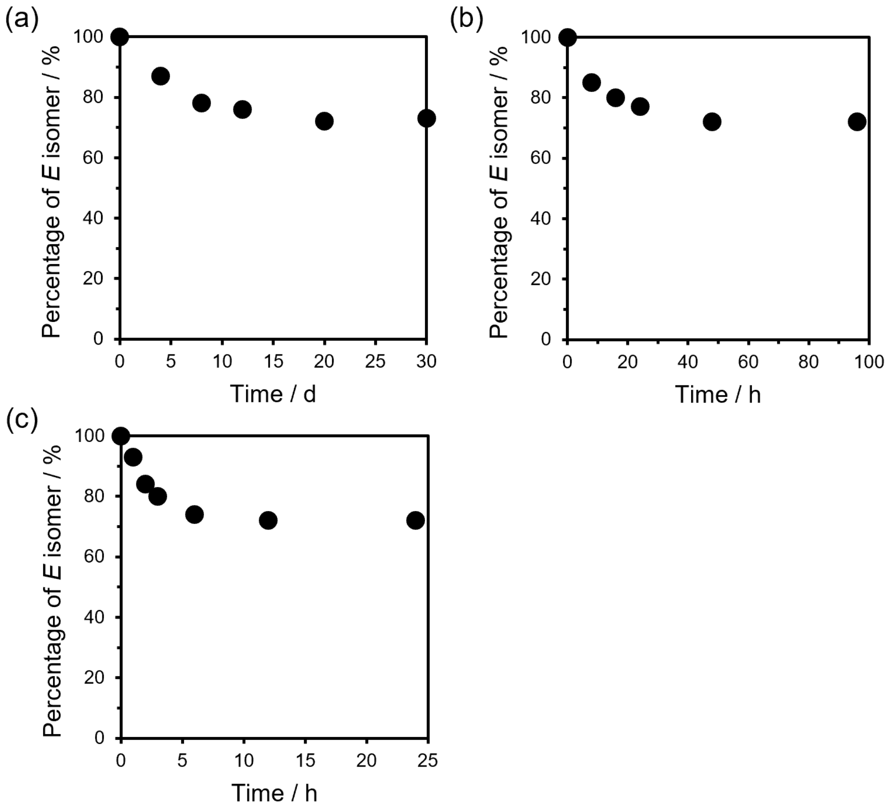

Fig. S47 Changes in E/Z ratios of ***E*-P1** (M_n = 13200) upon heating at (a) 100 °C, (b) 120 °C, and (c) 140 °C in vacuum in bulk.

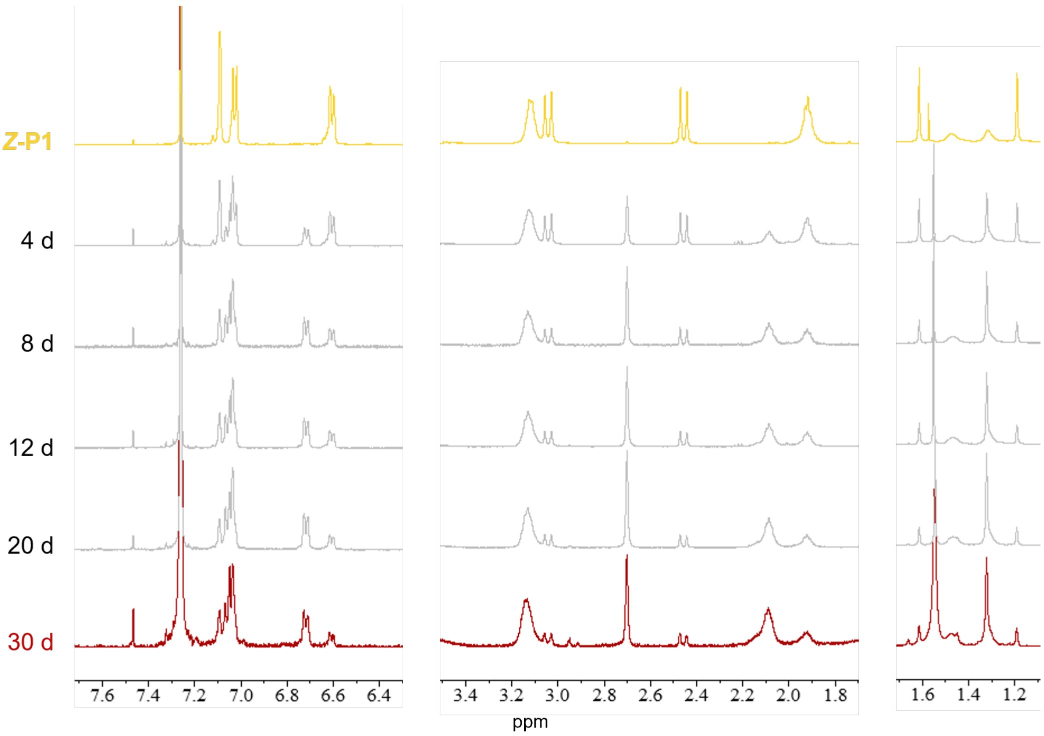

Fig. S48 Changes in ^{1}H NMR spectra (500 MHz, $CDCl_3$) of ***Z*-P1** upon heating at 100 °C in vacuum in bulk.

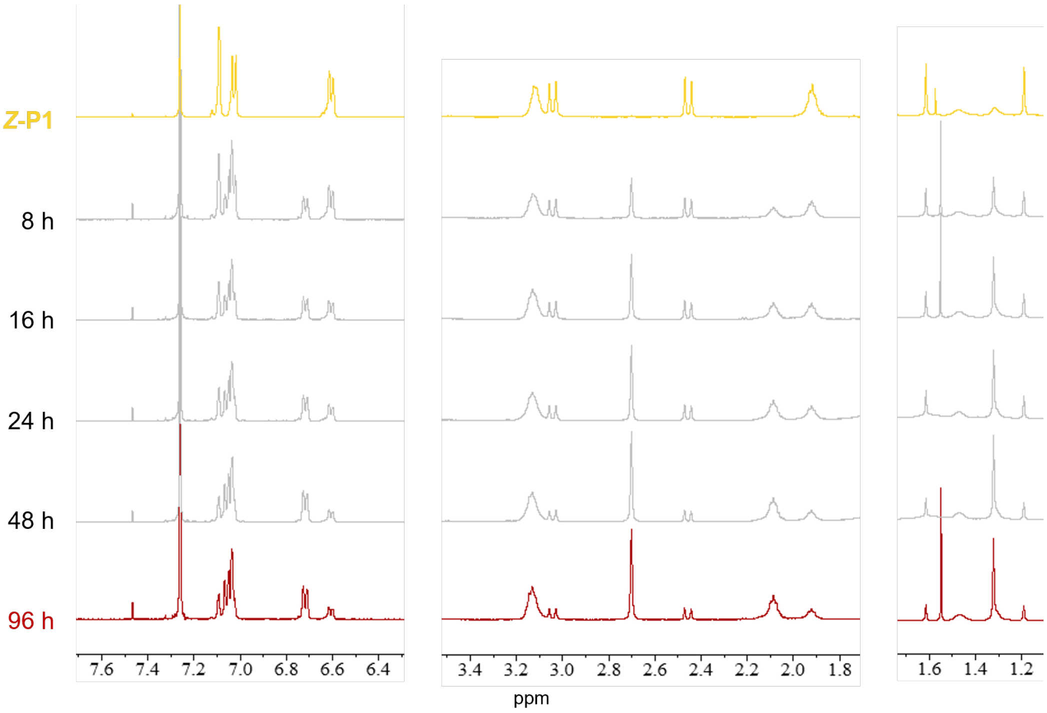

Fig. S49 Changes in ^{1}H NMR spectra (500 MHz, $CDCl_3$) of ***Z*-P1** upon heating at 120 °C in vacuum in bulk.

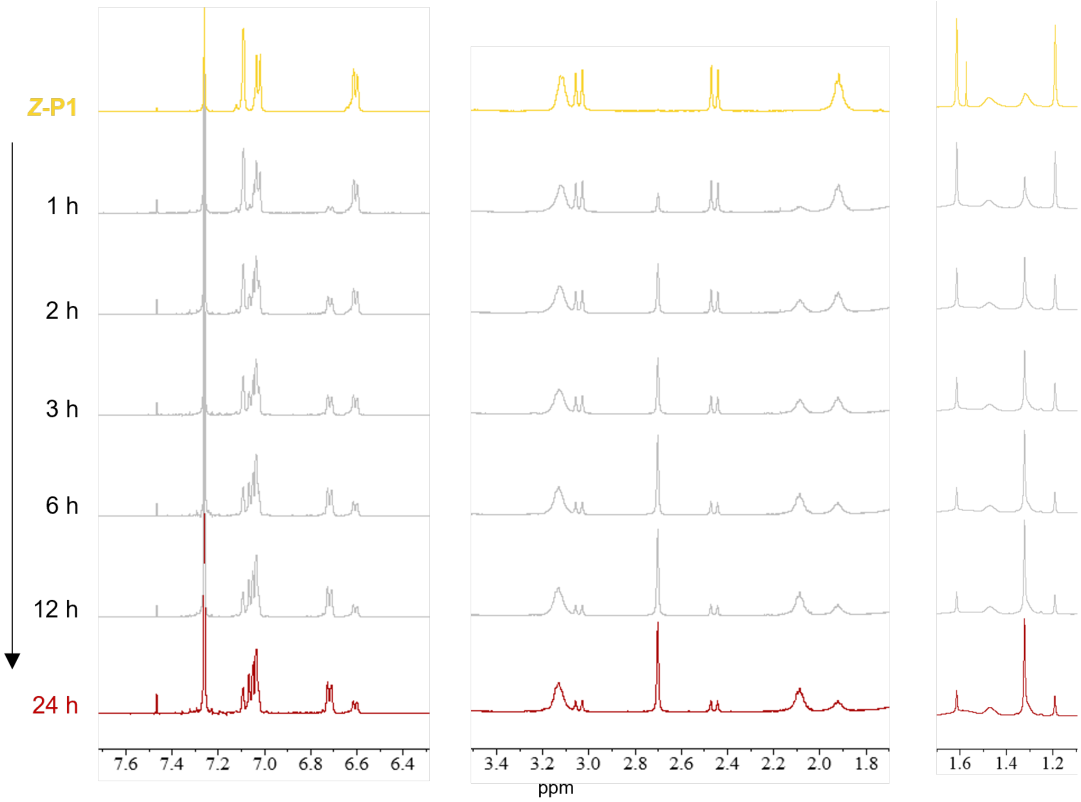

Fig. S50 Changes in ^{1}H NMR spectra (500 MHz, $CDCl_3$) of ***Z*-P1** upon heating at 140 °C in vacuum in bulk.

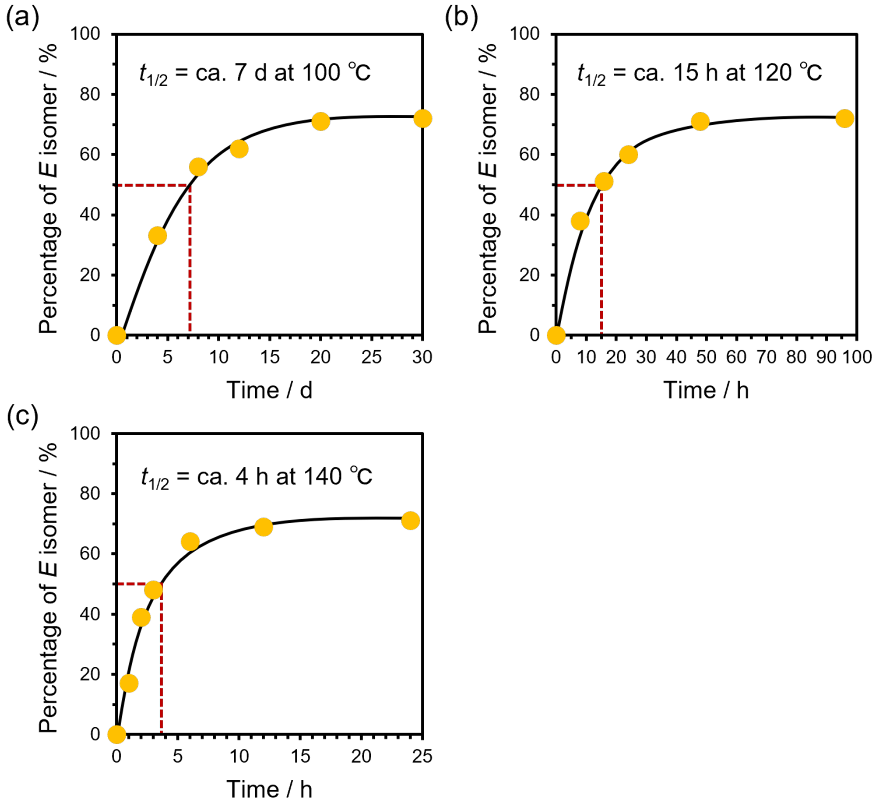

Fig. S51 Changes in *E*/*Z* ratios of ***Z*-P1** upon heating at (a) 100 °C, (b) 120 °C, and (c) 140 °C in vacuum in bulk and half-lives ($t_{1/2}$s) of *Z*-to-*E* thermal isomerization.

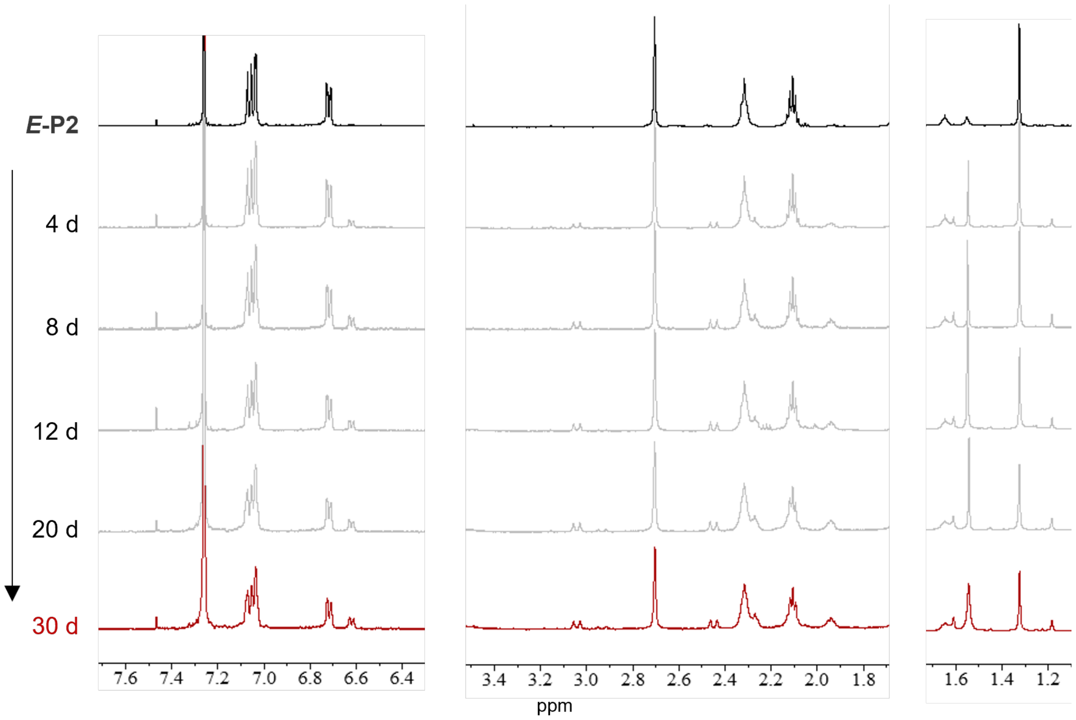

Fig. S52 Changes in ^{1}H NMR spectra (500 MHz, $CDCl_3$) of ***E*-P2** upon heating at 100 °C in vacuum in bulk.

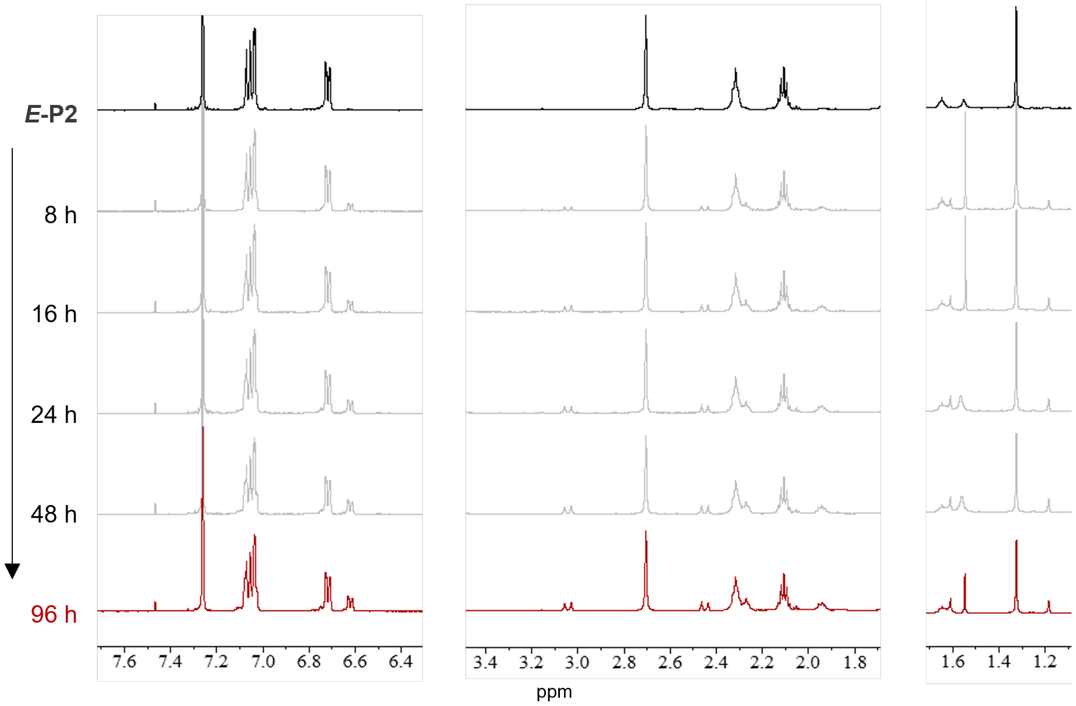

Fig. S53 Changes in ^{1}H NMR spectra (500 MHz, $CDCl_3$) of ***E*-P2** upon heating at 120 °C in vacuum in bulk.

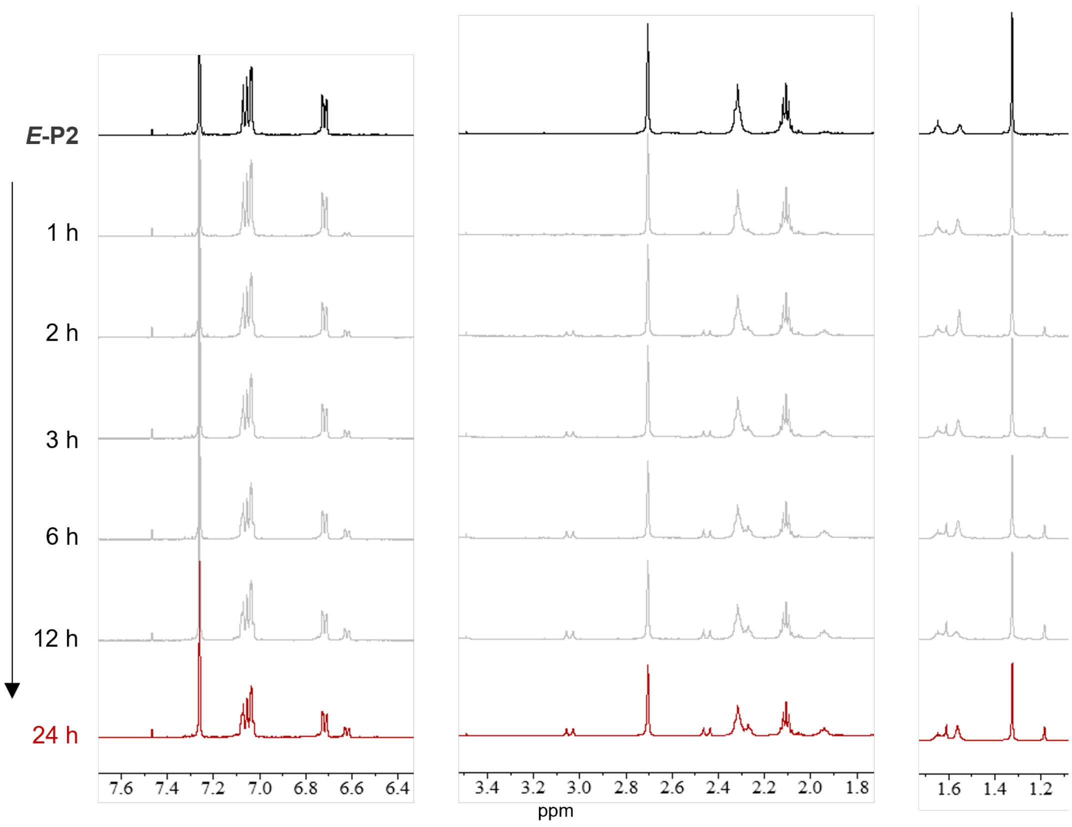

Fig. S54 Changes in ^{1}H NMR spectra (500 MHz, $CDCl_3$) of ***E*-P2** upon heating at 140 °C in vacuum in bulk.

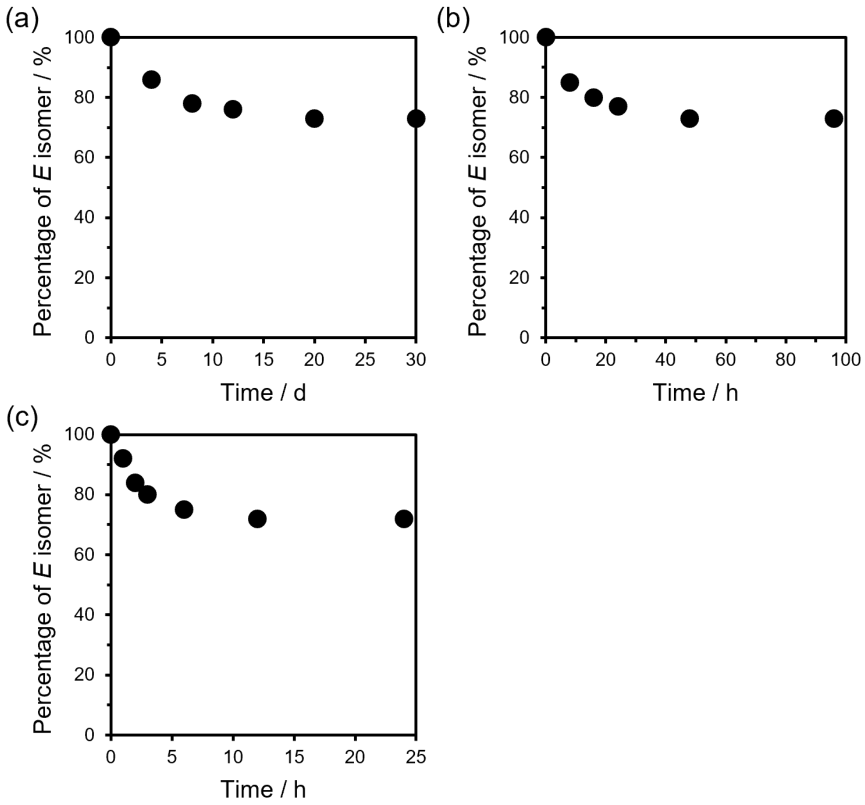

Fig. S55 Changes in *E*/*Z* ratios of ***E*-P2** upon heating at (a) 100 °C, (b) 120 °C, and (c) 140 °C in vacuum in bulk.

Photoisomerization in the glassy state

Polymer thin films were prepared by spin coating on hydrophobic quartz substrates. UV/vis absorption and ^{1}H NMR spectroscopy was employed to study photoisomerization of the main-chain HSS photoswitches in the thin films.

Quartz substrates (20 × 20 × 0.8–1.0 mm) were treated with piranha solution to clean the surfaces and expose hydroxy groups, washed with water, and dried in vacuum at RT. Then, the hydrophilic substrates were treated with 1,1,1,3,3,3-hexamethyldisilazane (HMDS) at 140 °C for 15 min to modify a hydrophobic monolayer, washed by sonication in acetone for 15 min, and dried in vacuum at RT. Thin films of ***E*-P1** (M_n = 13200), ***Z*-P1**, ***E*-P2**, and ***E*-P3** were prepared by spin coating of the DCM solutions (160 μL, 5 or 10 mg mL^{-1}) on the HMDS-treated substrates under nitrogen atmosphere at 1000 rpm for 30 s using a Mikasa MS-A100 Opticoat spin coater and drying in vacuum at RT overnight. *Z*-rich **P2** (84% *Z*) and **P3** (80% *Z*) were obtained by exposure of ***E*-P2** and ***E*-P3** in THF (1.00 ×10^{-3} M) to 300 nm LED light (0.40 mW cm^{-2}, LDR2-100UV300, CCS) (Fig. S29) under stirring and drying in vacuum (Fig. S38 and S39), and their thin films were also prepared in the same procedure. Prior to spin coating, the polymer solutions were stirred in the dark at RT overnight. The polymer thin films were irradiated with 300 nm LED light (0.40 mW cm^{-2}, LDR2-100UV300, CCS) and 405 nm LED light (19.6 mW cm^{-2}, LDR2-100VL405-W1U, CCS) (Fig. S29). The exposure distance was 40 mm for the 300 nm light and 80 mm for the 405 nm light. The *E*/*Z* ratios were determined from ^{1}H NMR spectra.

Table S4 Thickness of ***E*-P1** (M_n = 13200), ***Z*-P1**, ***E*-P2**, and ***E*-P3** thin films

Thin film	Concentration (mg mL^{-1})	Thickness (nm)^{a}
***E*-P1**	10	137 ± 13
***E*-P1**	5	72 ± 12
***Z*-P1**	10	134 ± 15
***E*-P2**	10	176 ± 17
***E*-P3**	10	180 ± 8

^{a}Averages and standard deviations of three measurements at different positions in thin films.

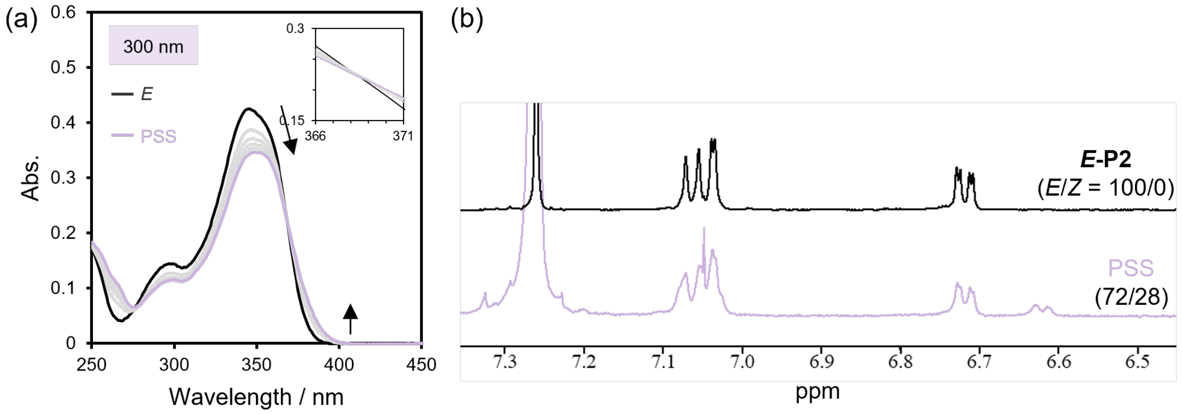

Fig. S56 (a) UV/vis absorption spectra and (b) ^{1}H NMR spectra (500 MHz, $CDCl_3$) of ***E*-P2** thin film upon irradiation with 300 nm light. The film was prepared by spin coating of the DCM solution (10 mg mL^{-1}).

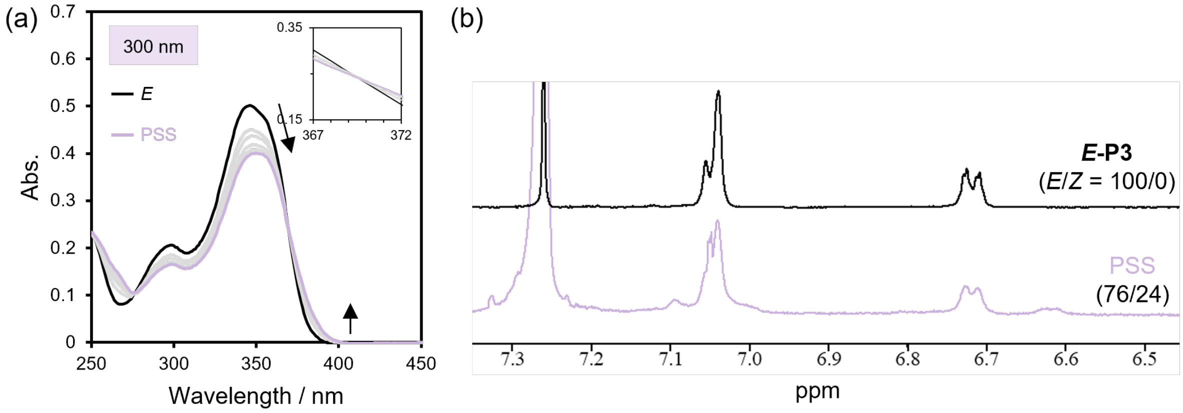

Fig. S57 (a) UV/vis absorption spectra and (b) ^{1}H NMR spectra (500 MHz, $CDCl_3$) of ***E*-P3** thin film upon irradiation with 300 nm light. The film was prepared by spin coating of the DCM solution (10 mg mL^{-1}).

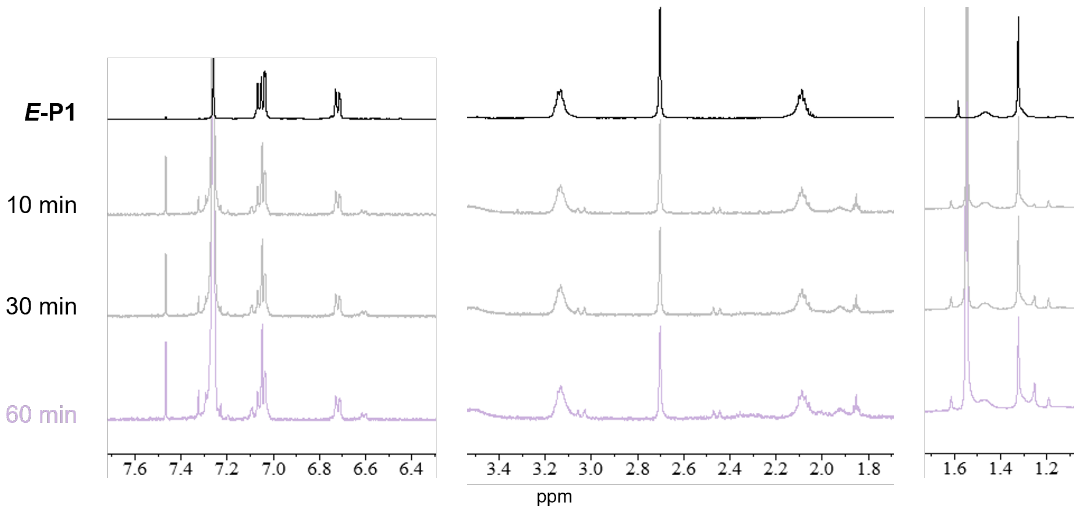

Fig. S58 Changes in ^{1}H NMR spectra (500 MHz, $CDCl_3$) of ***E*-P1** (M_n = 13200) thin film upon irradiation with 300 nm light. The film was prepared by spin coating of the DCM solution (10 mg mL^{-1}).

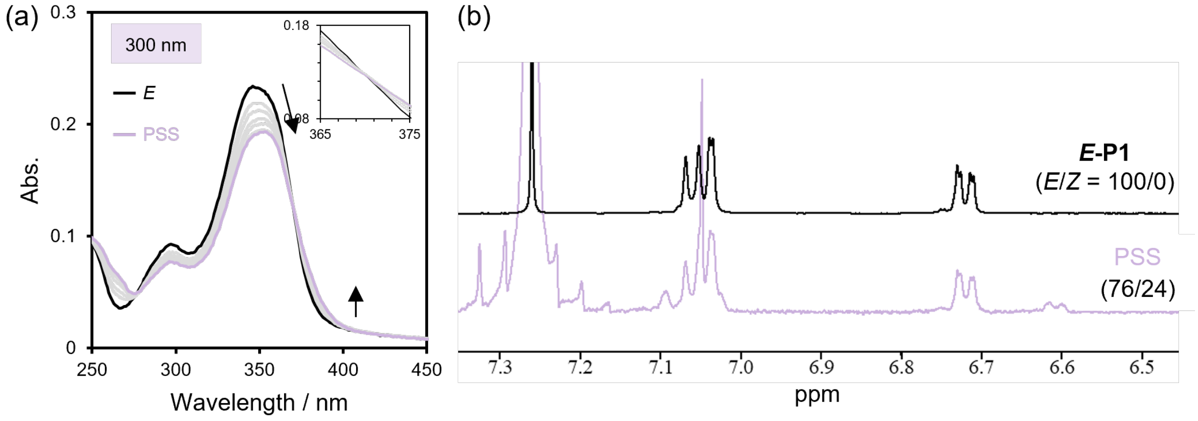

Fig. S59 (a) UV/vis absorption spectra and (b) ^{1}H NMR spectra (500 MHz, $CDCl_3$) of ***E*-P1** (M_n = 13200) thinner film upon irradiation with 300 nm light. The film was prepared by spin coating of the DCM solution (5 mg mL^{-1}).

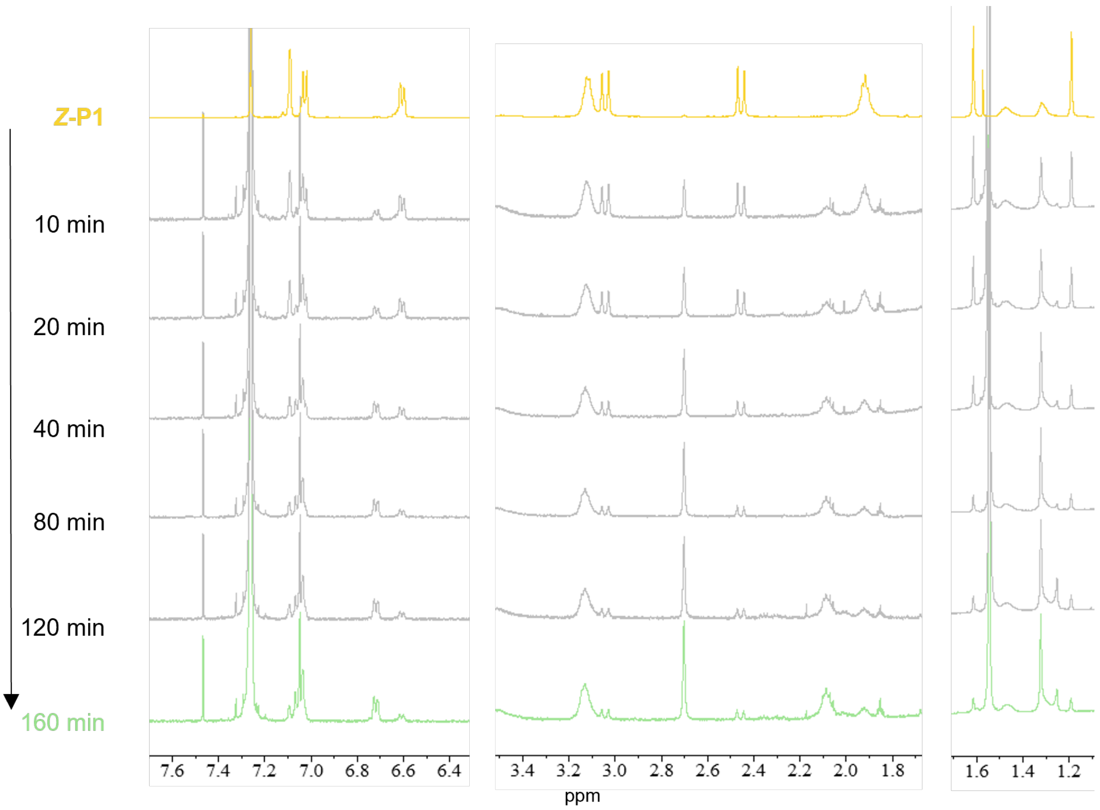

Fig. S60 Changes in ^{1}H NMR spectra (500 MHz, $CDCl_3$) of ***Z*-P1** thin film upon irradiation with 405 nm light. The film was prepared by spin coating of the DCM solution (10 mg mL^{-1}).

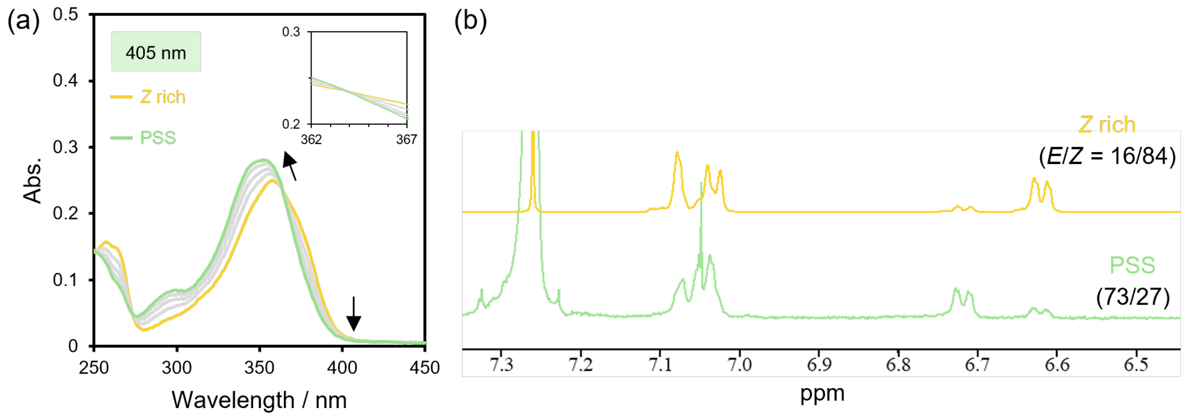

Fig. S61 (a) UV/vis absorption spectra and (b) ^{1}H NMR spectra (500 MHz, $CDCl_3$) of *Z*-rich **P2** thin film upon irradiation with 405 nm light. The film was prepared by spin coating of the DCM solution (10 mg mL^{-1}).

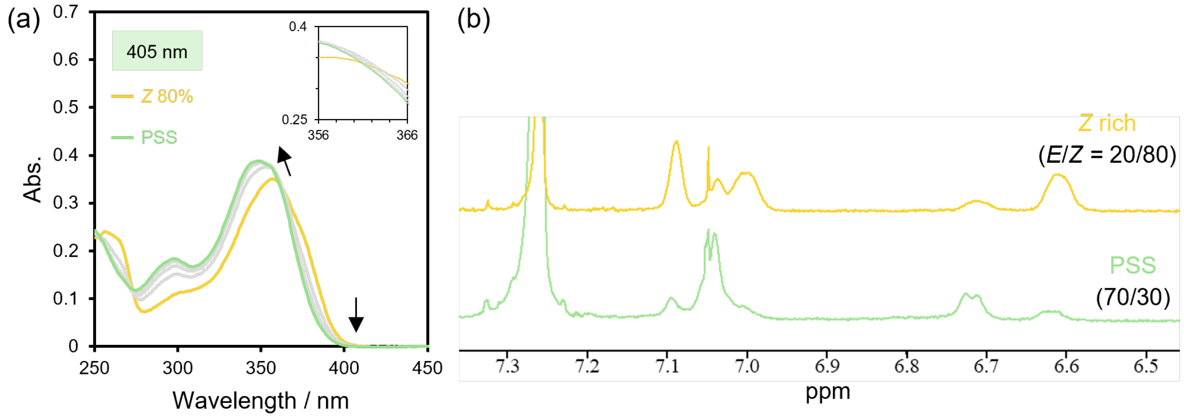

Fig. S62 (a) UV/vis absorption spectra and (b) ^{1}H NMR spectra (500 MHz, $CDCl_3$) of *Z*-rich **P3** thin film upon irradiation with 405 nm light. The film was prepared by spin coating of the DCM solution (10 mg mL^{-1}).

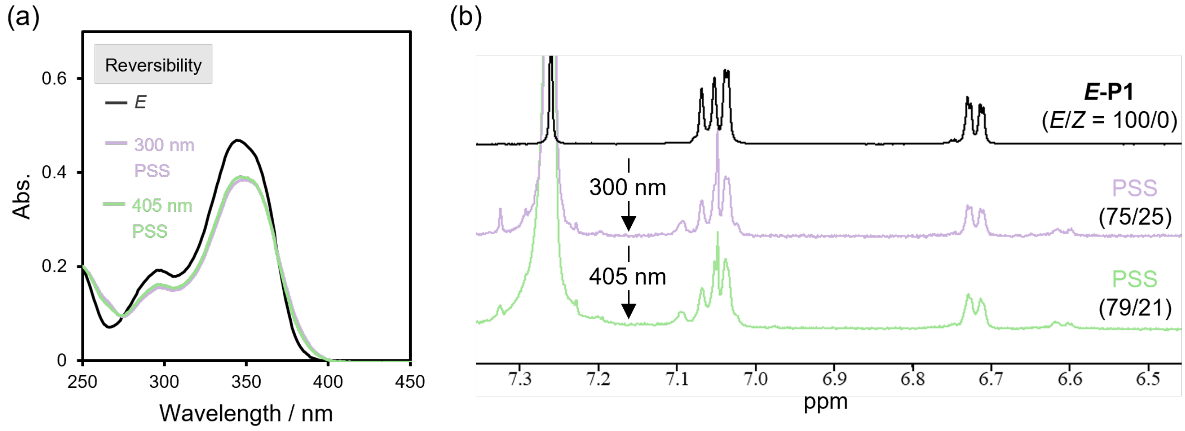

Fig. S63 (a) UV/vis absorption spectra and (b) ^{1}H NMR spectra (500 MHz, $CDCl_3$) of ***E*-P1** (M_n = 13200) thin film upon irradiation with 300 nm light and subsequent 405 nm light. The film was prepared by spin coating of the DCM solution (10 mg mL^{-1}).

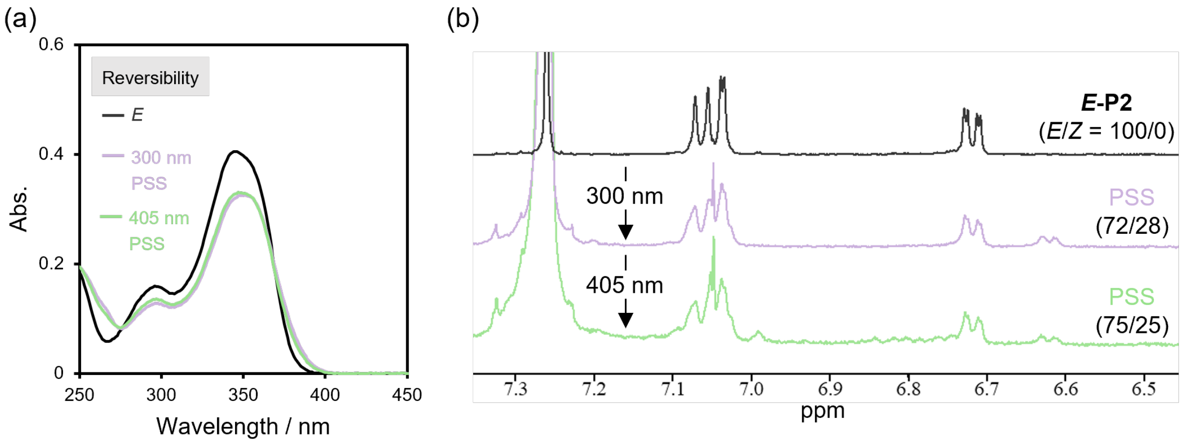

Fig. S64 (a) UV/vis absorption spectra and (b) ^{1}H NMR spectra (500 MHz, $CDCl_3$) of ***E*-P2** thin film upon irradiation with 300 nm light and subsequent 405 nm light. The film was prepared by spin coating of the DCM solution (10 mg mL^{-1}).

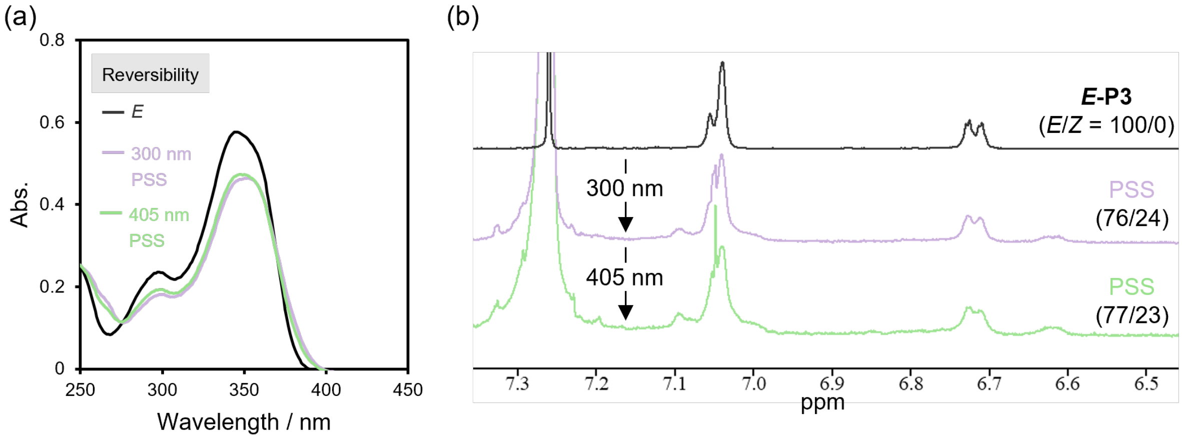

Fig. S65 (a) UV/vis absorption spectra and (b) ^{1}H NMR spectra (500 MHz, $CDCl_3$) of ***E*-P3** thin film upon irradiation with 300 nm light and subsequent 405 nm light. The film was prepared by spin coating of the DCM solution (10 mg mL^{-1}).

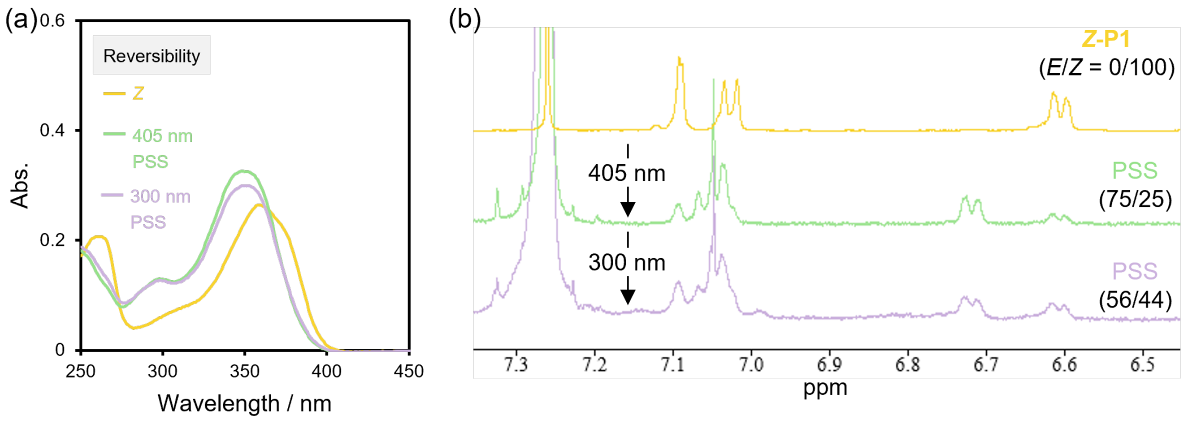

Fig. S66 (a) UV/vis absorption spectra and (b) ^{1}H NMR spectra (500 MHz, $CDCl_3$) of ***Z*-P1** thin film upon irradiation with 405 nm light and subsequent 300 nm light. The film was prepared by spin coating of the DCM solution (10 mg mL^{-1}).

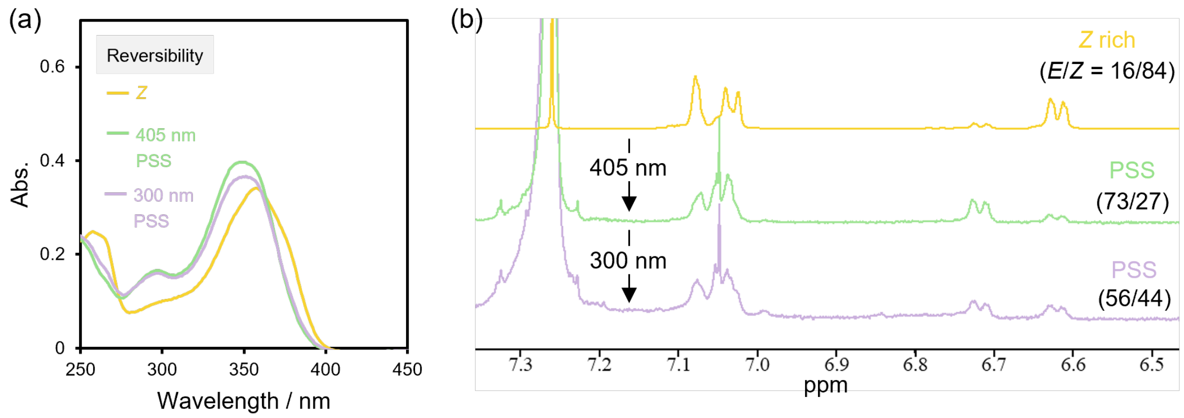

Fig. S67 (a) UV/vis absorption spectra and (b) ^{1}H NMR spectra (500 MHz, $CDCl_3$) of *Z*-rich **P2** thin film upon irradiation with 405 nm light and subsequent 300 nm light. The film was prepared by spin coating of the DCM solution (10 mg mL^{-1}).

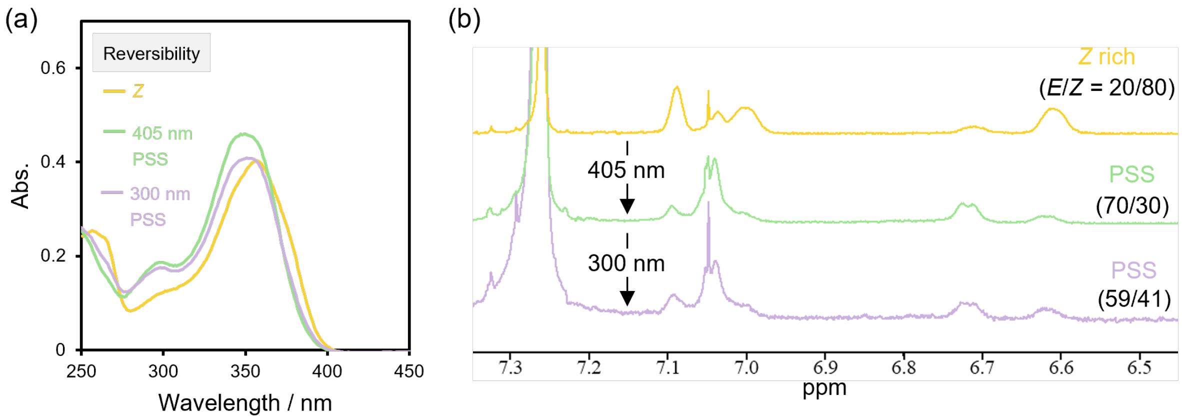

Fig. S68 (a) UV/vis absorption spectra and (b) ^{1}H NMR spectra (500 MHz, $CDCl_3$) of *Z*-rich **P3** thin film upon irradiation with 405 nm light and subsequent 300 nm light. The film was prepared by spin coating of the DCM solution (10 mg mL^{-1}).

Wettability of thin film surfaces

Static and dynamic contact angle measurements were employed to evaluate wettability of the polymer film surfaces. Thin films of **P1**, **P2**, and **P3** with different *E*/*Z* ratios were prepared by spin coating of the DCM solutions (160 μL, 10 mg mL^{-1}) of the polymers with different *E*/*Z* ratios on the HMDS-treated quartz substrates (20 × 20 × 0.8–1.0 mm) under nitrogen atmosphere at 1000 rpm for 30 s and drying in vacuum at RT overnight. The DCM solutions of **P1** with different *E*/*Z* ratios were obtained by exposure of ***Z*-P1** in THF (1.00 $\times 10^{-3}$ M) to 405 nm LED light (19.6 mW cm^{-2}, LDR2-100VL405-W1U, CCS) (Fig. S29) under stirring and drying in vacuum (Fig. S69). The DCM solutions of **P2** and **P3** with different *E*/*Z* ratios were obtained by exposure of ***E*-P2** and ***E*-P3** in THF (1.00 $\times 10^{-3}$ M) to 300 nm LED light (0.40 mW cm^{-2}, LDR2-100UV300, CCS) (Fig. S29) under stirring and drying in vacuum (Fig. S38 and S39). Thin films of **P1** with different *E*/*Z* ratios were also prepared by irradiation of the ***Z*-P1** thin film with 405 nm LED light (19.6 mW cm^{-2}, LDR2-100VL405-W1U, CCS) (Fig. S29 and S72 and Table S8). Prior to spin coating, the polymer solutions were stirred in the dark at RT overnight. Static contact angles (θ_Ss) were measured by gently placing a drop of water (2 μL) or ethylene glycol (1 μL) at four different positions on each thin film surface (n = 4). Hysteresis of water contact angles was determined by the difference between advancing contact angles (θ_As) and receding contact angles (θ_Rs) at three different positions on each thin film surface (n = 3). The θ_As were measured when the contact area increased by slowly injecting water from a needle into a water droplet (5 μL) on the surfaces. The θ_Rs were measured when the contact area decreased by slowly withdrawing water from an extended droplet. Surface free energies (γ) and the dispersion (γ^d) and polar (γ^p) components of the thin films were calculated from the θ_S using eqs 1 and 2:

$$(1 + \cos\theta_S) \times \gamma_L/2 = \sqrt{\gamma^d \times \gamma_L^d} \times \sqrt{\gamma^p \times \gamma_L^p} \quad (1)$$

$$\gamma = \gamma^d + \gamma^p \quad (2)$$

where γ_L is the surface free energy of the liquid, and $\gamma_L{}^d$ and $\gamma_L{}^p$ are the dispersion and polar components of the liquid, respectively. The $\gamma_L{}^d$ and $\gamma_L{}^p$ values of water are 21.8 and 51.0 mJ m^{-2}, and those of ethylene glycol are 29.3 and 19.0 mJ m^{-2}, respectively.[4]

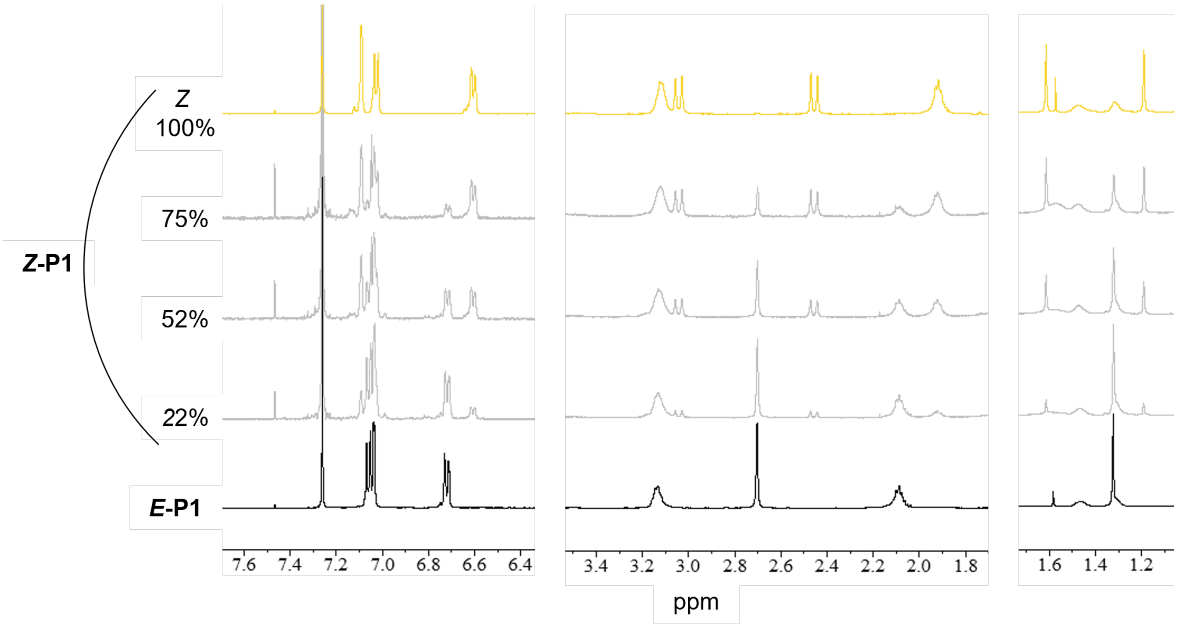

Fig. S69 ^{1}H NMR spectra (500 MHz, $CDCl_3$) of **P1** with different *E*/*Z* ratios.

Table S5 Root mean square (RMS) roughness (R_{rms}) estimated from AFM images of **P1**, **P2**, and **P3** thin films with different *E*/*Z* ratios (1 × 1 μm^2)

polymer	*Z* ratio (%)	R_{rms} (nm)[a]
**P1**[b]	0	0.32 ± 0.07
	100	0.66 ± 0.21
**P2**	0	1.14 ± 0.41
	84	0.71 ± 0.31
**P3**	0	0.91 ± 0.35
	80	0.64 ± 0.32

[a]Averages and standard deviations of four measurements at different positions on thin film surfaces.

[b]***E*-P1** (M_n = 13200) for 100% *E* **P1** and ***Z*-P1** for 100% *Z* **P1**.

Table S6 Static contact angles (θ_S) of water and ethylene glycol on **P1**, **P2**, and **P3** thin films with different *E*/*Z* ratios (n = 4, mean ± standard deviation) and surface free energies

polymer	*Z* ratio (%)	θ_S Water (deg)	θ_S ethylene glycol (deg)	Surface free energy γ^d (mJ m^{-2})	Surface free energy γ^p (mJ m^{-2})	Surface free energy γ (mJ m^{-2})
**P1**[a]	0	79.2 ± 0.3	55.2 ± 0.1	20.3	9.6	29.9
	22	85.1 ± 0.5	60.5 ± 0.5	21.7	6.2	27.9
	52	88.0 ± 0.3	62.5 ± 0.3	23.1	4.6	27.7
	75	90.5 ± 0.1	64.3 ± 1	24.2	3.4	27.6
	100	92.9 ± 0.3	65.1 ± 0.2	26.6	2.2	28.8
**P2**	0	82.9 ± 1.4	56.5 ± 1.2	23.8	6.4	30.3
	84	87.5 ± 0.3	63.3 ± 1.1	21.2	5.3	26.6
**P3**	0	90.5 ± 0.3	61.9 ± 0.5	27.7	2.6	30.3
	80	93.6 ± 0.3	65.5 ± 0.5	27.1	1.9	29.0

[a]***E*-P1** (M_n = 13200) for 0% *Z* **P1**. The others are prepared by photoisomerization of ***Z*-P1**.

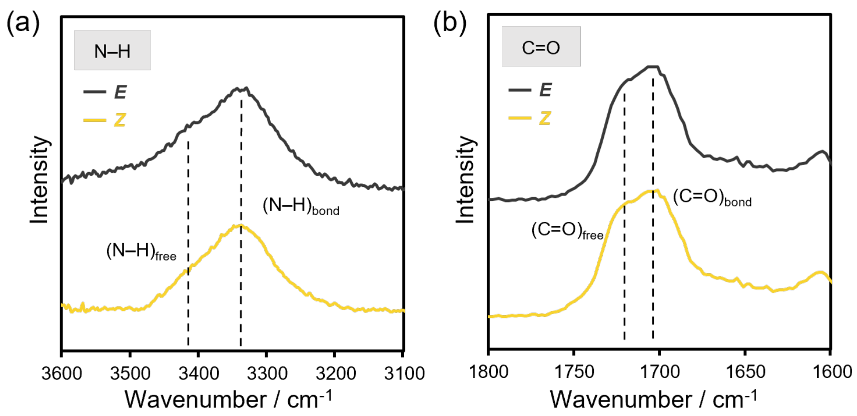

Fig. S70 FTIR spectra of ***E*-P1** (M_n = 13200) and ***Z*-P1** thin films focused on (a) N–H and (b) C=O stretching vibration.

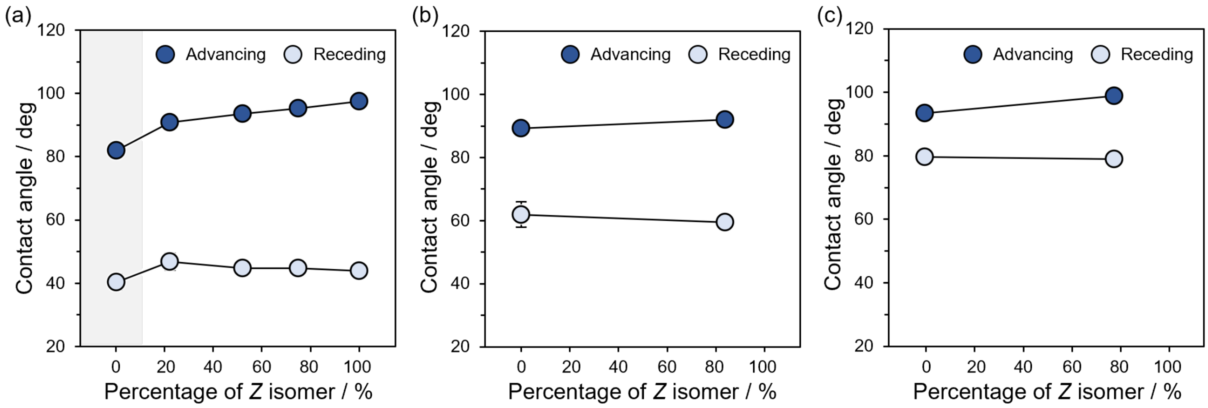

Fig. S71 Dynamic contact angles of water on (a) **P1**, (b) **P2**, and (c) **P3** thin films with different *E*/*Z* ratios (n = 3). Standard deviations are added as error bars but mostly buried. 0% *Z* **P1** highlighted in light gray is ***E*-P1** (M_n = 13200). The others are prepared by photoirradiation of ***Z*-P1**.

Table S7 Dynamic contact angles of water on **P1**, **P2**, and **P3** thin films with different *E*/*Z* ratios (n = 3, mean ± standard deviation) and hysteresis

polymer	*Z* ratio (%)	Dynamic contact angle		Hysteresis
		θ_A	θ_R	
**P1**[a]	0	81.9 ± 0.9	40.4 ± 2.5	41.5
	22	90.8 ± 0.3	46.8 ± 2.8	44.0
	52	93.6 ± 1.1	44.8 ± 0.0	48.8
	75	95.2 ± 1.0	44.8 ± 0.0	50.4
	100	97.4 ± 0.3	43.9 ± 1.3	53.5
**P2**	0	89.3 ± 1.3	61.9 ± 4.0	27.4
	84	92.0 ± 1.8	59.5 ± 2.1	32.5
**P3**	0	93.4 ± 0.8	79.6 ± 2.1	13.8
	80	98.8 ± 1.0	79.0 ± 1.1	19.8

[a]***E*-P1** (M_n = 13200) for 0% *Z* **P1**. The others are prepared by photoisomerization of ***Z*-P1**.

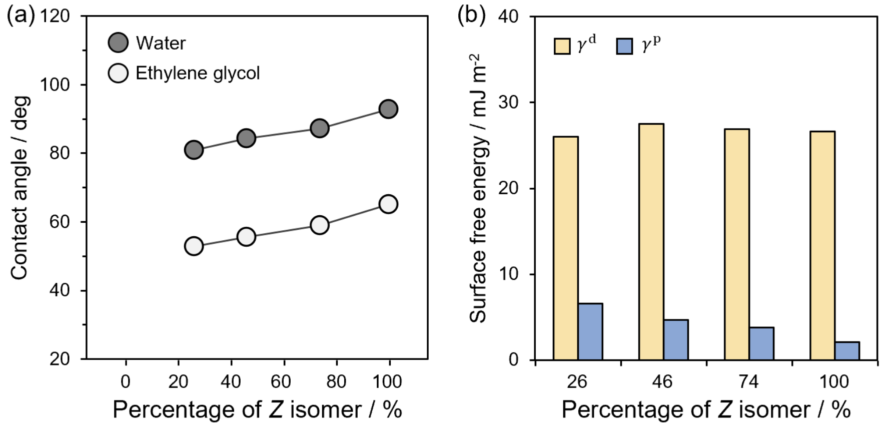

Fig. S72 (a) Static contact angles of water and ethylene glycol on **P1** thin films with different *E*/*Z* ratios prepared by irradiation of ***Z*-P1** thin film with 405 nm light (n = 4) and (b) γ^d and γ^p of surface free energies.

Table S8 Static contact angles (θ_S) of water and ethylene glycol on **P1** thin films with different *E*/*Z* ratios prepared by irradiation of ***Z*-P1** thin film with 405 nm light (n = 4, mean ± standard deviation) and surface free energies

polymer	*Z* ratio (%)	θ_S Water (deg)	θ_S ethylene glycol (deg)	Surface free energy γ^d (mJ m^{-2})	Surface free energy γ^p (mJ m^{-2})	Surface free energy γ (mJ m^{-2})
**P1**	26	81.0 ± 0.5	52.8 ± 0.3	26.0	6.6	32.6
	46	84.4 ± 0.3	55.5 ± 0.3	27.5	4.7	32.2
	74	87.2 ± 0.2	59.0 ± 0.6	26.9	3.8	30.7
	100	92.9 ± 0.3	65.1 ± 0.2	26.6	2.2	28.8

References

1. K. Imato, A. Ishii, N. Kaneda, T. Hidaka, A. Sasaki, I. Imae and Y. Ooyama, *JACS Au*, 2023, **3**, 2458–2466.
2. K. Nomura, P. Chaijaroen and M. M. Abdellatif, *ACS Omega*, 2020, **5**, 18301–18312.
3. K. Imato, A. Sasaki, A. Ishii, T. Hino, N. Kaneda, K. Ohira, I. Imae and Y. Ooyama, *J. Org. Chem.*, 2022, **87**, 15762–15770.
4. Y. Xie, J. Iwata, T. Matsumoto, N. L. Yamada, F. Nemoto, H. Seto and T. Nishino, *Langmuir*, 2022, **38**, 6472–6480.
